# A Photothermal Transducer Regulates Transmembrane Calcium Flow for Synergistic Osteoarthritis Therapy

**DOI:** 10.1002/advs.202520157

**Published:** 2026-03-06

**Authors:** Chenqi Yu, Yang Liu, Kang Kang, Jianfeng Yu, Xiaowei Xia, Yaoge Deng, Yingjie Lu, Huilin Yang, Lisong Li, Wu Xu, Yijian Zhang, Xuesong Zhu

**Affiliations:** ^1^ Department of Orthopaedics The First Affiliated Hospital of Soochow University Soochow University Suzhou China; ^2^ Orthopaedic Institute Medical College Soochow University Suzhou China

**Keywords:** calcium influx, macrophage polarization, osteoarthritis, photothermal therapy, transient receptor potential vanilloid type 4

## Abstract

Hyperthermia is a well‐established physical therapy modality used in orthopedics as an alternative treatment for osteoarthritis (OA). However, the precise cellular‐level interventions and intrinsic mechanisms regulating the joint microenvironment remain poorly understood. In this study, we developed a near‐infrared (NIR)‐responsive nanotransducer composed of selenium‐doped carbon quantum dots attached to black phosphorus nanosheets and coated with a membrane derived from M2 macrophages (M2M), termed M2M@BPSC. Upon NIR stimulation, M2M@BPSC selectively induced mild hyperthermia in macrophages, thereby inhibiting the transient receptor potential vanilloid type 4 (TRPV4) channel to inhibit calcium ion influx and prevent calcium overload‐induced mitochondrial dysfunction. Furthermore, TRPV4 suppression promotes the nuclear translocation of signal transducer and activator of transcription 6 dimers, enhancing the transcriptional activity of early growth response 2. This activates the expression of M2‐polarization‐associated genes. Programmed M2M exerts bidirectional regulatory effects on both chondrocytes and fibroblast‐like synoviocytes, thereby restoring the balance between cartilage extracellular matrix (C‐ECM) degradation and fibrosis ECM (F‐ECM) production. Overall, we identified a thermosensitive ion channel‐targeting strategy that effectively modulates the pathological microenvironment in OA.

## Graphical abstract

Upon NIR irradiation at 808 nm, the activity of TRPV4 was inhibited by M2M@BPSC, which led to reduced calcium influx, mitochondrial protection, and, importantly, the transcriptional activation of the STAT6‐EGR2 pathway. The improved macrophage function indirectly readjusts the equilibrium between C‐ECM and F‐ECM to reconstruct a suitable microenvironment for synergistic therapy in osteoarthritis.

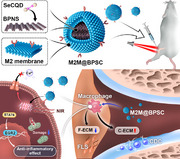

## 1 Introduction

**SCHEME 1:**
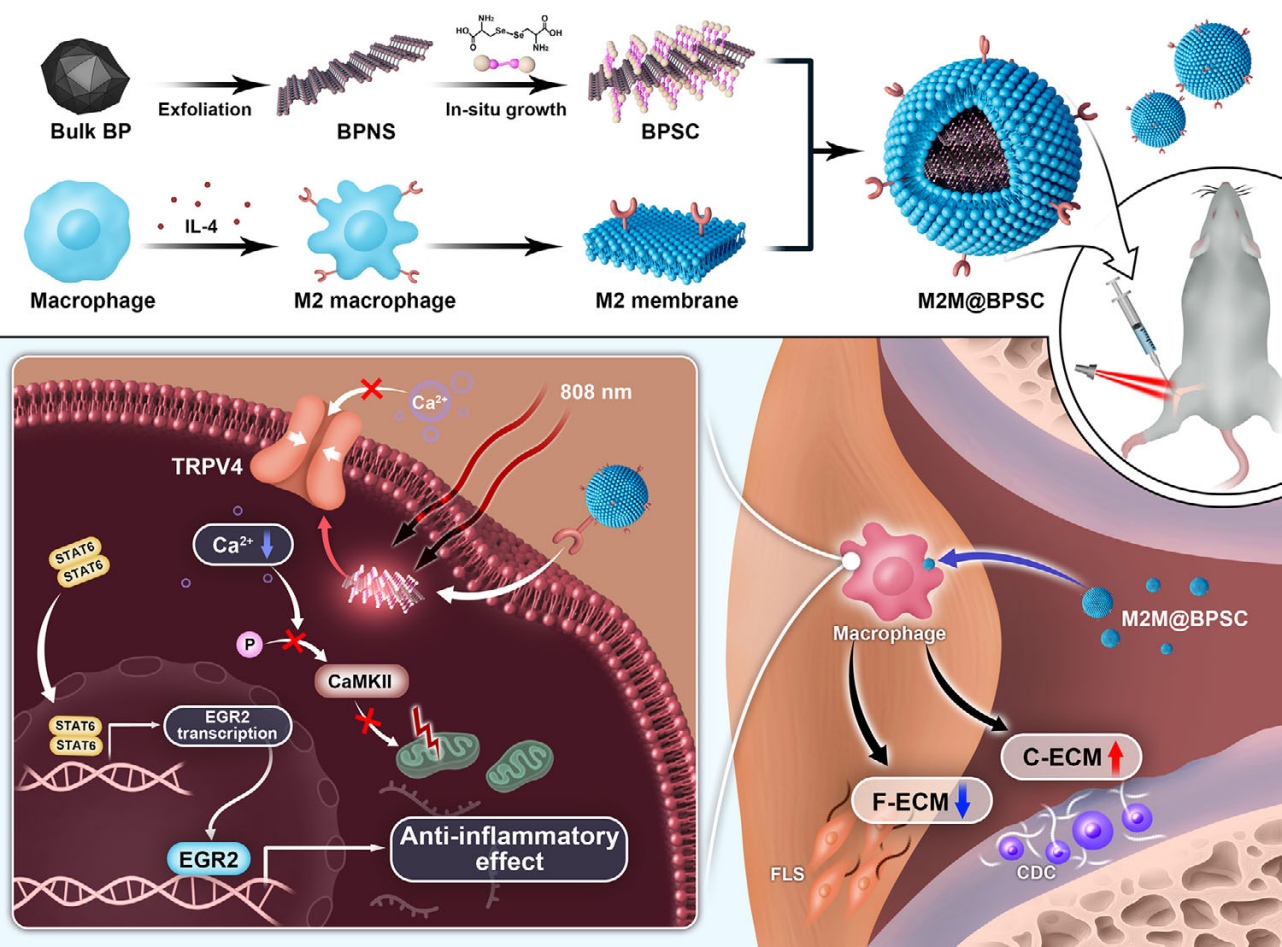
A photothermal transducer activates temperature‐sensitive ion channels for synergistic therapy in OA. M2M@BPSC was synthesized using a combination of liquid‐phase exfoliation, in situ growth techniques, and cell membrane coating. Upon NIR irradiation at 808 nm, TRPV4 activity was inhibited, leading to reduced calcium influx and mitochondrial protection. It enhanced M2 polarization of macrophages through the signal transducer and activator of transcription 6‐early growth response 2 (STAT6‐EGR2) signaling pathway. Improved macrophage function indirectly led to decreased F‐ECM and increased C‐ECM synthesis, thereby reversing the pathological microenvironment associated with synovial inflammation and cartilage degradation.

Musculoskeletal diseases have emerged as the leading cause of disability worldwide due to population aging [1]. Among them, osteoarthritis (OA), the most prevalent degenerative joint disorder, has indicated a marked increase in incidence, prevalence, and disability‐adjusted life years over the past three decades [2]. Traditionally, OA progression has been attributed primarily to cartilage degeneration, driven by chondrocyte apoptosis and breakdown of the cartilage extracellular matrix (C‐ECM) [3]. Our recent studies demonstrate that enhancing chondrocyte function can effectively mitigate the OA phenotype by boosting energy production [4], suppressing free radical damage [5], and restoring autophagy flux [6]. However, advances in the understanding of OA pathogenesis have revealed that non‐chondrocyte cells, including fibroblast‐like synoviocytes (FLS), synovial macrophages [7], and various bone‐derived cells (osteoclasts, osteoblasts, osteocytes, and stem cells), also play critical roles in disease development—the CX3CR1^+^ epithelial‐like lining macrophages act as a critical physical barrier, limiting inflammatory responses in arthritis [8]. GATD3A deficiency‐induced senescence in FLS impairs the mitochondrial tricarboxylic acid (TCA) cycle, thereby accelerating OA progression [9]. Clinical evidence also indicates that dysregulated immune‐regulatory macrophages and fibroblast subsets are closely related to increased pain in patients with knee OA [10]. Targeting anti‐inflammatory macrophages and non‐pathological fibroblasts to reduce synovitis has the potential to alleviate OA‐related tissue damage [11]. Moreover, synovial macrophages can indirectly impair the function of other cell types, such as chondrocytes, by secreting specific proteins, thereby disrupting C‐ECM integrity and perpetuating a vicious “inflammation–degradation” cycle that accelerates OA progression [12]. Therefore, restoring impaired macrophage function to interrupt this pathological cycle offers a novel and promising therapeutic strategy for OA treatment (Scheme 1).

Contrary to traditional therapeutic approaches, mild‐temperature photothermal therapy (PTT) offers non‐invasive and highly selective treatment advantages [13], making it particularly suitable for the localized treatment of joint diseases [14]. Precise regulation of cellular temperature has been indicated to promote osteogenic differentiation [15]. PTT‐induced activation of heat shock protein 70 [16] or suppression of reactive oxygen species (ROS) can facilitate cartilage regeneration [17]. Black phosphorus (BP), a photothermal material with a high photothermal conversion efficiency, can be irradiated with near‐infrared (NIR) light to generate localized heat [18]. In particular, BP nanosheets (BPNS) are a newly investigated class of 2D nanomaterials that exhibit a high specific surface area, tunable direct bandgap, and consequently superior photothermal properties [19]. BPNS converts light energy into heat to initiate biomineralization, effectively blocking the dentinal tubules and alleviating dentin hypersensitivity [20]. In OA therapy, BPNS has demonstrated robust ROS‐scavenging capabilities that promote C‐ECM synthesis and subchondral bone remodeling [21]. However, the antioxidant capacity of natural BPNS is limited and cannot completely counteract the overactivated oxidative stress in the late‐stage OA microenvironment. Selenium (Se) is an essential dietary trace element for humans, and Se‐doped carbon quantum dots (SeCQDs) are a novel type of semiconductor quantum dots incorporating Se elements [22]. They offer superior biocompatibility compared to heavy metal ion‐based quantum dots, including lead selenide or silver selenide [23]. Importantly, SeCQDs enhance endogenous antioxidant defenses, thereby protecting tissues from oxidative damage [24]. However, neither BPNS nor SeCQDs can achieve targeted therapy for specific cells or tissues, potentially leading to unnecessary side effects. To address this, cell membrane coating technology is widely used to enhance the cell‐targeting capabilities of nanoparticles via homologous targeting‐binding mechanisms [25]. Macrophage membrane (MM)‐coated nanorods can be actively internalized by resident foamy macrophages for atherosclerosis treatment [26]. Furthermore, MM cells obtained after specific pretreatment can be engineered to confer additional properties such as enhanced dual‐targeting capabilities or anti‐inflammatory effects [27].

Herein, we designed a novel multifunctional photothermal transducer, M2M@BPSC, to disrupt cartilage degeneration and synovial hyperplasia, thereby mitigating OA progression. First, 2D BPNS were fabricated via liquid‐phase exfoliation (photothermal cues). Subsequently, SeCQDs‐attached BPSC was synthesized using an in situ growth approach (antioxidant element). Finally, a membrane derived from interleukin 4 (IL‐4)‐induced M2 macrophages was used to coat BPSC, leading to the formation of M2M@BPSC (targeting guidance). Importantly, M2M@BPSC effectively promotes anti‐inflammatory M2 polarization of synovial macrophages through its excellent targeting capability, strong antioxidative properties, and enhancement of mitochondrial function. Mechanistically, the mild photothermal effect induced by M2M@BPSC inhibits the TRPV4 ion channel on the cell membrane, thereby reducing extracellular calcium influx. This reduction leads to STAT6 translocation into the nucleus, where it specifically binds to the EGR2 promoter to activate transcription, ultimately contributing to the anti‐inflammatory phenotype. By modulating macrophage paracrine activity, M2M@BPSC indirectly promoted C‐ECM synthesis and suppressed fibrotic ECM (F‐ECM) deposition, thereby synergistically enhancing joint mobility recovery. Overall, our proposed photothermal transducer presents a promising new therapeutic option for the treatment of OA.

## 2 Results and Discussion

### 2.1 Preparation and Characterization of BPSC

**FIGURE 1:**
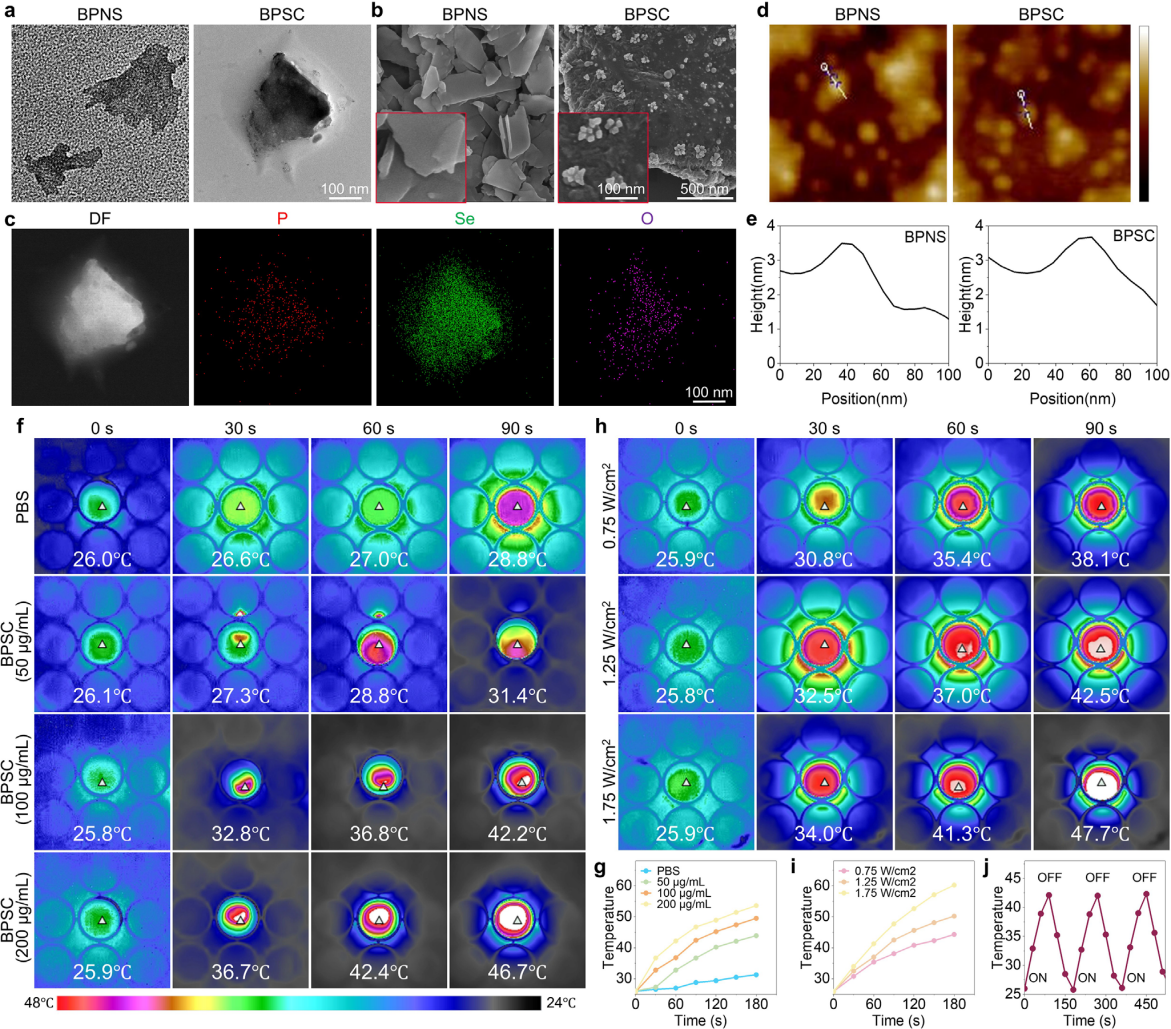
Synthesis and multi‐scale characterization of BPSC. (a) TEM images of BPNS and BPSC. (b) SEM images of BPNS and BPSC. (c) STEM image and corresponding EDX elemental mappings (P, Se, O) of BPSC. (d) AFM images of BPNS and BPSC. (e) Height profiles along dashed lines in (d). (f) Real‐time thermal imaging of BPSC suspensions under NIR irradiation (808 nm, 1.25 W/cm^2^) at varied concentrations. (g) In vitro temperature profiles of BPSC suspensions at indicated concentrations (NIR: 808 nm, 1.25 W/cm^2^). (h) Real‐time thermal imaging of BPSC suspensions (100 µg/mL) under different NIR power densities (808 nm). (i) In vitro temperature profiles of BPSC at varied NIR power densities (NIR: 808 nm). (j) Cyclic photothermal stability of BPSC suspensions (100 µg/mL) under ON/OFF NIR irradiation (808 nm, 1.25 W/cm^2^; 3 cycles).

First, we prepared 2D BPNS using a conventional liquid‐phase exfoliation method. These BPNS were subsequently combined with SeCQDs through an in situ growth method to synthesize BPNS plus SeCQDs (BPSC). Transmission electron microscopy (TEM) images indicated the formation of ultra‐thin nanosheets of BPNS and BPSC (Figure 1a). Scanning electron microscopy revealed that SeCQDs were deposited on the surface of the BPNSs in the BPSC group (Figure 1b). The morphology and specific crystal faces of BP were identified, with nanoparticle pores approximately 150 nm in size (Figure S1). The interplanar spacing of 0.34 nm identified in the prepared BPNS and BPSC corresponds to the (0 2 1) crystallographic plane of BP (Figure S2) [28]. Elemental composition analysis revealed the presence of intrinsic P and the introduction of oxygen and Se into the BPSC (Figure 1c; Figure S3). As measured by atomic force microscopy, the thickness of BPNS or BPSC was approximately 3 nm (Figure 1d,e), which generally aligns with previously reported values [29]. Raman spectra additionally revealed distinct and complementary vibrational peaks for the nanosheets (Figure S4a) [30]. UV–vis absorption spectroscopy revealed a new characteristic absorption peak of SeCQDs at approximately 260 nm in BPSC compared to BPNS (Figure S4b) [31]. These experimental findings corroborate the successful preparation of nanoscale BPSC. Based on the photothermal properties of BP, we investigated the temperature changes in a cell culture plate using high‐precision digital temperature sensors at varying concentrations of BPSC and different NIR irradiation intensities. The infrared thermal imaging results indicated that the temperature increased gradually with increasing BPSC concentration (Figure 1f,g). Similarly, increasing the NIR irradiation intensity led to a corresponding increase in the temperature (Figure 1h,i). Notably, the introduction of SeCQDs did not diminish the photothermal effect of BPNS (Figure S5). The photothermal stability of BPSC was further assessed through four cycles of heating and natural cooling, demonstrating its high photothermal stability (Figure 1j). Excessively high temperatures (>45°C) in traditional PTT may harm healthy cells and tissues. It is primarily applied in anti‐tumor [32] or antibacterial therapies [33] but is unsuitable for chronic degenerative disorders such as OA. To attain a moderate photothermal therapeutic effect (∼42°C) while avoiding potential side effects, and considering the practical photothermal conversion efficiency of BPSC, we selected an optimal treatment protocol (100 µg/mL and 1.25 W/cm^2^) for subsequent experiments. In summary, modified BPSC nanosheets were successfully fabricated and demonstrated excellent photothermal performance.

### 2.2 BPSC+NIR Induced Macrophage Reprogramming and Improved Mitochondrial Function

**FIGURE 2:**
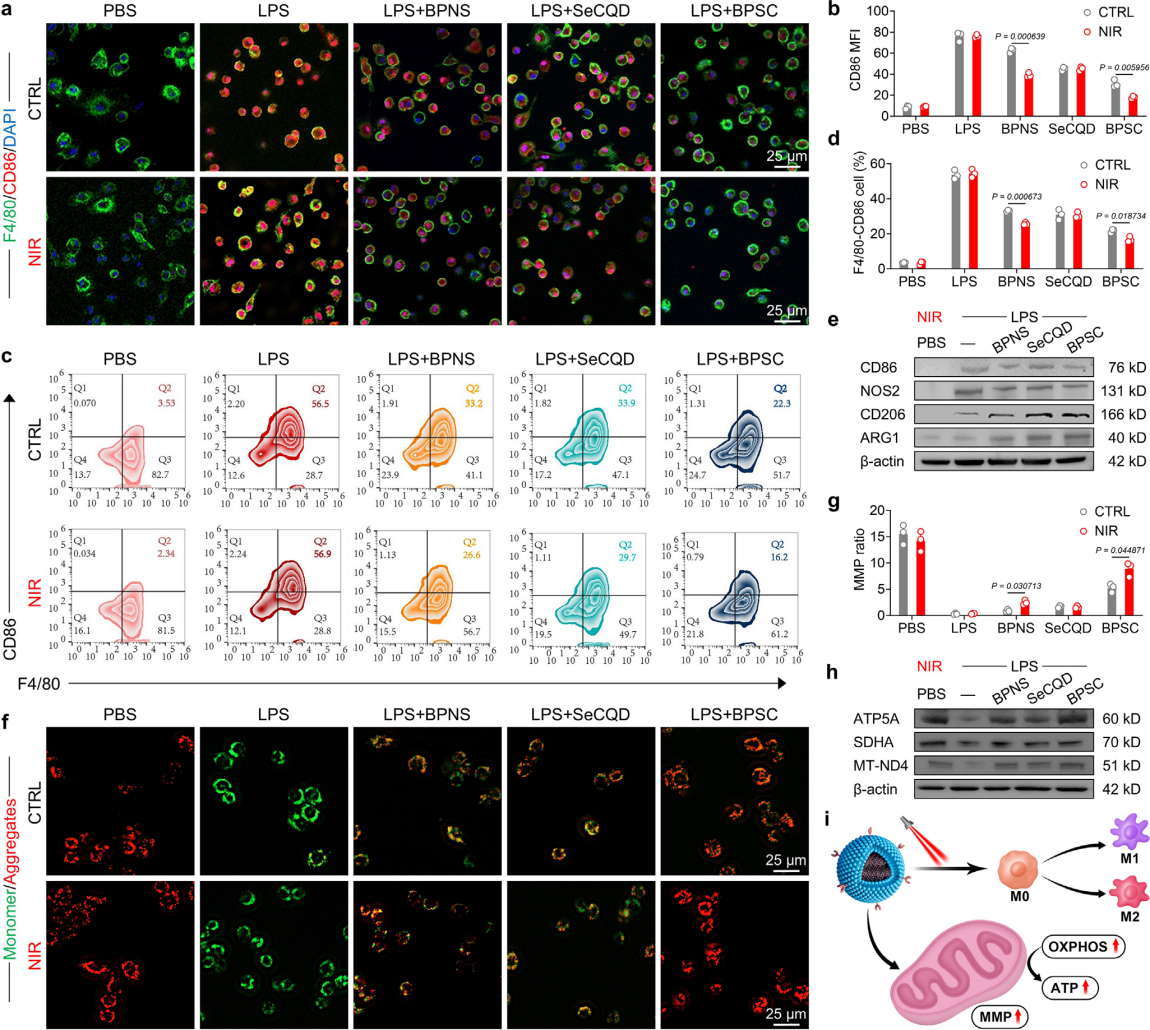
Photothermal‐driven macrophage reprogramming via BPSC: boosting M2 polarization and mitochondrial function (a,b) Immunofluorescence staining and quantification of CD86 in BMDM under control treatment and near‐infrared heat treatment (NIR: 808 nm, 1.25 W/cm^2^, 90 s) (n = 3). (c,d) Representative flow cytometry plots and quantification of M1 macrophages (CD86+ and F4/80+ cells) under control treatment and near‐infrared heat treatment (NIR: 808 nm, 1.25 W/cm^2^, 90 s) (n = 3). (e) Representative western blot bands of macrophage polarization in BMDM at different conditions under near‐infrared heat treatment (NIR: 808 nm, 1.25 W/cm^2^, 90 s) (n = 3). (f,g) JC‐1 staining and quantification for mitochondrial membrane potential (ΔΨm) in BMDM under near‐infrared heat treatment (NIR: 808 nm, 1.25 W/cm^2^, 90 s) (n = 3). (h) Representative western blot bands of mitochondrial function in BMDM at different conditions under near‐infrared heat treatment (NIR: 808 nm, 1.25 W/cm^2^, 90 s) (n = 3). (i) BPSC+NIR induced macrophage reprogramming and enhanced mitochondrial function.

In the in vitro biosafety assessment, cell proliferation and cytotoxicity assays demonstrated that BPSC did not exert cytotoxic effects on bone marrow‐derived macrophages (Figure S6). In LPS‐induced M1 polarization, BPSC treatment reduced CD86 expression while improving CD206 levels in F4/80‐positive macrophages. Among NIR light exposure groups, the beneficial effects on macrophage polarization were significantly enhanced in BPNS and BPSC groups, corroborating the photothermal therapeutic efficacy of BP (Figure 2a,b; Figure S7a,b). Flow cytometric analysis further demonstrated that BPSC promoted the reprogramming of inflammatory macrophages from M1 to M2 phenotype, especially under NIR intervention (Figure 2c,d; Figure S7c,d). Furthermore, the transcriptional and translational levels of M1‐related activation markers, including CD80 and iNOS, were suppressed following BPSC treatment, whereas M2‐related markers, including Arg1 and CD209 [34], were upregulated (Figure 2e; Figure S8). Mitochondrial function plays a key role in maintaining the macrophage regulatory phenotype. Mitochondrial dysfunction in macrophages caused by Ndufs4 deletion can exacerbate the inflammatory response and impair myocardial infarction repair [35]. Conversely, the restoration of mitochondrial homeostasis in synovial macrophages helps rebalance the M1/M2 phenotypic ratio and alleviates inflammation in OA [36]. Accordingly, we evaluated the potential effects of BPSC combined with NIR on mitochondrial metabolism and observed a significant restoration of the mitochondrial membrane potential (MMP) (Figure 2f,g). Owing to the robust antioxidant capacity of SeCQDs, extracellular free radicals, such as superoxide anions and hydroxyl radicals, were effectively scavenged by SeCQDs and BPSC (Figure S9) [37]. Additionally, MitoSOX and DCFDA staining results demonstrated that NIR‐triggered BPSC treatment significantly reduced intracellular and mitochondrial ROS‐induced damage (Figure S10a–d). Furthermore, mitochondria serve as central hubs for cellular respiration and energy metabolism. M1 pro‐inflammatory macrophages primarily rely on glycolysis for energy production [38]. Conversely, supplementation with α‐ketoglutarate, a crucial metabolite in the TCA cycle, promotes macrophage activation toward the M2 phenotype [39]. Metabolic flux analysis using Seahorse technology revealed that BPSC+NIR treatment enhanced basal, maximal, and ATP production, indicating improved mitochondrial aerobic respiration in macrophages (Figure S11). Moreover, BPSC+NIR upregulated the expression of subunits of mitochondrial respiratory chain complexes, including ATP5A, SDHA, and MT‐ND4 (Figure 2h; Figure S12). Collectively, these findings demonstrate that BPSC combined with NIR restores mitochondrial function in macrophages, thereby promoting anti‐inflammatory M2 polarization (Figure 2i).

### 2.3 BPSC‐Induced PTT Suppressed the TRPV4 Ion Channel Activity to Limit the Excessive Calcium Influx

**FIGURE 3:**
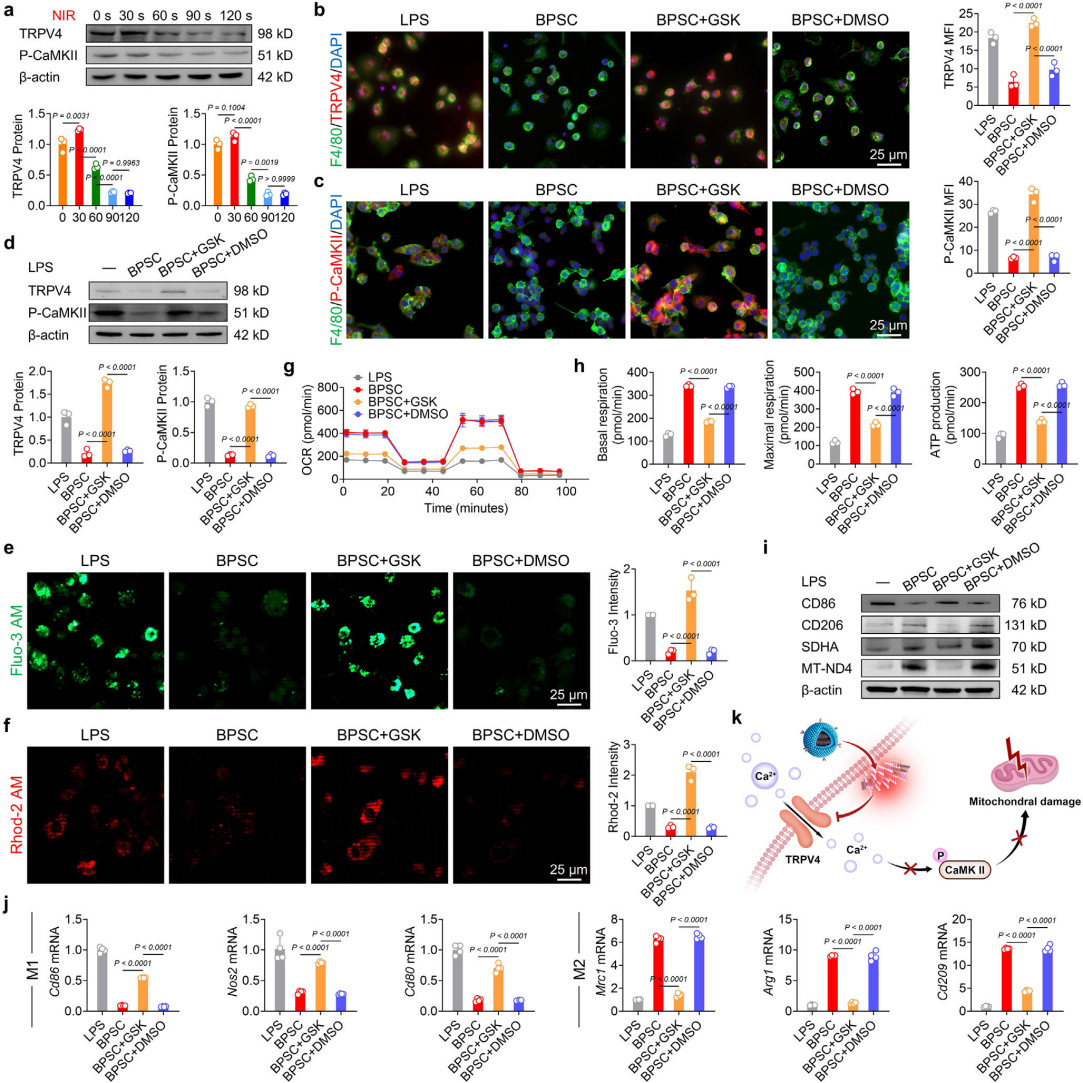
BPSC‐mediated photothermal therapy (PTT) attenuated the TRPV4 ion channel, reducing calcium influx. (a) Representative western blot bands and quantification of TRPV4 and P‐CaMKII expression in BMDM at varied exposure times under near‐infrared heat treatment (NIR: 808 nm, 1.25 W/cm^2^) (n = 3). (b,c) Immunofluorescence staining and quantification of TRPV4 and P‐CaMKII in BMDM under near‐infrared heat treatment (NIR: 808 nm, 1.25 W/cm^2^, 90 s) (n = 3). (d) Representative western blot bands and quantification of TRPV4 and P‐CaMKII expression in BMDM at LPS conditions under near‐infrared heat treatment (NIR: 808 nm, 1.25 W/cm^2^) (n = 3). (e,f) Representative images and quantitative analysis of intracellular (Fluo‐3 AM, green) and mitochondrial Ca^2+^ localization (Rhod‐2 AM, red) (n = 3). (g) Oxygen consumption rate (OCR) of BMDM under near‐infrared heat treatment (NIR: 808 nm, 1.25 W/cm^2^, 90 s) (n = 3). (h) Oxygen consumption rate (OCR) profiles showing: basal respiration, ATP production, and maximal respiration (n = 3). (i) Representative western blot bands of macrophage polarization and mitochondrial function in BMDM at different conditions under near‐infrared heat treatment (NIR: 808 nm, 1.25 W/cm^2^, 90 s) (n = 3). (j) qPCR analysis of CD86, iNOS, CD80, MRC1, Arg1, and CD209 mRNA levels in BMDM at different conditions under near‐infrared heat treatment (NIR: 808 nm, 1.25 W/cm^2^, 90 s) (n = 4). (k) BPSC‐induced PTT suppressed TRPV4 ion channel activity to limit excessive calcium influx.

Based on the aforementioned functional results, we further investigated the molecular mechanism by which the photothermal effect induced by BPSC combined with NIR irradiation modulates macrophage phenotypic polarization. The transient receptor potential vanilloid channel 4 (TRPV4) is a thermosensitive ion channel that plays a key role in skeletal development [40]. TRPV4 remains activated within the physiological temperature range of 27°C to 42°C [41], whereas excessively high temperatures may attenuate channel activity through adaptive responses or channel desensitization [42]. To verify this conjecture, macrophages were exposed to BPSC combined with NIR stimuli for different durations. Over time, TRPV4 expression gradually declined, suggesting that persistent heating desensitizes TRPV4 and reduces its expression (Figure 3a). Patch‐clamp experiments demonstrated that treatment with BPSC+NIR reduced TRPV4 channel activity (Figure S13). Furthermore, we conducted continuous whole‐cell voltage‐clamp recordings in the same cell both before and after treatment with BPSC+NIR at different time points. Our results demonstrated that within the same cell, TRPV4 currents exhibited a multiphasic response to photothermal stimulation: potentiation at 30 s, a decline from 30 to 60 s, and significant suppression by 90 s, which indicated progressive desensitization (Figure S14). In response to temperature changes, TRPV4 primarily regulates calcium influx [43]. Our findings indicated that, in response to the multiphasic alteration of TRPV4, the phospho‐Ca^2+^/calmodulin (CaM)‐dependent protein kinase II (P‐CaMKII) exhibited a similar trend of rising first and then falling after PTT (Figure 3a) [44]. To further elucidate the critical role of TRPV4, a specific agonist (GSK1016790A) was used to activate TRPV4 in macrophages [45]. Immunofluorescent staining demonstrated that GSK1016790A improved TRPV4 expression on the plasma membrane and elevated the cytoplasmic levels of P‐CaMKII (Figure 3b,c). Protein quantification analysis confirmed the strong activation of TRPV4 and its downstream target P‐CaMKII by BPSC plus NIR (Figure 3d). Calcium fluorescence probe analysis using Fluo‐3 AM and Rhod‐2 AM demonstrated that PTT reduced intracellular calcium concentration, whereas TRPV4 activation promoted calcium influx, ultimately leading to cytosolic calcium overload (Figure 3e,f). Calcium overload, resulting from excessive calcium influx, can lead to the loss of MMP, increased oxidative damage, and ultimately, mitochondrial dysfunction [46]. Mitochondrial calcium overload also contributes to the development of pathogenic programmed necrosis in macrophages infected with mycobacteria [47]. To investigate the effects of TRPV4‐mediated calcium ion transport on mitochondrial function and the polarization phenotype of macrophages. Oxygen consumption rate analysis indicated that the enhancement of aerobic respiration and energy metabolism induced by BPSC combined with NIR was negated by treatment with GSK1016790A (Figure 3g,h). Similarly, the expression levels of respiratory chain complexes were downregulated (Figure 3i; Figure S15), and the endogenous antioxidant capacity was reduced in macrophages treated with GSK1016790A (Figure S16). In the macrophage polarization analysis, inhibiting TRPV4 with GSK1016790A reverses the anti‐inflammatory M2 phenotype back to a pro‐inflammatory M1 phenotype (Figure 3j; Figure S17a–c). Subsequently, to determine the critical role of TRPV4 in regulating macrophage polarization and calcium transmembrane transport, macrophages were transfected with TRPV4‐targeting siRNA to specifically knock down the TRPV4 protein (Figure S18). Interestingly, BPSC+NIR exerted a similar beneficial effect to siTRPV4 treatment in promoting M2 anti‐inflammatory polarization (Figure S19a–c) and restricting calcium influx (Figure S20). This suggests the potential regulatory effect of BPSC‐induced PTT on TRPV4, for example, acting as a TRPV4 antagonist. Overall, the BPSC‐induced photothermal effect inactivates the TRPV4 ion channel, thereby restricting calcium influx and contributing to mitochondrial protection (Figure 3k).

### 2.4 BPSC‐Induced PTT Promoted Anti‐Inflammatory Polarization Through the STAT6‐EGR2 Signaling Pathway

**FIGURE 4:**
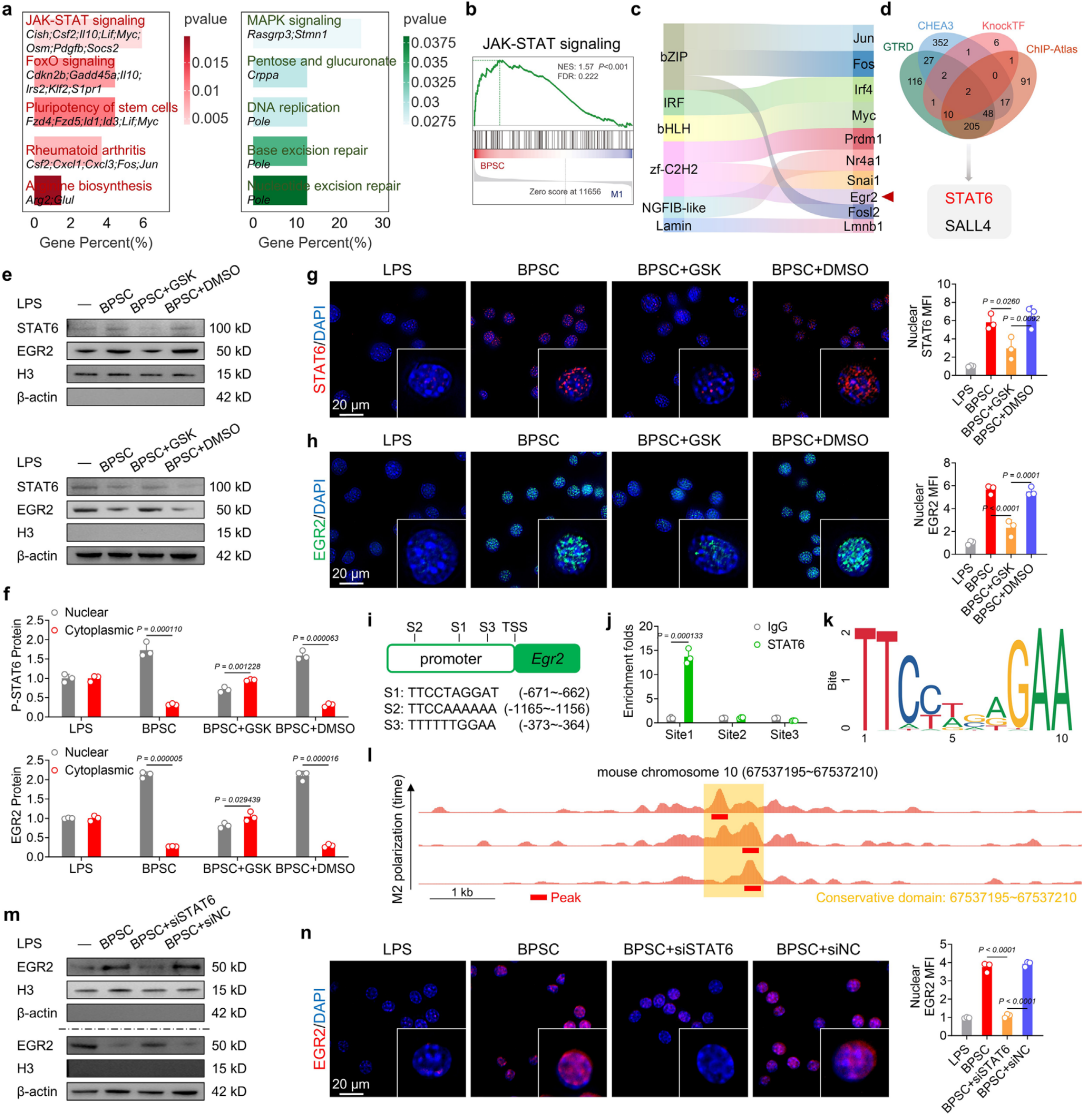
BPSC‐mediated photothermal activation drove M2 macrophage polarization via the STAT6‐dependent EGR2 signaling axis. (a) KEGG analysis of BMDM under control treatment or near‐infrared heat treatment with BPSC. (b) GSEA plot comparing CTRL and BPSC+NIR groups. (c) Sankey diagram showing differential expression flow from transcription factor families to individual transcription factors. (d) Venn diagram of the overlapping in the “regulation of EGR2” gene sets from the GTED, CHEA3, KnockTF, and ChIP‐Atlas databases. (e,f) Representative western blot bands and quantification of cytoplasmic and nuclear STAT6 and EGR2 expression in BMDM at different conditions under near‐infrared heat treatment (NIR: 808 nm, 1.25 W/cm^2^, 90 s) (n = 3). (g,h) Immunofluorescence staining and quantification of nuclear STAT6 and EGR2 in BMDM at different conditions under control treatment and near‐infrared heat treatment (NIR: 808 nm, 1.25 W/cm^2^, 90 s) (n = 3). (i) Schematic diagram of three potential STAT6 binding sites in the promoter of EGR2 predicted by the JASPAR website. (j) ChIP‐qPCR quantification of STAT6 occupancy at three sites in the EGR2 promoter in BMDMs: CTRL vs. BPSC+NIR groups (n = 3). (k) Consensus STAT6‐binding motif in the mammalian EGR2 promoter. (l) Anti‐STAT6 ChIP‐seq binding in the C57BL6/J mouse EGR2 gene (GSE38377) visualized via UCSC Genome Browser, highlighting the putative conserved STAT6 binding site within the mammalian EGR2 gene. The red box represents the binding site “Peak.” (m) Representative western blot bands and quantification of cytoplasmic and nuclear STAT6 and EGR2 expression in BMDMs at different conditions under near‐infrared heat treatment (NIR: 808 nm, 1.25 W/cm^2^, 90 s). (n) Immunofluorescence staining and quantification of nuclear EGR2 in BMDMs at different conditions under control treatment and near‐infrared heat treatment (NIR: 808 nm, 1.25 W/cm^2^, 90 s).

To further elucidate the underlying mechanisms by which BPSC‐mediated PTT regulates macrophage function, M1‐polarized macrophages treated with or without NIR+BPSC were subjected to RNA sequencing. Using a standard threshold for screening (q< 0.05 and |logFC| >1), 323 upregulated and 16 downregulated genes were identified (Figure S21). Gene Ontology enrichment analysis indicated that biological processes related to transcriptional regulation, including gene expression, positive regulation of transcription by RNA polymerase II, and transcription regulator complex formation, were enhanced (Figure S22). KEGG enrichment analysis revealed that BPSC treatment upregulated JAK‐STAT and FoxO signaling pathways and stem cell pluripotency, whereas the MAPK signaling pathway was downregulated (Figure 4a). Additional gene set enrichment analysis revealed that BPSC treatment increased JAK‐STAT signaling pathway activity in macrophages (Figure 4b). Accordingly, we considered that STAT‐related transcriptional regulation warranted further investigation. Based on the Sankey diagram of the top 10 differentially expressed transcription factors (TFs), six calcium ion‐related potential TFs (Nr4a1 [48], Myc [49], Jun [50], Fos [51], Irf4 [52], and Egr2 [53]) were identified (Figure 4c). Functional experiments verified that EGR2 is a crucial TF involved in the response to BPSC+NIR and TRPV4 activation, which has also been reported to be strongly correlated with macrophage polarization (Figure S23) [54]. To determine the relationship between STAT signaling and EGR2, we analyzed data from the ChIP‐X Enrichment Analysis 3, Gene Transcription Regulation Database, Knock TF, and ChIP‐Atlas, and identified STAT6 and SALL4 as potential upstream regulators of EGR2 (Figure 4d). Subsequent model‐based analysis of ChIP‐Seq binding scores confirmed the crucial role of STAT6 as a transcriptional modulator of EGR2 (Figure S24). The nucleocytoplasmic separation assay revealed that BPSC treatment facilitated the nuclear translocation of STAT6; however, this effect was abolished upon TRPV4 reactivation (Figure 4e,f). Correspondingly, either the BPSC treatment or the silencing of TRPV4 enhanced STAT6 phosphorylation, while the activation of TRPV4 weakened this effect (Figure S25). This dynamic distribution of STAT6 and EGR2 was further confirmed by immunofluorescence staining (Figure 4g,h). Three candidate binding sites were identified to predict promoter activity using the JASPAR database (Figure 4i). ChIP‐PCR results demonstrated that site 1 (TTCCTAGGAT) was the primary binding site for the transcription factor STAT6 at the EGR2 promoter (Figure 4j,k), which is also conserved across multiple species (Figure S26). Finally, we re‐analyzed a ChIP‐Seq dataset on IL‐4‐induced M2 polarization in macrophages with extended treatment time [55]. This analysis further validated the presence of STAT6‐binding sites in the EGR2 promoter region (Figure 4l). To further determine the significance of the TRPV4‐Ca^2^^+^‐STAT6‐EGR2 axis, macrophages were transfected with specific‐siRNA to silence STAT6 expression (Figure S27). The siSTAT6 treatment partially reversed the protective effects of BPSC treatment on the phenotypic switching of macrophages (Figure S28a–c). Immunofluorescence and nucleocytoplasmic separation assays demonstrated that the suppression of STAT6 resulted in a reduction of EGR2 translocation to the nucleus, a decline in the fluorescence intensity within the nucleus, and thus a decrease in mRNA expression (Figure 4m,n; Figure S29a,b). The above results demonstrated the crucial role of STAT6 in sustaining the pro‐inflammatory effect on macrophage polarization, and also the dependence of EGR2 induction on STAT6. Collectively, these findings demonstrate that BPSC‐induced PTT promotes M2 polarization by activating the STAT6‐EGR2 signaling pathway.

### 2.5 BPSC‐Induced PTT Indirectly Prevented C‐ECM Degradation and Fibroblastic Hyperplasia

**FIGURE 5:**
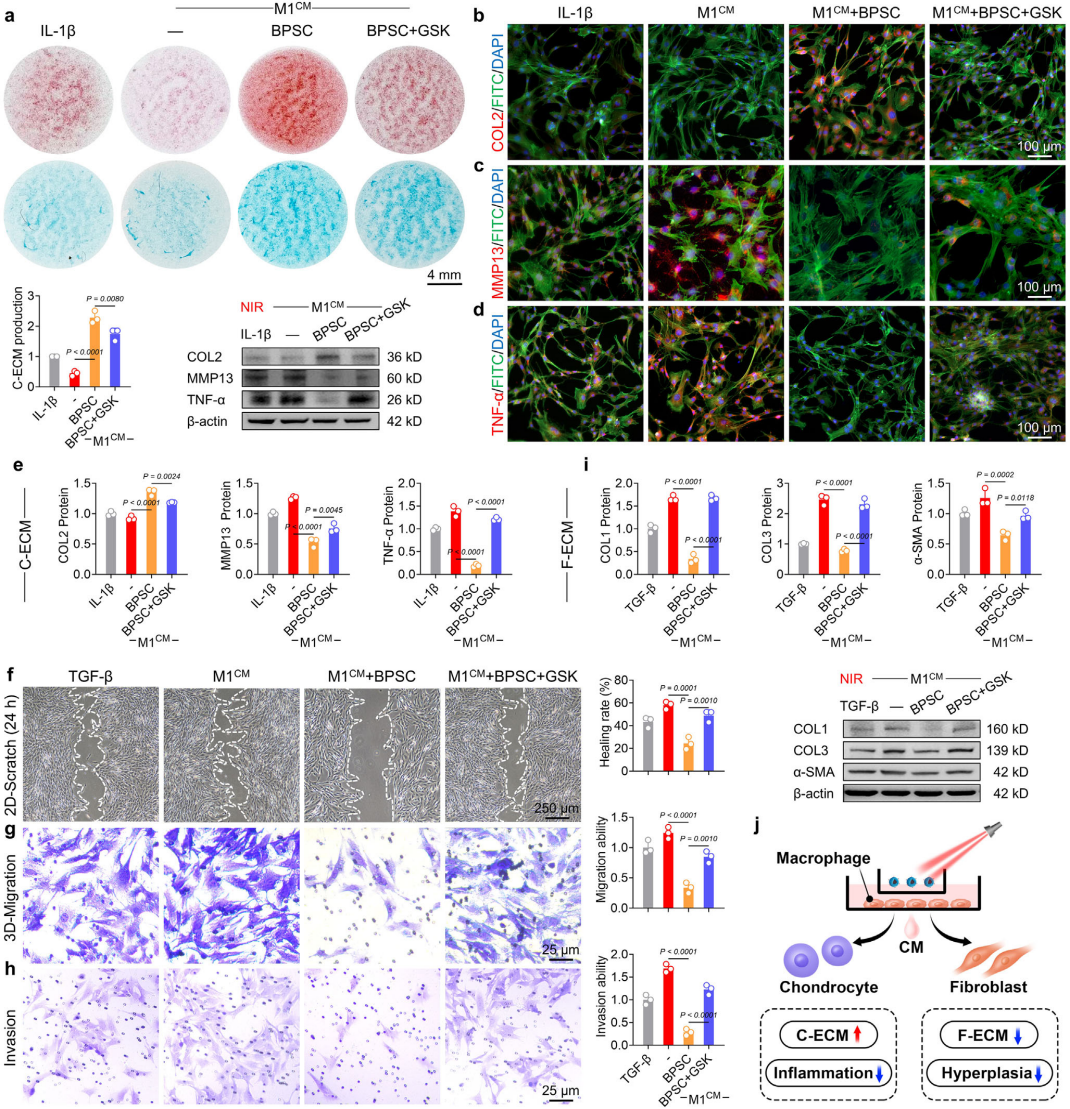
BPSC photothermal therapy suppressed cartilage degradation and synovial hyperplasia via TRPV4/Ca^2+^ modulation. (a) Alcian blue staining and quantitative analysis. (b–d) Immunofluorescence staining of Col2, MMP13, and TNF‐α in murine chondrocytes cultured with macrophage‐conditioned media from distinct polarization states (n = 3). (e) Representative western blot bands and quantification of COL2, MMP13, and TNF‐α expression in murine chondrocytes cultured with macrophage‐conditioned media from distinct polarization states (n = 3). (f) Representative scratch wound healing assay and quantitative analysis of murine fibroblasts cultured with macrophage‐conditioned media from distinct polarization states with TGFβ treatment (n = 3). (g,h) Invasion and migration images and quantitative analysis of murine fibroblasts quantified by Transwell assays. (i) Representative western blot bands and quantification of COL1, COL3, and α‐SMA expression in murine chondrocytes cultured with macrophage‐conditioned media from distinct polarization states (n = 3). (j) Graphic abstract: BPSC‐induced PTT indirectly prevented C‐ECM degradation and fibroblastic hyperplasia.

Macrophages can communicate with target cells via paracrine signaling by secreting various factors. In the joint microenvironment, synovial macrophages indirectly regulate chondrocytes, FLS, osteoblasts, osteoclasts, and osteocytes, thereby influencing the process of joint degeneration. During OA, cartilage degeneration resulting from impaired C‐ECM secretion by chondrocytes [56] and synovial hyperplasia driven by excessive F‐ECM production by FLS [57] are crucial pathogenic factors. Based on this, mouse articular chondrocytes were isolated and treated with various macrophage‐conditioned media (CMs), and the recognized pro‐inflammatory cytokine IL‐1β served as the positive control [58]. ECM results indicated that BPSC‐mediated PTT promoted C‐ECM production; however, this effect was attenuated by the reactivation of the TRPV4 channel (Figure 5a). Immunofluorescence staining demonstrated that BPSC combined with NIR treatment improved the expression of collagen type II (COL2) (Figure 5b), reduced the level of matrix metallopeptidase 13 in chondrocytes (Figure 5c), and reduced the expression of the inflammatory mediator tumor necrosis factor‐alpha (Figure 5d; Figure S30). Follow‐up messenger ribonucleic acid and protein experiments indicated that PTT restored the C‐ECM balance and partially alleviated the inflammatory response via the TRPV4 signaling pathway (Figure 5e; Figure S31a). Second, a mouse fibroblast‐like synoviocyte cell line (CP‐M323) was treated with the same macrophage‐CM, using transforming growth factor β, a classical profibrogenic cytokine, serving as a positive control [59]. Both 2D scratch and 3D transwell migration assays demonstrated that BPSC+NIR significantly inhibited FLS migration, whereas this effect was reversed by GSK1016790A (Figure 5f,g; Figure S32). A further chamber Matrigel invasion assay demonstrated that under ECM‐mimicking conditions, PTT suppressed the invasive capacity of FLS. In contrast, it was enhanced in the presence of a TRPV4 activator (Figure 5h). Quantitative analysis revealed that the expression of F‐ECM markers, including COL1, COL3, and actin alpha 2, was suppressed via the photothermal signal‐TRPV4‐calcium pathway (Figure 5i; Figure S31b). Notably, BPSC+NIR regulated the macrophage phenotype to indirectly protect the homeostasis of C‐ECM and F‐ECM in a paracrine manner (Figure 5j).

### 2.6 M2M@BPSC+NIR Improved Pathological Phenotype and Motor Function in the Post‐Traumatic OA Mice Model

**FIGURE 6:**
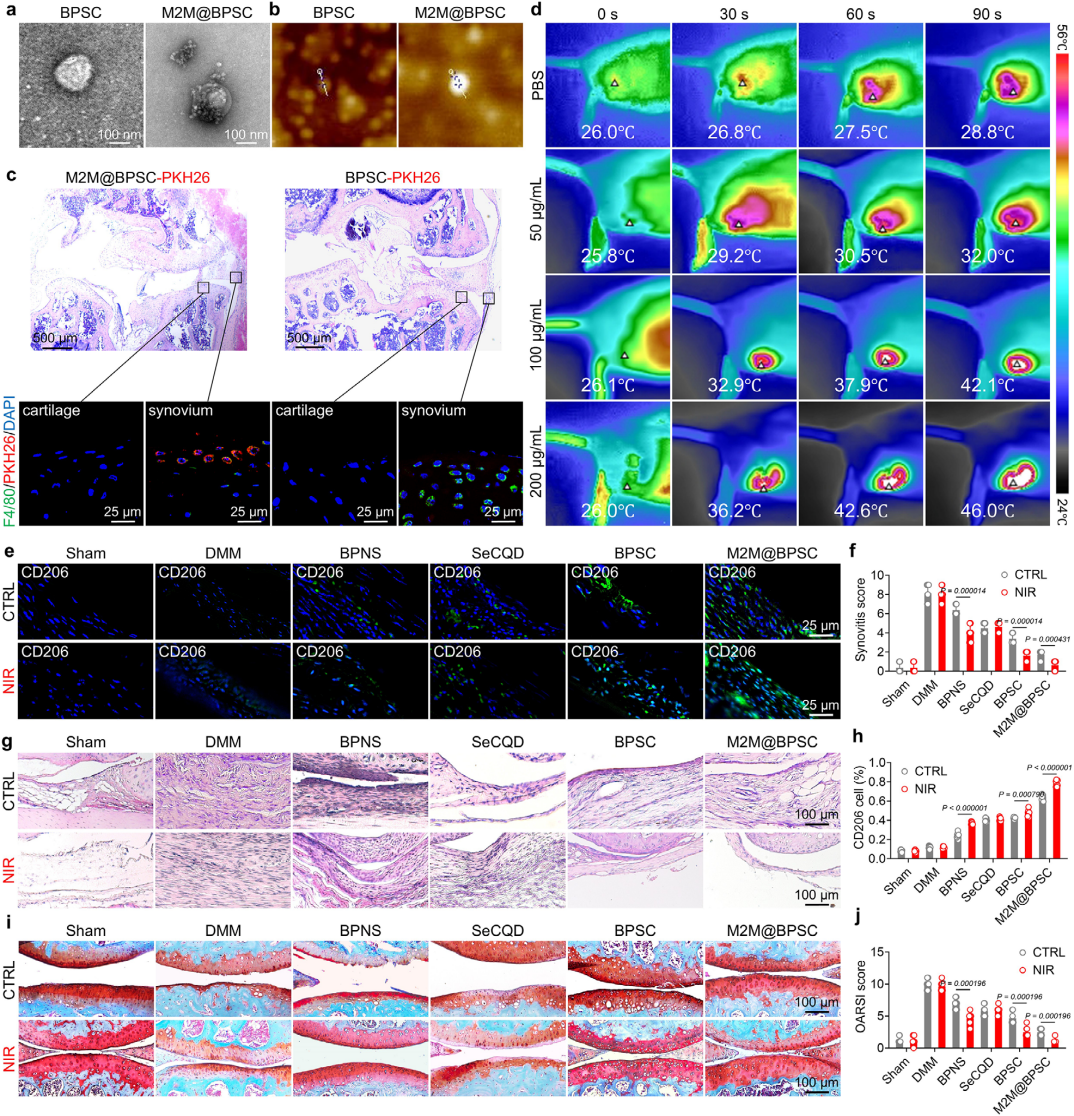
Photothermal amelioration of PTOA pathologies and motor dysfunction by M2M@BPSC. (a) Negative‐stained TEM image of BPSC and M2M@BPSC. (b) AFM image of BPSC and M2M@BPSC. (c) H&E staining and immunofluorescence staining of F4/80 (green), PKH26 (red), and DAPI (blue) in the cartilage and synovium of mice 14 days after DMM surgery with injection of pkh26/M2M@BPSC. (d) Real‐time thermal imaging and quantitative analysis of mice injected with M2M@BPSC under near‐infrared heat treatment (NIR: 808 nm, 1.25 W/cm^2^). (e,h) Immunofluorescence staining of CD206 (green) and DAPI (blue) in the synovium of mice under control treatment and near‐infrared heat treatment (NIR: 808 nm, 1.25 W/cm^2^). (f,g) H&E staining and quantitative analysis of synovium in mice under control treatment and near‐infrared heat treatment (NIR: 808 nm, 1.25 W/cm^2^) (n = 8). (i,j) Safranin‐O (S.O.) staining and quantitative analysis of knee joint sections in mice under control treatment and near‐infrared heat treatment (NIR: 808 nm, 1.25 W/cm^2^) (n = 8).

Based on the aforementioned cell experiments, we conducted in vivo studies to assess the therapeutic potential of BPSC‐mediated photothermal effects in alleviating OA. To address the limited targeting capability of BPSC following injection, BPSC was coated with IL‐4‐induced M2 MM using a membrane separation technique [60]. TEM analysis confirmed the presence of a membrane structure on the surface of the nanosheets in M2M@BPSC (Figure 6a). Furthermore, the diameter (Figure S33) and thickness (Figure 6b; Figure S34) of M2M@BPSC were markedly higher after membrane coating. Owing to its homotypic targeting capability, M2M@BPSC accumulated more in F4/80‐labeled synovial macrophages than BPSC, demonstrating its excellent macrophage‐targeting properties (Figure 6c). In vivo photothermal experiments demonstrated that the local temperature at the knee joints could exceed 42°C after localized NIR irradiation, indicating effective thermal conversion and accumulation of the nanomaterials at the target site (Figure 6d). Subsequently, in vivo distribution studies of the nanosheets using IVIS demonstrated approximately one week of retention within the articular cavity, consistent with the administration protocol of weekly intra‐articular injections (Figure S35). Based on these findings, we established a classical post‐traumatic OA (PTOA) model through surgical destabilization of the medial meniscus and applied treatments involving various nanostructures, with or without NIR stimulation. Owing to the intrinsic anti‐inflammatory properties of induced M2 macrophages, M2M@BPSC demonstrated superior efficacy in reducing the number of M1‐positive macrophages and increasing the number of M2‐positive macrophages (Figure 6e,f). Hematoxylin‐eosin staining demonstrated that fibrosis and hyperplasia in the synovial membrane were alleviated following treatment with BPSC or M2M@BPSC, particularly under NIR laser irradiation (Figure 6g,h). M2M@BPSC+NIR effectively prevented articular cartilage degradation, as indicated by the improved OARSI scores and increased hyaline cartilage thickness (Figure 6i,j; Figure S36). The disruption of C‐ECM was also ameliorated by M2M@BPSC+NIR treatment, as evidenced by the restored anabolic and catabolic metabolism (Figure S37). Gait analysis demonstrated a significant improvement in limb mobility following M2M@BPSC+NIR treatment (Figure S38), and enhanced motor function was further supported by the results of the open‐field test (Figure S39). Importantly, no pathological changes were observed in the major organs, including the heart, liver, spleen, lungs, and kidneys (Figure S40). In vivo tracing experiments have demonstrated that nanoparticles are primarily metabolized by the liver and the kidney (Figure S41). In addition, serum biochemistry analysis indicated that M2M@BPSC did not induce hepatotoxicity or nephrotoxicity in mice (Figure S42). In summary, M2M@BPSC+NIR combined with NIR irradiation effectively attenuated synovial hyperplasia and cartilage destruction, while also contributing to the restoration of joint mobility.

### 2.7 M2M@BPSC+NIR Restored Cartilage and Synovium Homeostasis in a TRPV4‐Dependent Manner

**FIGURE 7:**
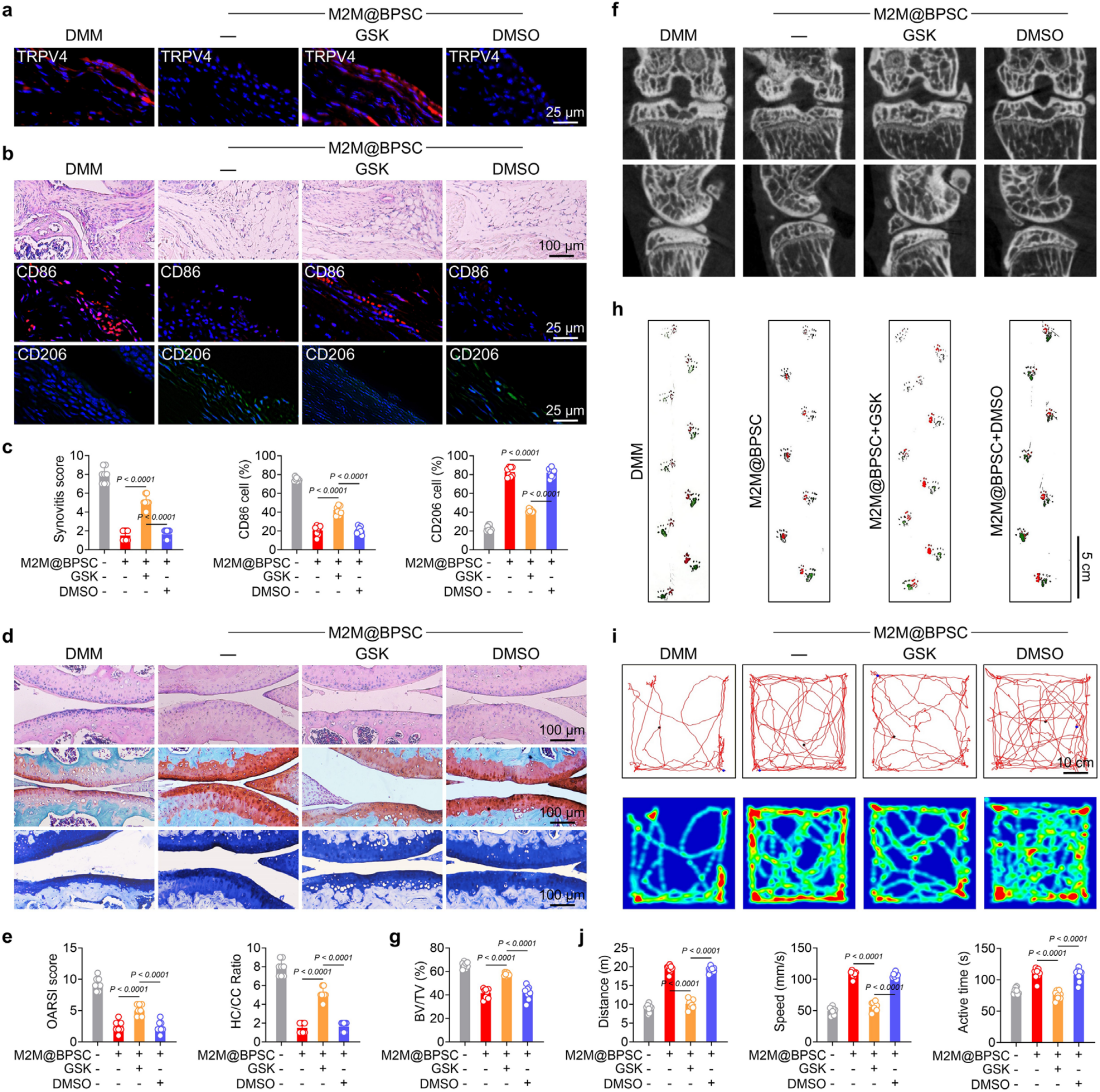
Photothermal remodeling of joint homeostasis via TRPV4 by M2M@BPSC. (a) Immunofluorescence staining of TRPV4 (green) and DAPI (blue) in the synovium of mice under different conditions with near‐infrared heat treatment (NIR: 808 nm, 1.25 W/cm^2^). (b,c) H&E staining, immunofluorescence staining, and quantitative analysis of CD86 (red), CD206 (green), and DAPI (blue) in the synovium of mice under different conditions with near‐infrared heat treatment (NIR: 808 nm, 1.25 W/cm^2^) (n = 8). (d,e) H&E, Safranin‐O (S.O.), and Toluidine Blue (T.B.) staining and quantitative analysis of cartilage in mice under different conditions with near‐infrared heat treatment (NIR: 808 nm, 1.25 W/cm^2^) (n = 8). (f,g) CT reconstruction images and quantitative analysis of mice under different conditions with near‐infrared heat treatment (NIR: 808 nm, 1.25 W/cm^2^) (n = 8). (i,j) Representative open field test plots and quantitative analysis of mice under different conditions with near‐infrared heat treatment (NIR: 808 nm, 1.25 W/cm^2^) (n = 8).

Subsequently, we examined the role of TRPV4 in the therapeutic effects induced by M2M@BPSC+NIR using GSK1016790A, a TRPV4 agonist, to reactivate the channel. Immunofluorescence analysis demonstrated that the administration of M2M@BPSC combined with NIR treatment suppressed TRPV4 expression in synovial tissue, whereas this suppression was reversed by treatment with GSK1016790A (Figure 7a). Furthermore, GSK1016790A attenuated the therapeutic effects on synovial inflammation, as evidenced by persistent FLS hyperplasia and dysregulated polarization within the synovial tissue (Figure 7b,c). Regarding cartilage protection, TRPV4 activation led to reduced hyaline cartilage thickness and promoted cartilage wear and degradation (Figure 7d,e). The metabolic balance of C‐ECM in the articular cartilage was also disrupted following treatment with GSK1016790A (Figure S43). Meanwhile, the TRPV4 agonist exacerbated subchondral bone sclerosis and osteophyte formation, contributing to pathological bone remodeling (Figure 7f,g). Functional assays demonstrated that M2M@BPSC+NIR promoted recovery of lower limb mobility by inhibiting the TRPV4 signaling pathway, as revealed by gait analysis (Figure 7h). Open‐field experiments revealed that PTT treatment increased behavioral activity in PTOA mice, an effect attenuated by GSK1016790A (Figure 7i,j). Overall, M2M@BPSC+NIR effectively restored the homeostasis of the cartilage, subchondral bone, and synovium through TRPV4‐dependent mechanisms.

## 3 Conclusions

Given the limitations of conventional clinical hyperthermia in alleviating OA, this study proposes a novel photothermal converter, M2M@BPSC, designed to achieve precise and mild heating effects on synovial macrophages. The localized temperature increase triggers closure of the TRPV4 ion channel, thereby restricting calcium influx and preventing mitochondrial calcium overload. Furthermore, TRPV4 inhibition activates the STAT6‐EGR2 axis, promoting the polarization of macrophages toward an anti‐inflammatory phenotype. Ultimately, treatment with M2M@BPSC restored the balance between F‐ECM and C‐ECM, suppressing synovial inflammation and mitigating cartilage degeneration. These findings highlight M2M@BPSC as a promising strategy for combined photothermal‐immunomodulatory therapy for OA.

## Conflicts of Interest

The authors declare no conflicts of interest.

## Supporting information




**Supporting file**: advs74714‐sup‐0001‐SuppMat.docx.

**Supplemental Video**: advs74714‐sup‐0002‐VideoS1.mp4

## Data Availability

The data that support the findings of this study are available from the corresponding author upon reasonable request.
