# Supplementary material for: A Photothermal Transducer Regulates Transmembrane Calcium Flow for Synergistic Osteoarthritis Therapy
Source: Adv Sci (Weinh). 2026 Mar 6;13(28):e20157. doi: 10.1002/advs.202520157 (PMC13185896; doi:10.1002/advs.202520157)
Supplement: Supplementary file 1 — Supporting file: advs74714‐sup‐0001‐SuppMat.docx. [file ADVS-13-e20157-s002.docx]

**Materials and Methods**

**Synthesis of BPNS**

Black phosphorus nanosheets (BPNS) were synthesized using a modified liquid exfoliation technique. Briefly, 5 mg of bulk BP was dispersed in 20 mL of deionized water, followed by argon bubbling to eliminate dissolved oxygen. The mixture underwent probe ultrasonication in an ice-water bath for 8 hours (power: 700 W, on/off cycle: 2 s/4 s). Afterward, the dispersion was centrifuged at 3000 rpm for 20 minutes to remove unexfoliated BP. The brown supernatant containing exfoliated BP nanosheets was collected and stored at 4°C for future use. Prior to use, the supernatant was centrifuged at 10,000 rpm for 10 minutes to remove excess solvent.

**Synthesis of BPSC**

Se-carbon quantum dots (Se-CQDs, referred to as SC) were synthesized in situ on the surface of BPNS *via* a chemical reduction method. Specifically, 5 mL of the BPNS dispersion was mixed with 40 mL of deionized water and 5 mL of L-selenocystine solution (15 mg/mL). The pH was adjusted to 10.0 using 0.1 M NaOH, and the mixture was heated to 60°C for 20 hours under continuous nitrogen purging. The solid product was collected by centrifugation at 12,000 rpm for 15 minutes, followed by washing with deionized water (3 ×) to remove unreacted precursors.

**Preparation of M2M@ BPSC**

M2 bone marrow-derived macrophages (BMDMs) were polarized with 20 ng/mL IL-4 in DMEM containing 10% FBS for 72 hours. Cells were lysed using ice-cold buffer (50 mM Tris-HCl, pH 7.4, 150 mM NaCl, 1 mM PMSF [Sigma-Aldrich], 1×protease inhibitor cocktail). After a 2-hour incubation at 4°C, the suspension underwent three freeze-thaw cycles: freezing in liquid nitrogen (10 minutes) and thawing in a 37°C water bath (≤5 minutes). The lysate was centrifuged at 500×g for 10 minutes at 4°C to remove debris. The supernatant was further centrifuged at 12,000×g for 15 minutes at 4°C. The resulting pellet was resuspended in TM buffer. M2 membranes and BPSC were dispersed *via* bath sonication for 15 minutes. The mixture was then extruded through 400-nm and 200-nm polycarbonate membranes (15 passes) using a mini-extruder (Avanti, USA), yielding M2M@BPSC.

**Characterization**

Morphological characterization of BPNS and BPSC was conducted using transmission electron microscopy (TEM, Hitachi, Japan), scanning electron microscopy (SEM, Zeiss GeminiSEM 300, Germany), and atomic force microscopy (AFM, Bruker, Billerica, MA, USA). The morphology of M2M@BPSC was evaluated by TEM and AFM. Composition and structural analysis were performed using UV–visible spectrophotometry (TU-1900, Beijing Purkinje General, China), X-ray photoelectron spectroscopy (XPS, Thermo Scientific K-Alpha, USA), high-resolution TEM (HR-TEM, Tecnai G2 F20 U-TWIN, Japan) with energy-dispersive X-ray spectroscopy (EDS), and Fourier-transform infrared spectroscopy (FTIR, Thermo Fisher Nicolet 6700). Hydrodynamic particle sizes were determined by dynamic light scattering (DLS, Malvern Zetasizer Nano ZS, Germany).

**Radical Scavenging Activity Quantification**

Free-radical scavenging activity was assessed using the DPPH (ml092650, MLBIO), ABTS (ml092653, MLBIO), and •OH-scavenging (ml076360, MLBIO) assays. In the DPPH assay, a 100 μM DPPH solution was prepared, and nanocomposite samples were dispersed in 2.0 mL of ethanol and mixed with the DPPH solution. PBS was used as the blank. After a 30-minute dark incubation at 37°C, absorbance was measured at 517 nm. For the ABTS assay, 100 μL of the nanocomposite solution was combined with 100 μL of a 100 μM ABTS solution, vortexed, and absorbance was recorded at 413 nm. For the •OH scavenging assay, 100 mg of the sample was mixed with 500 μL of 1 mM FeSO₄ and 500 μL of 100 mM H_2_O_2_, incubated at 37°C for 1 hour, and cooled to 25°C. The supernatant (100 μL) was then mixed with 100 μL of 10 mM TMB (in DMSO), and absorbance was measured on a TU-1900 spectrophotometer according to the manufacturer’s protocol. Activity spectra were generated using Origin 2021 software.

**Photothermal properties assessment**

Photothermal performance and local temperature regulation during near-infrared (NIR) irradiation were monitored using an infrared thermal imaging system (Model 616C, FOTRIC, China), enabling real-time, noninvasive assessment of temperature changes at the irradiation site. For *in vitro* characterization, nanocomposite suspensions were dispersed in PBS and exposed to 808 nm NIR laser irradiation at power densities of 0.75, 1.25, and 1.75 W cm⁻², with infrared thermograms acquired at 30 s intervals and temperature values extracted from a predefined region of interest corresponding to the laser spot. For *in vivo* experiments, mice were anesthetized with 2% isoflurane and positioned on a temperature-controlled platform to minimize systemic thermal fluctuations, followed by intra-articular injection of the nanocomposites (20 μL, 200 μg mL⁻¹ in PBS). The knee joint was then irradiated with an 808 nm laser at a power density of 1.25 W cm⁻², with the laser-to-joint distance fixed at 20 cm and the laser spot diameter standardized to 0.5 cm to ensure consistent irradiation geometry. To achieve precise mild hyperthermia while preventing overheating, an intermittent irradiation protocol was applied, consisting of 1.5 min of laser exposure followed by a 1 min pause, repeated for three consecutive cycles. Throughout the entire irradiation procedure, continuous real-time infrared thermal imaging was performed with the camera aligned perpendicular to the joint surface, and laser output was dynamically adjusted or immediately terminated once the local temperature approached or exceeded the predefined therapeutic window of 41.5 to 42.5℃ (Supplementary Video). No visible skin burns, local edema, or abnormal behavioral responses were observed during or after irradiation, confirming the local thermal safety and reproducibility of the optimized photothermal protocol.

**Cell culture**

BMDMs were isolated from 8-week-old male C57BL/6J mice. Following euthanasia, the mice were sterilized with 75% ethanol and transferred to sterile glass plates. The femora and tibiae were aseptically dissected, stripped of soft tissue, and placed in ice-cold PBS. Epiphyses were resected to expose the marrow cavities, which were flushed repeatedly with complete medium using a 20-gauge needle attached to a 20 mL syringe. The cell suspension was centrifuged (500×g, 5 minutes, 4°C), and erythrocytes were lysed with ACK buffer. After a second centrifugation, the nucleated cells were resuspended in DMEM supplemented with 10% FBS (F103, Vazyme Biotech, China) and 30 ng/mL recombinant murine M-CSF (CB34, Novoprotein, China) before being seeded in 10-cm Petri dishes. Non-adherent cells were removed after 16 hours, and adherent cells were cultured for an additional 3 days to obtain mature BMDMs. Chondrocytes were isolated from the knee joints of 1-week-old C57BL/6J mice. Following euthanasia, the mice were sterilized with 75% ethanol and placed under sterile conditions. The overlying skin was incised with sterile instruments, and the articular cartilage was excised. Cartilage fragments were minced, washed with 10% penicillin–streptomycin solution (Gibco, USA), and then rinsed with sterile PBS (RG-CE-10, KETU, China). Tissue pieces were digested overnight at 37°C in 0.2% Type II collagenase (Sigma-Aldrich, USA). Undigested material was removed by filtration through a 70-µm cell strainer, and the digest was centrifuged (500×g, 5 minutes). The pellet was resuspended and plated into 100-mm dishes (704202, NEST Biotechnology, China) to obtain P0 chondrocytes. Cells were maintained in DMEM/F-12 (Keygen BioTECH, China) with 10% FBS (F103, Vazyme Biotech, China), 100 U/mL penicillin, and 100 µg/mL streptomycin at 37°C and 5% CO_2_. P1 chondrocytes were used for all experiments. Fibroblasts were isolated from mouse embryos. The embryo trunk was minced and digested with 0.25% trypsin-0.02% EDTA at 37°C for 10 minutes, then resuspended in DMEM containing 10% FBS. After counting, the cells were plated at 5×10^5^ cells per dish at 37°C and 5% CO_2_. The medium was changed every 2 days, and cells were passaged every 3–4 days. P3–P5 fibroblasts were used for experiments.

**siRNA-mediated knockdown of TRPV4 and STAT6 in mouse BMDMs**

Bone marrow-derived macrophages (BMDMs) were prepared as described above and seeded in 6-well plates at a density of 100,000 cells per well. Small interfering RNAs (siRNAs) targeting mouse *Trpv4* or *Stat6*, along with a non-targeting scrambled siRNA control, were procured from GenePharma (Shanghai, China). Transfection was carried out using Lipofectamine™ RNAiMAX (Thermo Fisher Scientific) following the manufacturer's guidelines. In brief, siRNA duplexes and Lipofectamine™ RNAiMAX were separately diluted in Opti - MEM, gently mixed, and incubated at room temperature for 10-20 minutes to facilitate complex formation before being added drop-by-drop to the cells. The final working concentration of siRNA was set at 50 nM. After 6 hours of transfection, the medium was replaced with complete culture medium, and the cells were incubated for an additional 24-48 hours before downstream experiments. Subsequently, the siRNA-transfected BMDMs were subjected to macrophage polarization stimulation, nanocomposite treatment, and/or near-infrared (NIR) irradiation as specified. The knockdown efficiency of TRPV4 and STAT6 was verified by quantitative real-time PCR and western blotting prior to functional assays, and only cells with effective knockdown were included in further analyses.

**Induction of macrophage polarization**

BMDMs were stimulated with M-CSF (20 ng/mL) for 24 hours to ensure macrophage survival and proliferation, and then co-stimulated with LPS (100 ng/mL) and IFN-γ (20 ng/mL) to induce classical activation and polarization into the M1 phenotype. The M2 phenotype was induced by co-stimulation with M-CSF (20 ng/mL) and IL-4 (20 ng/mL) for 72 hours to promote alternative activation. Appropriately polarized macrophages were used for downstream experiments.

**Whole-cell patch-clamp recording**

Whole-cell voltage-clamp recordings were carried out to evaluate TRPV4-mediated membrane currents in mouse bone marrow-derived macrophages (BMDMs). The experiments were conducted at room temperature (22-24 °C) with the use of an EPC-10 amplifier (HEKA Elektronik, Germany) controlled by PatchMaster software. Differentiated BMDMs were plated on glass coverslips and permitted to adhere before recording. Patch pipettes were fabricated from borosilicate glass capillaries (BF150-86-10, Sutter Instrument) and had a resistance ranging from 2 to 4 MΩ. Cells were continuously perfused through a gravity-driven local perfusion system (flow rate ≥100 μL·min⁻¹) to guarantee rapid and stable control of the extracellular environment. The extracellular solution was composed of 140 mM NaCl, 5 mM KCl, 2 mM CaCl₂, 1 mM MgCl₂, 10 mM glucose, and 10 mM HEPES (pH 7.4; osmolarity approximately 340 mOsm/L). For current-voltage analysis, membrane currents were evoked by a voltage ramp protocol from -100 mV to +100 mV over a period of 500 ms starting from a holding potential of 0 mV. The signals were low-pass filtered at 2 kHz and digitized at 10 kHz, and the I–V relationships were analyzed using Clampfit 10. To assess the time-dependent modulation of TRPV4 activity in the same cell, recordings were performed at a holding potential of -60 mV, with currents sampled at 5 kHz and low-pass filtered at 1 kHz. Single BMDM was incubated with BPSC (100 μg/mL) for 12 h prior to the recording. During continuous recording, NIR irradiation (808 nm, 1.25 W/cm²) was applied, and the TRPV4 agonist GSK1016790A was administered at specified time points (0, 30, 60, and 90 s). Current amplitudes were normalized to the same-cell baseline recorded at 0 s (Iₜ/I₀) to reduce intercellular variability.

**Co-culture assay​​**

M1 BMDMs were generated by stimulating primary BMDMs with lipopolysaccharide (LPS, 100 ng/mL) and interferon-γ (IFN-γ, 20 ng/mL) for 24 hours in complete DMEM medium supplemented with 10% fetal bovine serum (FBS). Conditioned medium (M1-CM) was collected from each experimental group, centrifuged at 1,000×g for 5 minutes at 4°C to remove cellular debris, and diluted 1:1 (v/v) with serum-free DMEM/F-12 medium. The resulting M1-CM was applied to two distinct cell models: IL-1β-stimulated mouse chondrocytes (10 ng/mL IL-1β pretreatment for 24 hours) to assess chondrocyte extracellular matrix (ECM) degradation *via* collagen II loss, and TGF-β-stimulated fibroblasts (5 ng/mL TGF-β pretreatment for 48 hours) to measure fibroblast proliferation using CCK-8 assays. All treatments were maintained under standard culture conditions (37°C, 5% CO_2_).

**Cellular intervention**

Cells were plated at 5,000 cells/cm^2^ and maintained at 37°C/5% CO_2_. To model arthritis, cells were treated with LPS (100 ng/mL) and IFN-γ (20 ng/mL) for 24 hours. Nanocomposites were sterilized by three washes with 75% ethanol (v/v), followed by three PBS rinses. Nanocomposite-free controls received the same 24-hour treatment. *In vitro* experimental groups included: Experiment 1: Ctrl, LPS, LPS+BPNS, LPS+SeCQD, LPS+BPSC; Experiment 2: LPS, LPS+BPSC, LPS+BPSC+GSK1016790A (10 nM), LPS+BPSC+DMSO. After 24 hours of dark incubation, cells were subjected to NIR irradiation (808 nm, 1.25 W/cm^2^, 90 seconds) or left untreated.

**Micromass culture for chondrogenic assessment**

Micromass culture was employed to quantify cartilage-specific ECM deposition. Primary mouse chondrocytes were suspended at 2.0×10⁷ cells/mL in DMEM/F-12 medium supplemented with 10% FBS and 1% penicillin-streptomycin. A 50 μL droplet of cell suspension was dispensed centrally into each well of a 24-well plate. After 2 hours of adhesion at 37°C/5% CO_2_, 1 mL of conditioned medium containing experimental treatments was gently added. Cultures were maintained for 72 hours, with complete medium replacement and treatment renewal every 24 hours. After culture, micromasses were fixed in 4% paraformaldehyde (4°C, 24 hours) and stained with 0.2% alcian blue (pH 0.2) or Safranin O for sulfated glycosaminoglycan visualization. ECM deposition was quantified using ImageJ.

***In vitro* assays for cell migration and invasion**

Cell migration was assessed using 24-well Transwell chambers (Corning). The upper chamber received 5×10^4^ cells in 100 μL serum-free medium, and the lower chamber contained 600 μL medium with 10% FBS as a chemoattractant. After 24 hours of incubation, migrated cells were methanol-fixed (10 minutes) and stained with 0.5% crystal violet. Non-migrated cells were removed from the upper chamber. Migrated cells were quantified by counting three random microscope fields. For invasion assays, membranes were pre-coated with Matrigel (30 μg, Corning); otherwise, the procedures were identical to the migration assays.

**Wound-healing assay**

Cells were seeded at a density of 5×10^5^ cells per well in 6-well plates and cultured until a confluent monolayer was formed. A standardized linear wound was then introduced by gently scraping the cell sheet with a sterile 200 μL pipette tip held perpendicular to the plate surface, ensuring consistent pressure and angle across all wells to minimize variability. Following the injury, each well was carefully washed twice with warm PBS to remove detached cells and debris, after which the medium was replaced with an equal volume of serum-free medium to suppress proliferation and focus the assay on directed cell migration. Wound closure was monitored and digitally photographed at the same positions at 0, 12, and 24 hours using an inverted microscope equipped with a phase-contrast objective and a fixed camera setting to ensure reproducible image acquisition.

**Cytocompatibility investigation**

BMDMs were plated in 96-well plates at 5,000 cells per well and maintained at 37°C/5% CO_2_. At the indicated time points, cells were treated with vehicle, LPS (100 ng/mL), BPNS, SeCQD, or BPSC (100 µg/mL). Cell viability was assessed using the CCK-8 assay according to the manufacturer’s instructions. After 6, 12, or 24 hours of treatment, the medium was replaced with 100 µL of fresh medium containing 10 µL of CCK-8 reagent. After 2 hours of incubation, 100 µL of stop solution was added to each well, and plates were protected from light at 25°C for 4 hours. Absorbance was measured at 570 nm using a Tecan microplate reader (BioTek Instruments Inc.), and viability was calculated relative to controls.

**Live/Dead cell staining assay**

BMDMs were cultured in 24-well plates and treated with LPS (100 ng/mL), BPN, SeCQD, or BPSC as specified. After 24 hours, cells were stained with calcein-AM and propidium iodide (PI) (Thermo Fisher Scientific, USA) for 20 minutes at 37°C to assess viability. Live cells showed green fluorescence from cleaved calcein-AM, while dead cells displayed red fluorescence from PI. Fluorescence images were acquired using an inverted microscope (Axio Observer 7, Zeiss, Germany) with a 20×objective.

**Flow cytometric analysis**

BMDMs were seeded at a density of 2×10^4^ cells/mL in direct co-culture with BPNS, selenium-carbon quantum dots (SeCQDs), or black phosphorus-selenium nanocomposites (BPSC). After 24-hour stimulation with LPS (100 ng/mL) and IFN-γ (20 ng/mL) in complete medium supplemented with 10% FBS, cells were harvested by gentle scraping. Single-cell suspensions were prepared by sequential filtration through 70-μm and 40-μm cell strainers (Falcon, USA). Cell surface markers were stained for 15 minutes at 25°C in the dark using the following fluorochrome-conjugated monoclonal antibodies (all from eBioscience, USA) at the recommended concentrations: Anti-F4/80-phycoerythrin (PE), Anti-CD86-phycoerythrin-cyanine 7 (PE-Cy7), and Anti-CD206-fluorescein isothiocyanate (FITC) (Clone C068C2, Cat# 11-2061-82). After two washes with ice-cold phosphate-buffered saline (PBS, pH 7.4), stained cells were resuspended in 300 μL PBS. Surface expression was quantified using a Guava® easyCyte 12HT flow cytometer (Merck Millipore, Germany). Data analysis was performed using FlowJo v10.8.1 software (BD Life Sciences, USA).

**Mitochondrial function detection**

Mitochondrial membrane potential (MMP) was evaluated using the JC-1 MMP Assay Kit (Beyotime, China). BMDMs were incubated with JC-1 working solution (0.5 µM) at 37°C for 30 minutes, protected from light, then washed with cold JC-1 staining buffer. Fluorescence images were captured using fluorescence microscopy. MMP was quantified by calculating the red/green fluorescence ratio (JC-1 aggregates/monomers) using ImageJ software.

Intracellular and mitochondrial reactive oxygen species (ROS) levels were measured using DCFH-DA (MedChemExpress, USA) and MitoSox (MedChemExpress, USA) probes. Cells were incubated at 37°C with a working solution containing 5×10^–6^ M DCFH-DA or 5×10^–3^ M MitoSox for 30 minutes. ROS detection was performed using a standard fluorescence microscope. Quantitative analysis was conducted using ImageJ software.

**Seahorse assay**

Mitochondrial oxygen consumption rate (OCR) was measured using the Seahorse Extracellular Flux XFe24 analyzer (Agilent, USA). BMDMs from each experimental group were seeded at 5×10^4^ cells/well in Seahorse XF-24 culture plates. Prior to analysis, cell plates were incubated in a CO_2_-free incubator for 1 hour. For OCR analysis, Oligomycin (1×10^–6^ M), FCCP (1.5×10^–6^ M), and rotenone/antimycin (R/A) (2×10^–6^ M) were sequentially injected. Basal respiration was determined as the last OCR before Oligomycin injection minus non-mitochondrial respiration; maximum respiration was assessed as the highest OCR after FCCP injection minus the lowest OCR after R/A injection; ATP production was evaluated as the OCR before Oligomycin injection minus the lowest OCR after Oligomycin injection.

**Calcium imaging detection**

Cytosolic and mitochondrial calcium ion (Ca^2+^) influx was quantified in BMDMs by loading cells with 5 μM Fluo-3 AM (HY-D0716, MedChemExpress) for cytosolic Ca^2+^ detection and 5 μM Rhod-2 AM (HY-D0989, MedChemExpress) for mitochondrial Ca^2+^ detection, followed by a 30-minute dark incubation at 37°C. Fluorescence imaging was performed under standardized microscopy conditions, using 488 nm excitation/525 nm emission for Fluo-3 AM and 552 nm excitation/581 nm emission for Rhod-2 AM.

**Western blot assay**

Cellular proteins were extracted from treated samples using RIPA lysis buffer (Beyotime, China), and supernatants were collected after ultracentrifugation for protein concentration measurement *via* a BCA kit (Beyotime, China). Protein samples were mixed with loading buffer (Beyotime, China) at a 4:1 ratio and denatured at 100°C for 5 minutes. Electrophoresis was performed on 4–12% SDS-PAGE gels (GenScript, M00938, China), followed by protein transfer onto nitrocellulose membranes. Membranes were blocked in blocking buffer (Beyotime, China) for 30 minutes at 25°C, then incubated overnight at 4°C with primary antibodies (1:2000 dilution): anti-CD86 (A21198, Abclonal, China); anti-Mannose Receptor (A8301, Abclonal, China); anti-TRPV4 (A22657, Abclonal, China); anti-EGR2 (A3219, Abclonal, China); anti-p-STAT6 (AP0456, Abclonal, China); anti-STAT6 (A19120, Abclonal, China); anti-p-CAMKII (AP1386, Abclonal, China); anti-COL2 (ab188570, Abcam, USA); anti-MMP13 (ab39012, Abcam, USA); anti-ATP5A (A11217, Abclonal, China); anti-MT-ND4 (A17970, Abclonal, China); anti-SDHA (A2594, Abclonal, China); anti-Collagen I (A21059, Abclonal, China); anti-Collagen III alpha 1 (A0817, Abclonal, China); anti-α-SMA (A17910, Abclonal, China); anti-H3 (A2348, Abclonal, China); β-Actin (GB11001-100, Servicebio, China). After primary antibody removal, membranes were incubated with HRP-conjugated secondary antibodies (1:10,000, Affinity, China) for 1 hour at 25°C. After three 5-minute washes, protein detection was performed using ultrasensitive ECL substrate (NCM Biotech, China) and imaging with a VersaDoc™ system (Bio-Rad, USA). Band intensities were quantified using ImageJ software.

**Quantitative real-time PCR analysis**

Cells in six-well plates were treated as indicated, followed by total RNA extraction using TRIzol reagent (Sigma-Aldrich, USA). RNA concentration and purity were measured with a NanoDrop ND-2000 spectrophotometer (Thermo Fisher Scientific, USA). cDNA was synthesized using a reverse-transcription kit (Thermo Fisher Scientific, USA). The cDNA was mixed with SYBR Green PCR Master Mix, gene-specific primers, and DEPC-treated water to prepare the reaction mixtures. Real-time PCR was performed on a CFX96 system (Bio-Rad, USA) with Gapdh as the endogenous control. Target gene expression was calculated using the 2^⁻ΔΔCt^ method.

**Chromatin immunoprecipitation (ChIP) assay**

ChIP was conducted using a commercial kit (JKR23002A, GENECREATE, Wuhan, China) following the manufacturer's protocol. Briefly, macrophages were cross-linked with 1% formaldehyde at room temperature for 10 minutes, quenched with 0.125 M glycine solution for 5 minutes, and washed twice with ice-cold PBS containing protease and phosphatase inhibitors. Nuclei were lysed on ice using SDS lysis buffer (1% SDS, 10 mM EDTA, 50 mM Tris-HCl pH 8.1, inhibitors) for 10 minutes. Chromatin was fragmented into 200–500 bp fragments by ultrasonic disruption (30 seconds on/30 seconds off, 12 cycles, 4°C). After ribonuclease and proteinase K treatment, fragment sizes were verified by 1.5% agarose gel electrophoresis. The sheared chromatin (50 μg per reaction) was diluted 10-fold with ChIP dilution buffer, pre-cleared with protein A/G magnetic beads at 4°C for 1 hour, and then incubated overnight at 4°C with 3 μg of anti-STAT6 antibody or matching rabbit IgG (negative control). Immunocomplexes were captured using fresh protein A/G magnetic beads (30 μL, 2 hours, 4°C) and washed sequentially with low-salt, high-salt, LiCl, and TE buffers. Bound chromatin was eluted with 200 μL elution buffer (1% SDS, 0.1 M NaHCO_3_, 65°C, 15 minutes) and decrosslinked by incubation at 65°C in 0.2 M NaCl for 4 hours. DNA fragments were purified using the kit’s centrifugal columns and quantified by qRT-PCR with SYBR Green and promoter-specific primers (Supplementary Table S2) on a CFX96 system (Bio-Rad), normalized to input DNA (1% of each original lysate).

**Immunofluorescence staining**

After treatment, cells were fixed with 4% paraformaldehyde (Servicebio, China) for 30 minutes at 25°C. Following permeabilization with 0.1% Triton X-100 for 5 minutes, samples were blocked with blocking buffer (Beyotime, China) for 30 minutes. Primary antibodies (1:200 in blocking buffer) were applied overnight at 4°C. After three PBS washes, cells were incubated with Alexa Fluor 488- or 594-conjugated secondary antibodies (1:500, ABclonal, China) for 1 hour at 25°C in the dark. Nuclei were stained with DAPI (1 µg/mL, Sigma-Aldrich) for 5 minutes. Fluorescence images were acquired using a Zeiss Axiovert 40 CFL microscope under light-free conditions, capturing ≥3 random fields per sample.

**Transcriptome sequencing**

BMDMs were treated with LPS (100 ng/mL) and IFN-γ (20 ng/mL) alone or combined with M2M@BPSC microspheres. Total RNA was sent to Wekemo Tech Group Co., Ltd. (China) for RNA sequencing *via* Affymetrix GeneChip microarray (Affymetrix, USA). RNA integrity was confirmed using an Agilent Bioanalyzer 2100 (Agilent Technologies, USA). Differentially expressed genes (DEGs) were identified using the Affymetrix Microarray Suite, with thresholds of |fold change| > 2 and P < 0.05. Functional enrichment analysis was performed through Gene Ontology (GO), Gene Set Enrichment Analysis (GSEA), and Kyoto Encyclopedia of Genes and Genomes (KEGG) annotations.​

**Animal study**

Osteoarthritis was surgically induced in 8-week-old male C57BL/6 mice by transecting the right knee medial meniscotibial ligament (DMM model), with sham controls receiving identical joint exposure without ligament transection. Postoperative wounds were disinfected with povidone–iodine, and penicillin (50,000 IU/kg) was administered intramuscularly. In Experiment 1 (initiated 1 week post-surgery), mice received weekly intra-articular injections (20 µL) of PBS, BPNS, SeCQD, BPSC, or M2M@BPSC (100 µg/mL) *via* a fine needle under the patella. In Experiment 2, injections (20 µL) contained PBS, M2M@BPSC, M2M@BPSC + GSK1016790A (0.015 µg/µL), or M2M@BPSC + DMSO. Twenty-four hours post-injection, mice received near-infrared (NIR) therapy (808 nm, 1.25 W/cm^2^) or no irradiation. NIR treatment consisted of three 90-second irradiation cycles with 30-second intervals. At 8 weeks post-surgery, mice were euthanized by cervical dislocation under anesthesia, and the heart, liver, spleen, lung, kidney, and knee joints were harvested for histology (Safranin-O/fast green, toluidine blue, H&E staining). All animal experiments were conducted in accordance with the guidelines of the Ethics Committee of Soochow University (SUDA20250320A01).

**M2M@BPSC targeted tracking and major organ biodistribution**

M2M@BPSC was labeled using the lipophilic fluorescent membrane dye PKH26 (SHY-D1451, MedChemExpress) as follows: 1×10^7^ particles were incubated with 5 µM PKH26 in Dilution Buffer at 25°C for 5 minutes, followed by three washes with PBS to remove unbound dye. After labeling, 20 µL (100 µg/mL) of PKH26-M2M@BPSC was immediately injected into the right knee joint of DMM-induced osteoarthritis mice *via* intra-articular injection under sterile conditions. Animals were housed in light-protected cages to minimize photobleaching. Seven days post-injection, the knee joint was carefully dissected under dim red light, fixed overnight in 4% formaldehyde solution at 4°C, decalcified in 10% EDTA solution for 7 days, cryoprotected in 30% sucrose solution, embedded in OCT, and sectioned at 6 µm thickness for subsequent immunofluorescence imaging. Following the intra-articular injection protocol described above, M2M@BPSC was labeled with the near-infrared fluorescent dye Cy5.5 for the purpose of biodistribution and metabolic tracking. Then, it was injected into the knee joints of DMM-induced osteoarthritis mice. Subsequently, major organs were harvested at 7 and 14 days post-injection for ex vivo fluorescence imaging to assess the biodistribution and clearance of the nanomaterials.

***In vivo* imaging systems imaging**

Longitudinal tracking of M2M@BPSC degradation in the mouse joint cavities was performed using an IVIS Spectrum *in vivo* imaging system (Tanon, China). Mice, under isoflurane anesthesia, received intra-articular injections of 20 μL DiR (HY-D1048, MCE)-labeled M2M@BPSC (100 µg/mL). Anesthesia was reapplied for imaging on days 1, 3, 5, and 7 post-injection. During imaging, mice were placed laterally on a heated stage (37°C), with fixed exposure settings. Kinetic curves were derived from sequential images. Circular regions of interest (ROIs) were drawn over the joint cavities, and residual M2M@BPSC was quantified by measuring integrated fluorescence (average radiance, p/s/cm^2^/sr).

**Open field test**

Locomotor and exploratory behaviors were evaluated using an automated computer-vision tracking system (Shanghai Xinruan Information Technology Co., Ltd., Shanghai, China). After 30 minutes of dark adaptation, each mouse was gently placed in the center of a matte-black open-field chamber (50 cm×50 cm×40 cm). Activity trajectories were recorded continuously for 3 minutes at 25 frames per second. The software automatically calculated total movement distance (cm), cumulative active time (s), and average velocity (cm/s). Between trials, the arena was wiped with 70% ethanol and dried to eliminate odor cues, and the camera lens was recalibrated to maintain spatial accuracy (± 1 mm).

**Footprint gait analysis**

Mouse forelimbs and hindlimbs were gently coated with non-toxic, water-based red and green dyes, respectively, taking care to prevent spillage onto fur. Each animal was released at one end of a 70 cm×20 cm runway, with the floor lined with fresh white paper taped flush to the surface. Mice were allowed to traverse the track freely for three consecutive trials, separated by 5-minute rest intervals. Footprints deviating by more than 5 mm from the central 10 cm lane were discarded. Forepaw (red) and hindpaw (green) imprints were captured on the paper and scanned at 600 dpi for automated analysis using ImageJ. To minimize novelty stress, all animals underwent a one-week environmental acclimation period, which included daily 5-minute exploration sessions on a paper-covered runway identical to the test setup.

**Micro-CT**

Knee specimens were harvested from euthanized mice 8 weeks post-surgery and fixed in 4% paraformaldehyde (Servicebio, China) for 24 hours. The specimens were subjected to high-resolution micro-CT scanning (SkyScan 1176, Aartselaar, Belgium) under standardized parameters (60 kV, 170 mA, 0.7° rotation, 18 μm resolution). Scanned data were reconstructed and analyzed using NRecon, Data Viewer, CTAn, and Mimics software for 3D quantitative analysis. Osteoarthritis severity was assessed by calculating bone volume/tissue volume (BV/TV), trabecular separation (Tb.Sp), and trabecular thickness (Tb.Th).

**Immunohistofluorescence staining**

Tissue sections were deparaffinized in xylene and dehydrated through graded ethanol. Endogenous peroxidase activity was blocked with 3% H_2_O_2_ (15 minutes), followed by antigen retrieval with hyaluronidase (2 mg/mL, Sigma-Aldrich, USA) at 37°C. Sections were blocked with 1.5% goat serum and incubated with primary antibodies (1:200) overnight at 4°C. After removal of the primary antibody, sections were incubated with Alexa Fluor 488/594-conjugated secondary antibodies (1:500, Abcam, UK) for 1 hour at 25°C, protected from light, and counterstained with DAPI. Imaging was performed using a Zeiss Axiovert 40CFL microscope, and quantitative analysis was conducted using ImageJ.

***In vivo* toxicity**

Following the final treatment, mice were euthanized under deep isoflurane anesthesia and immediately subjected to gross necropsy. The heart, liver, spleen, lungs, and both kidneys were rapidly excised, rinsed with ice-cold PBS to remove residual blood, and fixed in 10% neutral-buffered formalin at 4°C for 48 hours. After fixation, tissues were dehydrated through a graded ethanol series (70%, 80%, 95%, 100%), cleared with xylene, and embedded in paraffin. Serial 4 µm sections were cut on a rotary microtome, mounted on poly-L-lysine-coated slides, and dried overnight at 37°C. Following deparaffinization and rehydration, sections were stained with Mayer’s hematoxylin for 5 minutes and eosin Y for 2 minutes. After dehydration and coverslipping with synthetic resin, slides were examined under a light microscope for histopathological evaluation, including assessment of inflammation, necrosis, fibrosis, or other structural abnormalities.

**Serum biochemical analysis**

For each mouse, 200 μL of serum was collected and stored at 4 °C before analysis. Subsequently, serum biochemical parameters, including total protein (TP), albumin (ALB), globulin (GLB), alanine aminotransferase (ALT), aspartate aminotransferase (AST), total bilirubin (T-Bil), direct bilirubin (D-Bil), indirect bilirubin (I-Bil), blood urea nitrogen (BUN), and creatinine (CRE), were analyzed using an automated biochemical analyzer (Model 7020, Hitachi, Japan) according to the manufacturer's standard protocols.

**Statistical analysis**

Data are presented as mean ± SD unless otherwise specified. Statistical significance was assessed using one-way ANOVA with multiple comparisons or two-tailed Student's t-test (for two-group comparisons), with *P* < 0.05 considered significant. Analyses were performed using GraphPad Prism.

**Supplementary Figures and Legends**

**
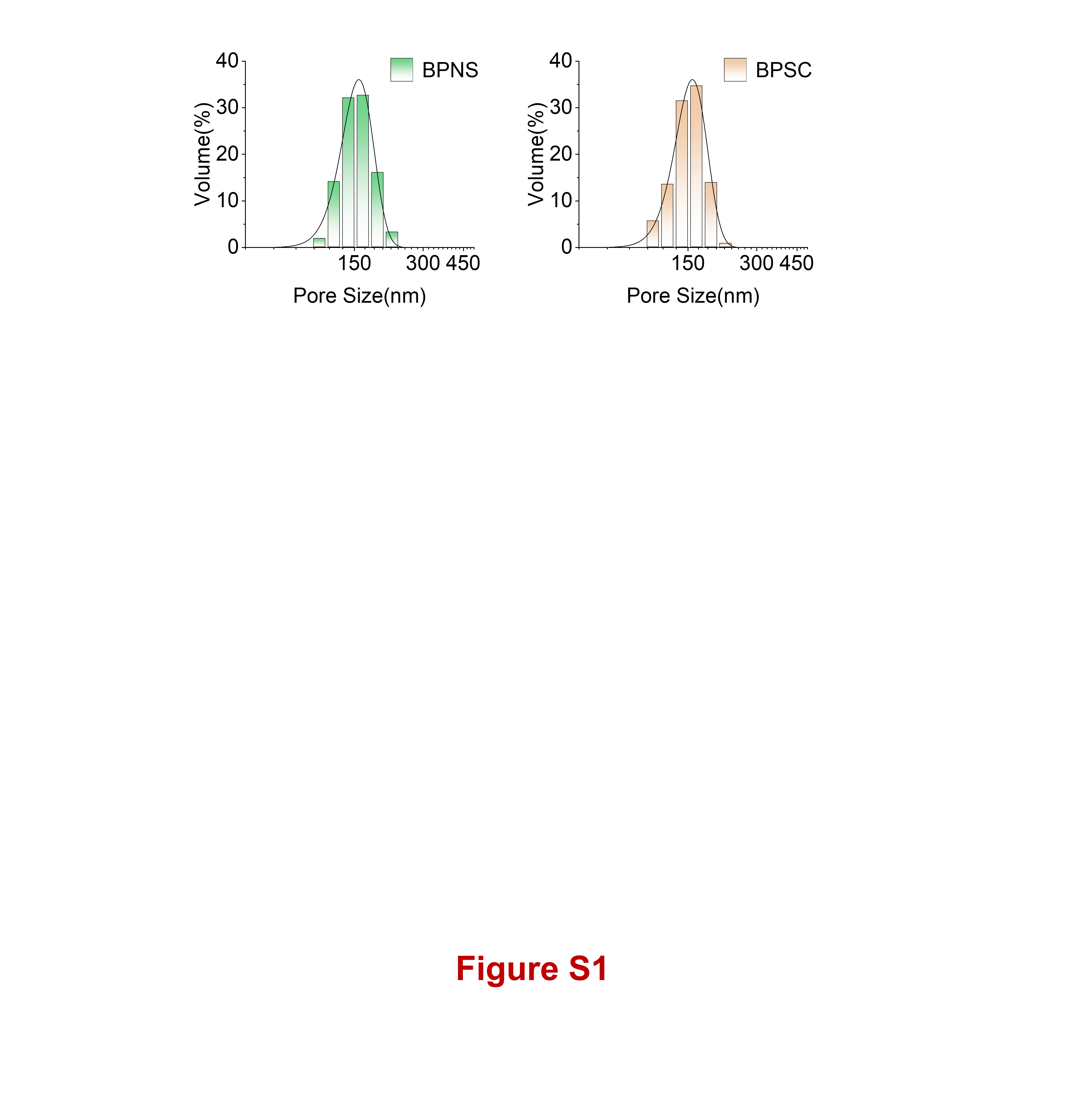
**

**S1:** Size distribution of BPNS and BPSC determined by DLS.


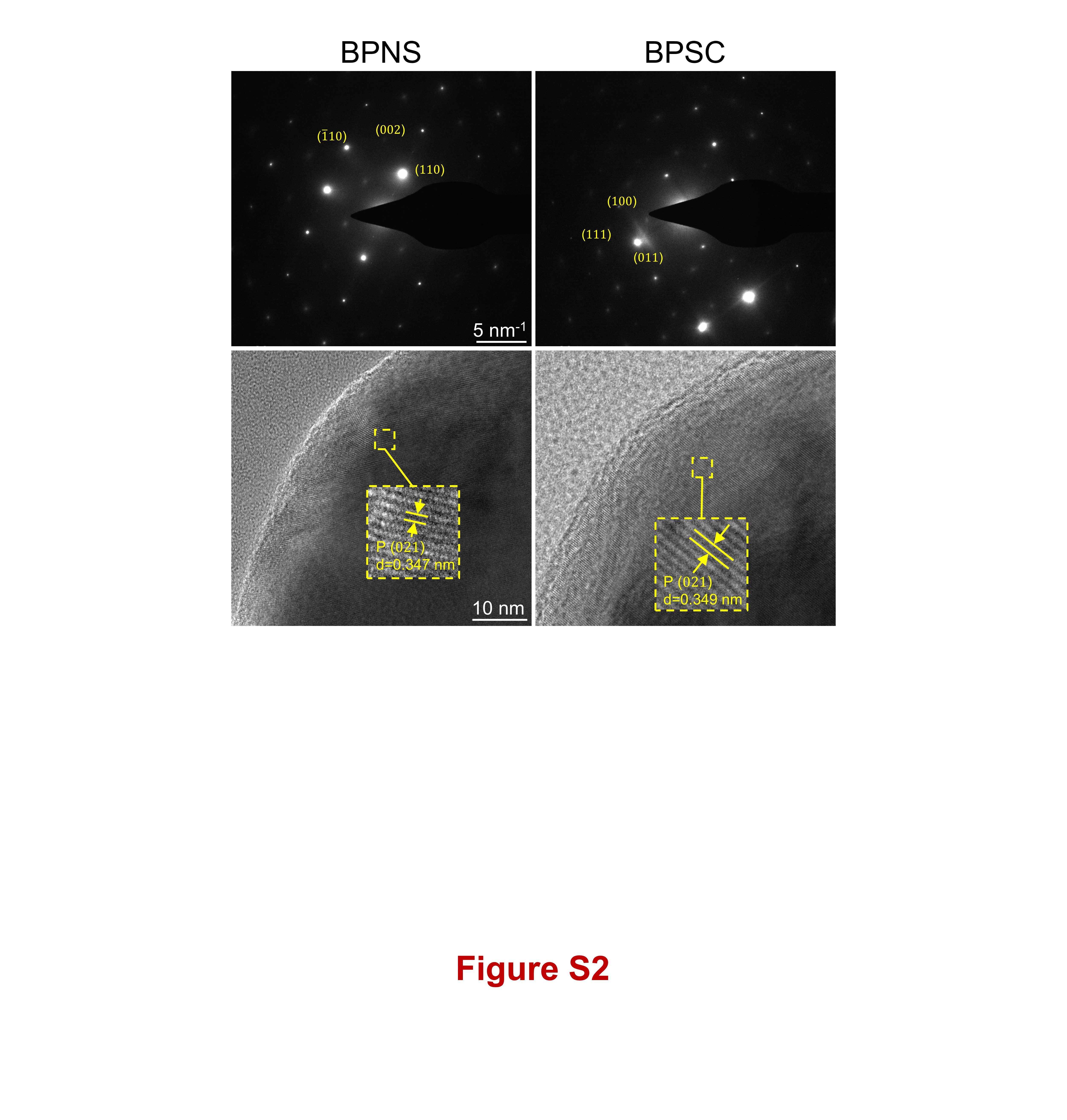


**S2:** SAED pattern and HRTEM image of BPNS and BPSC.

**
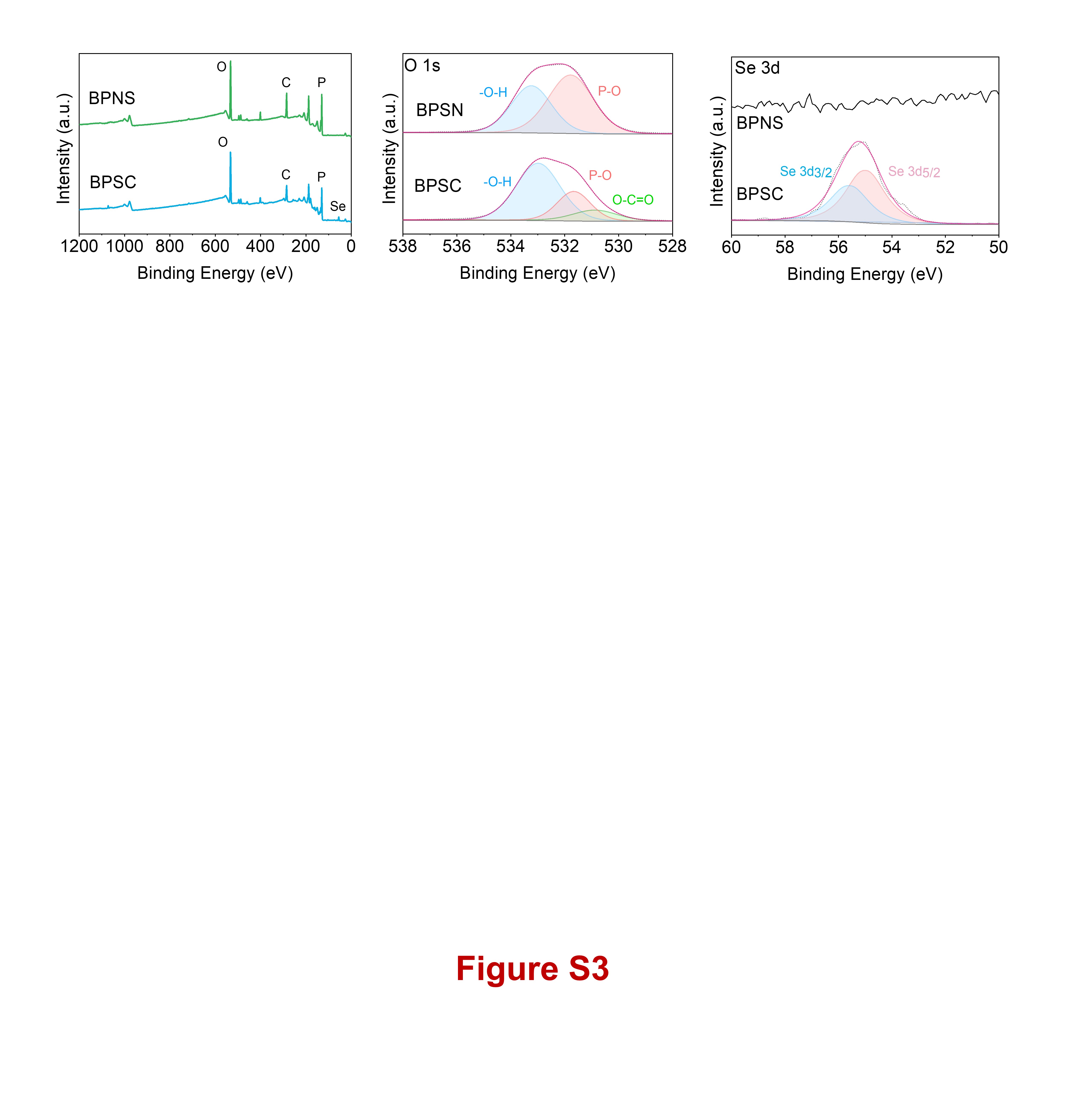
**

**S3:** XPS analysis of BPNS and BPSC.

**
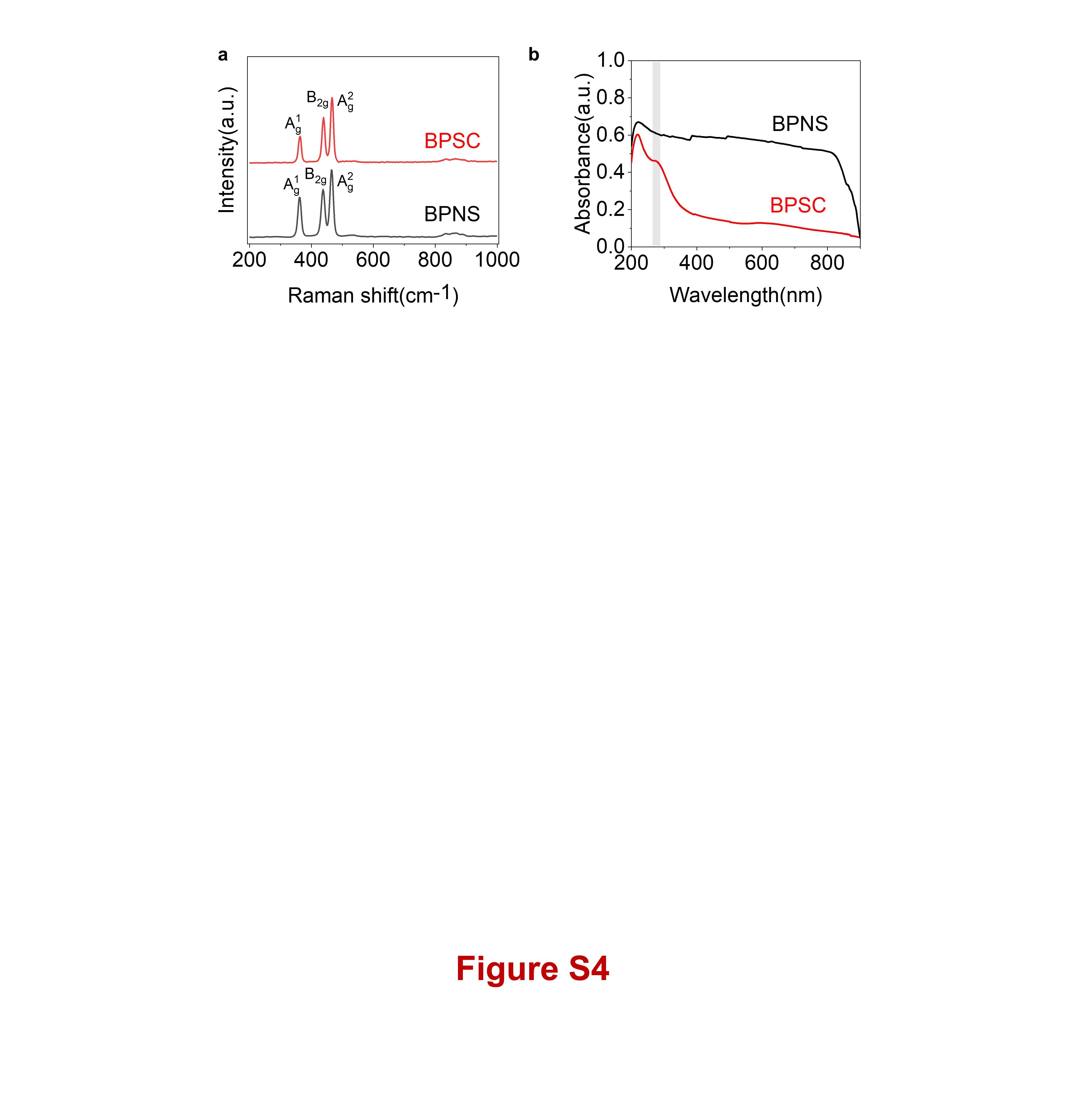
**

**S4:** FTIR and Raman spectra of BPNS and BPSC.

**
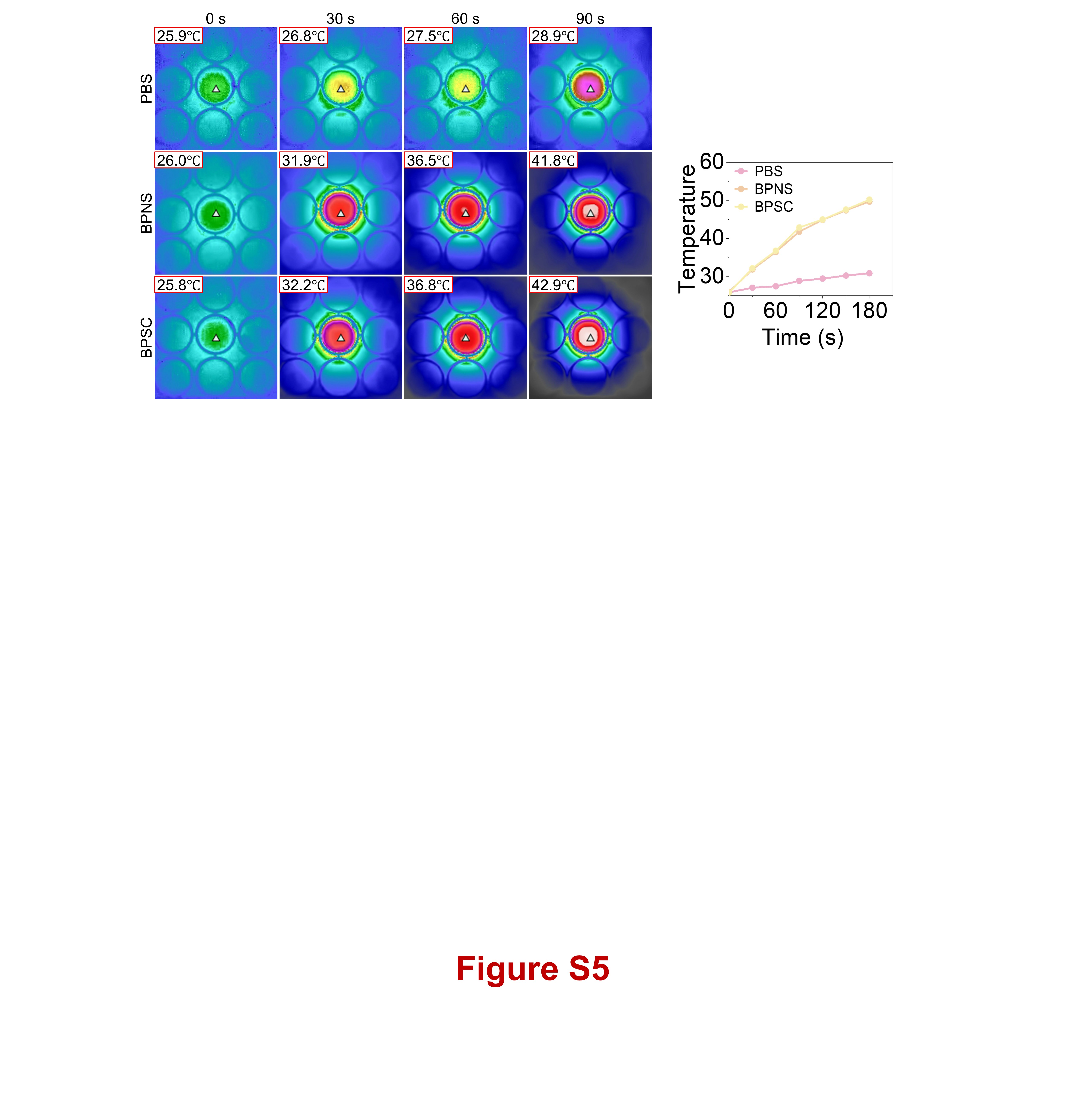
**

**S5:** Real-time thermal imaging and quantification of PBS, BPNS suspensions (100 μg/mL), and BPSC suspensions (100 μg/mL) under NIR irradiation (NIR: 808 nm, 1.25 W/cm^2^).

**
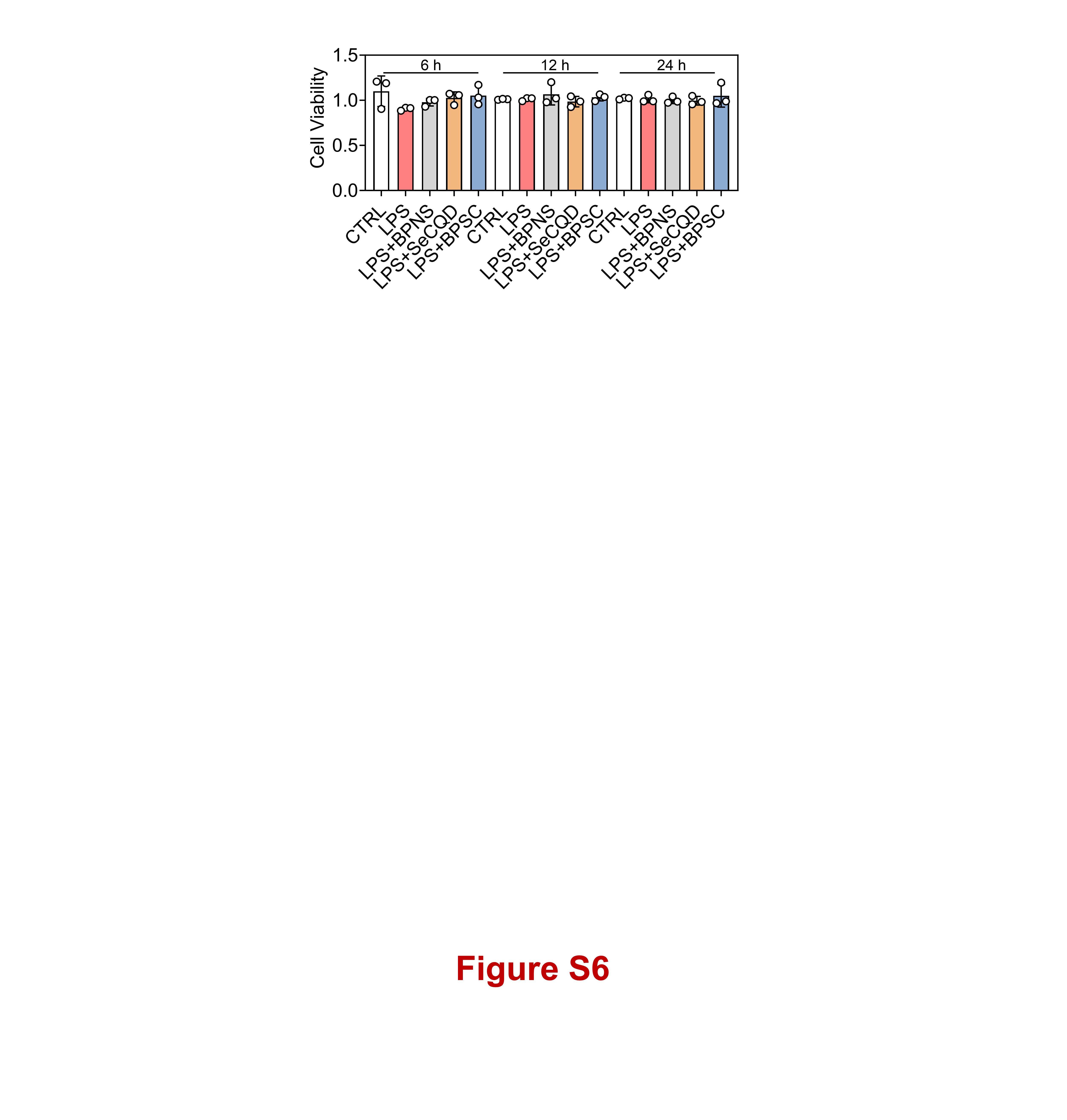
**

**S6:** Cell viability of BMDM under different conditions assessed by CCK-8 assay.

**
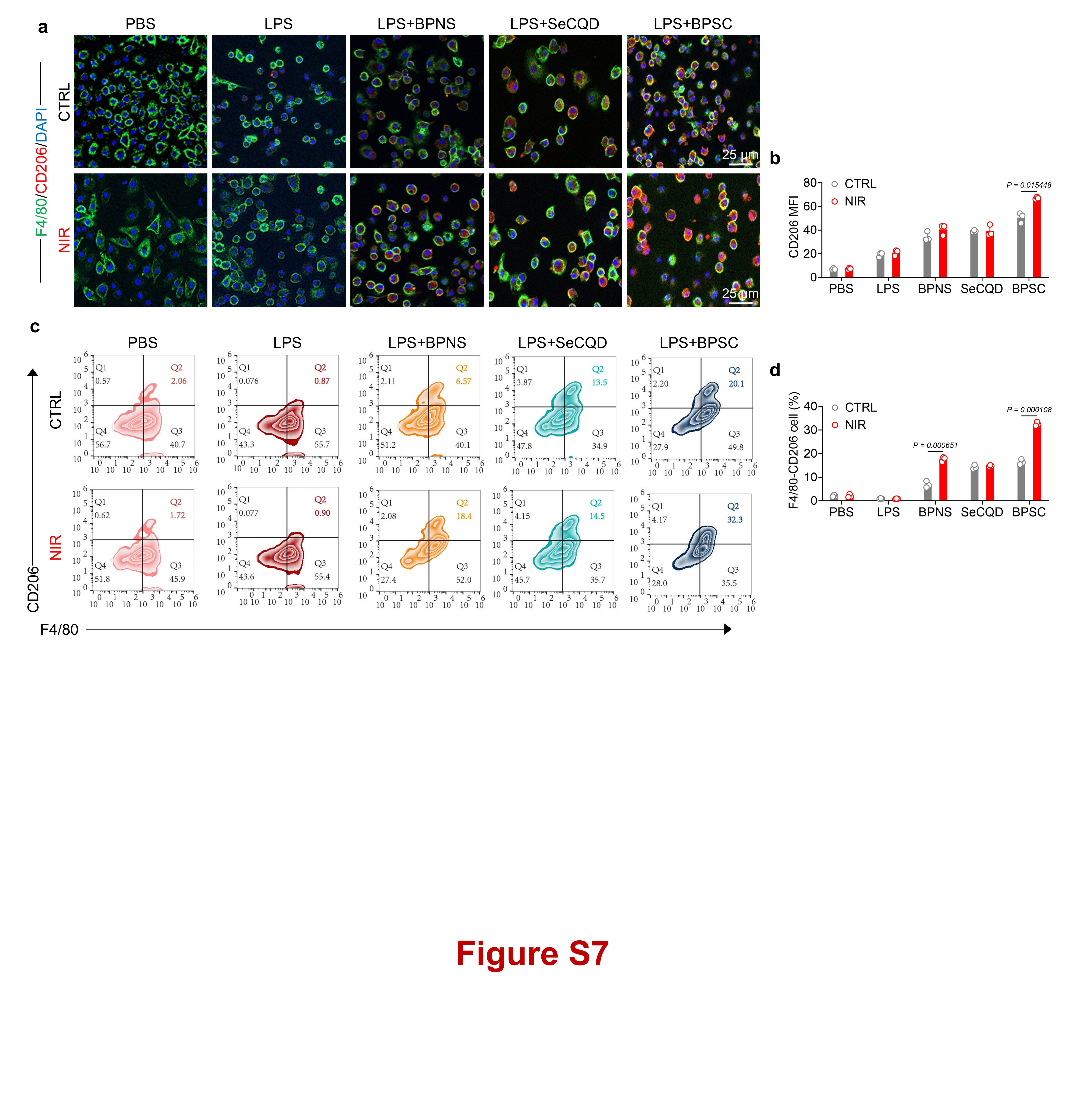
**

**S7:** a) Immunofluorescence staining and quantification of CD206 in BMDM under control treatment or near-infrared heat treatment (NIR: 808 nm, 1.25 W/cm^2^, 90 s) (n = 3). b) Representative flow cytometry plots and quantification of M2 macrophages (CD206^+^ and F4/80^+^ cells) under control treatment or near-infrared heat treatment (NIR: 808 nm, 1.25 W/cm^2^, 90 s) (n = 3).

**
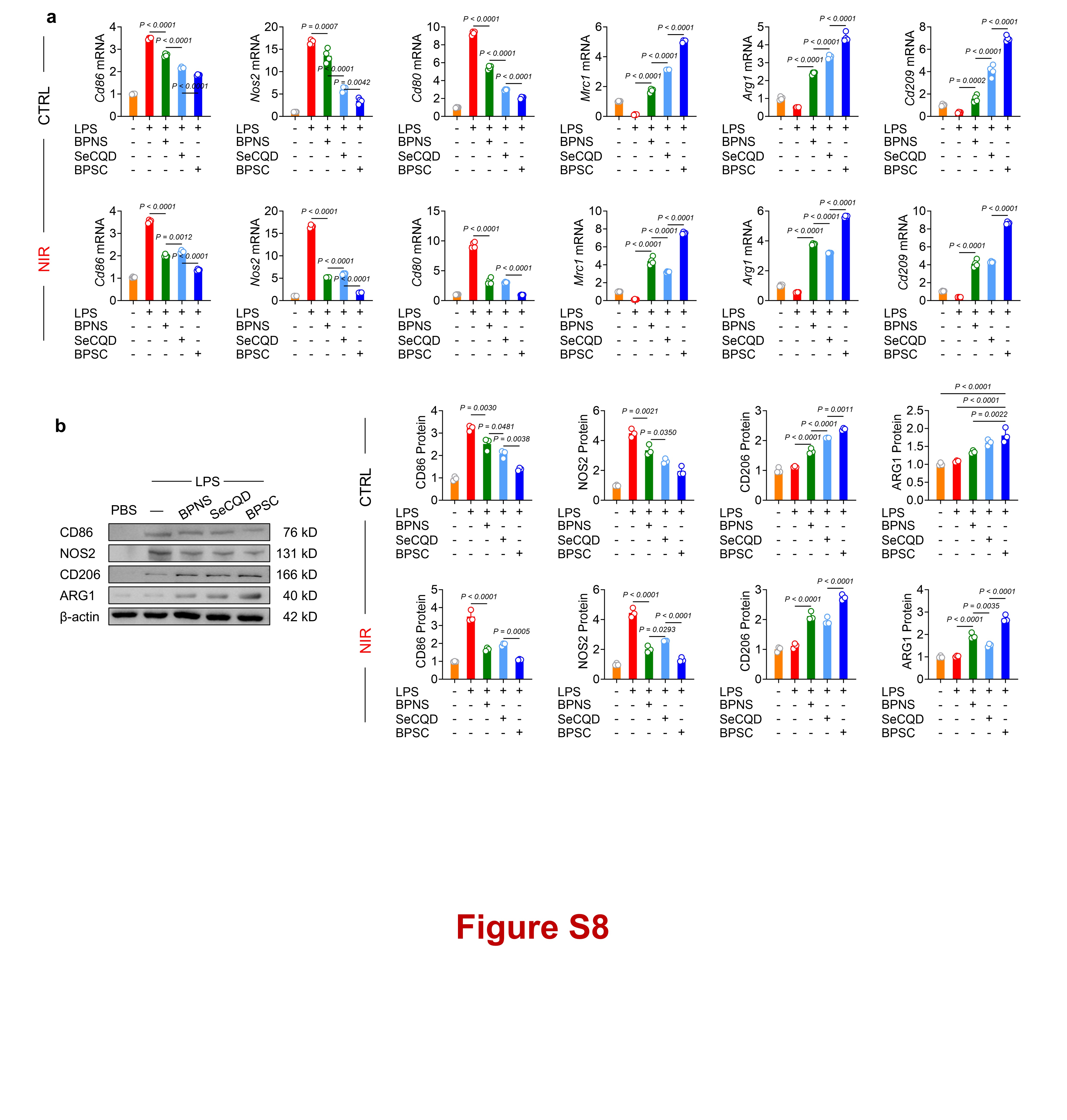
**

**S8:** a) qPCR analysis of CD86, iNOS, CD80, MRC1, Arg1, and CD209 mRNA levels in BMDM under control treatment or near-infrared heat treatment (NIR: 808 nm, 1.25 W/cm^2^, 90 s) (n = 4). b) Representative western blot bands and quantification of macrophage polarization in BMDM at different conditions (NIR: 808 nm, 1.25 W/cm^2^, 90 s) (n = 3).

**
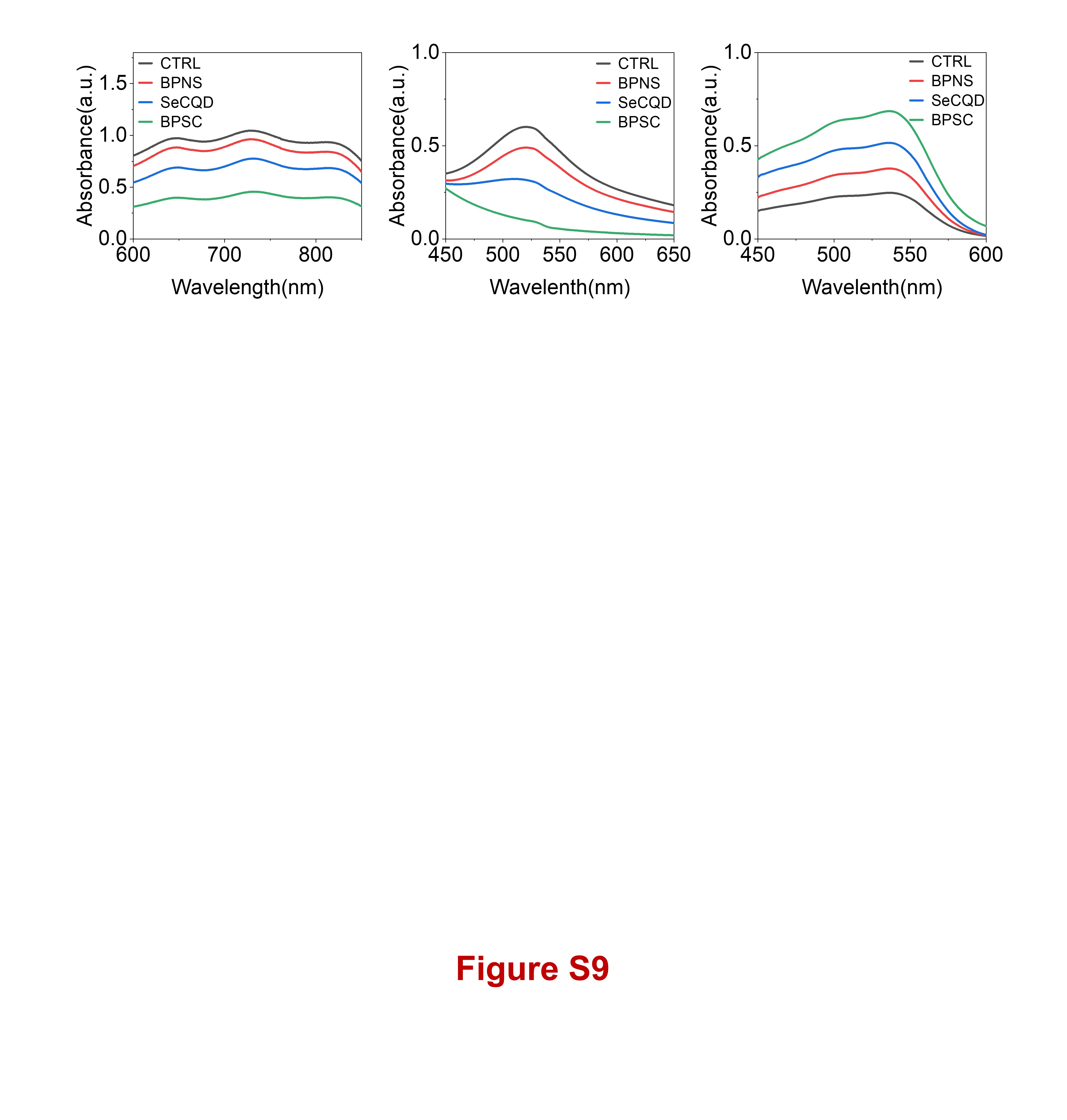
**

**S9:** The scavenging efficiency of BPNS, SeCQD, and BPSC toward typical free radicals (ABTS, DPPH) and •OH.

**
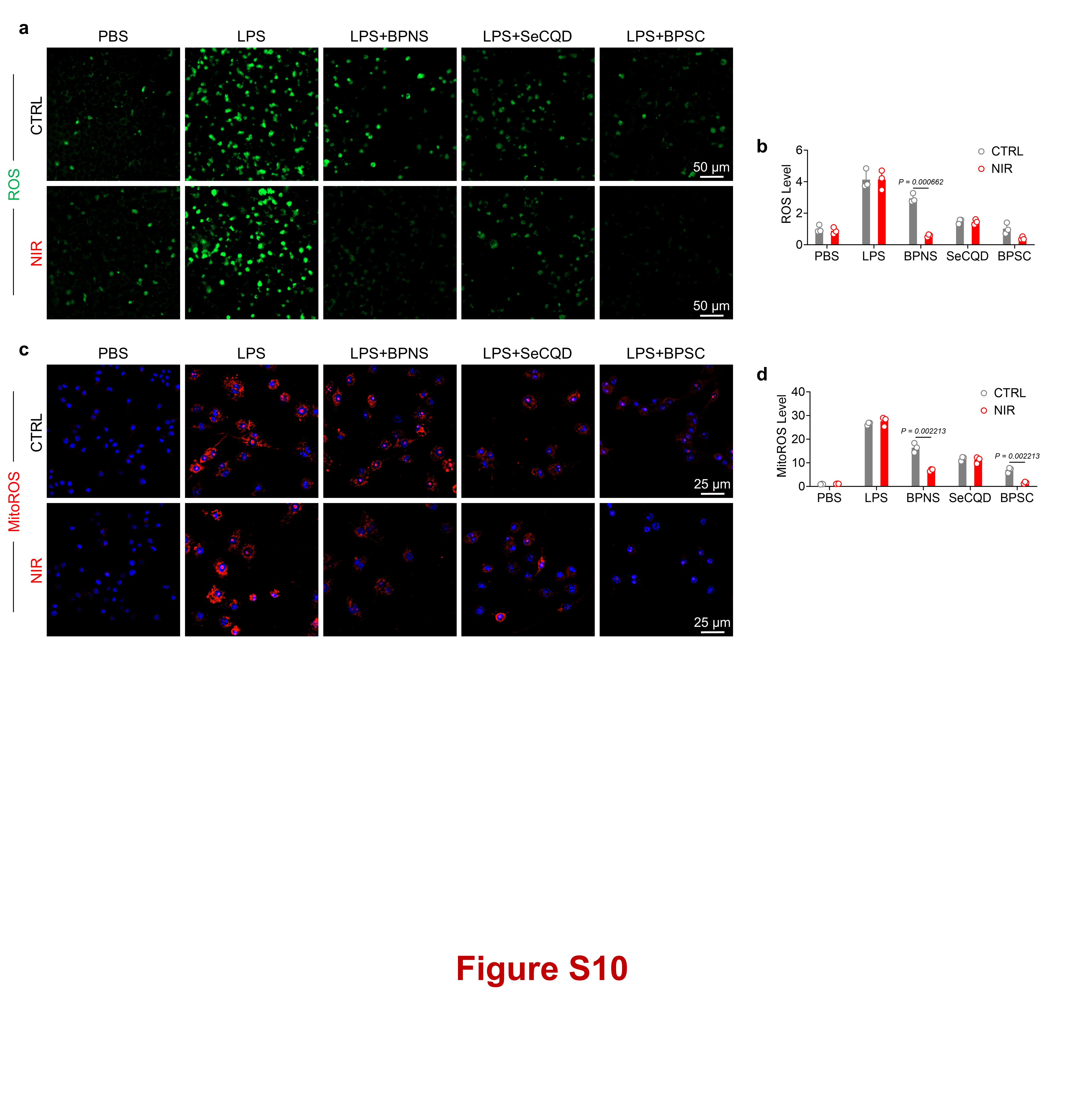
**

**S10:** ROS, mitoROS levels, and quantification of BMDM detected by DCFH-DA (green) and MitoSOX (red) under control treatment or near-infrared heat treatment (NIR: 808 nm, 1.25 W/cm^2^, 90 s) (n = 3).

**
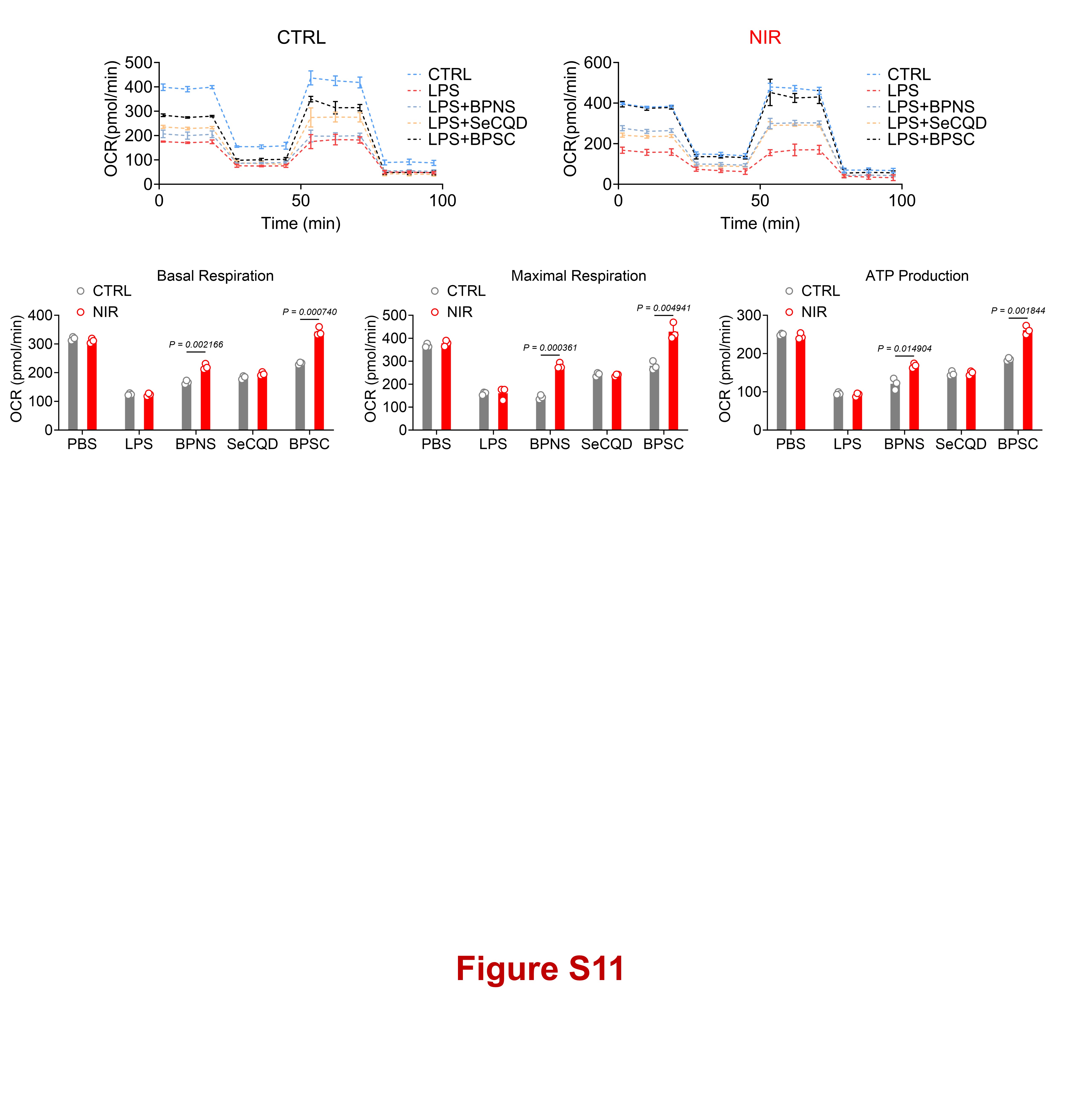
**

**S11:** Oxygen consumption rate (OCR) and quantification of BMDM under control treatment and near-infrared heat treatment (NIR: 808 nm, 1.25 W/cm^2^, 90 s) (n = 3).

**
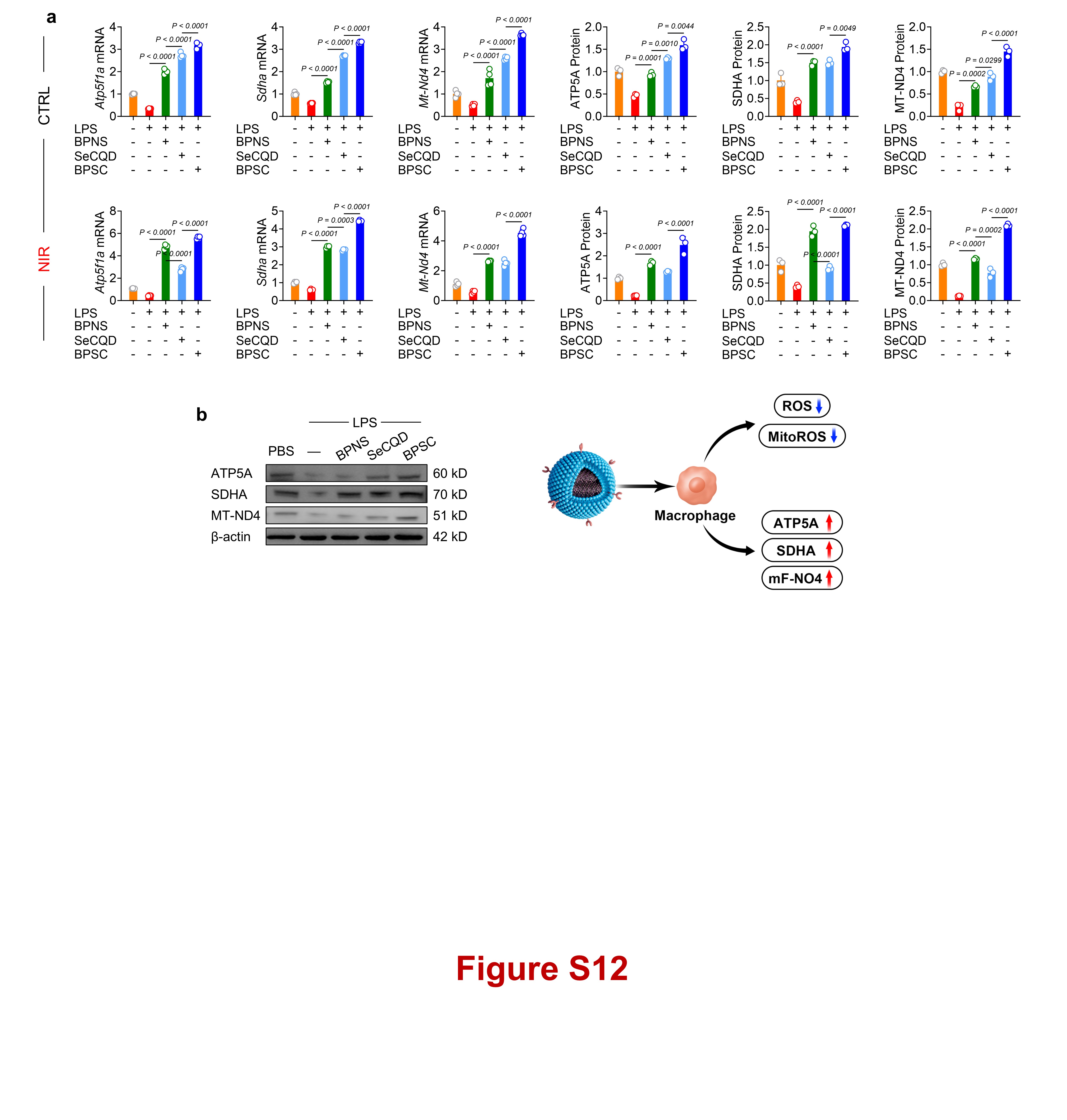
**

**S12:** a) qPCR analysis of *Atp5f1a*, *Sdha*, *Mt-Nd4* mRNA levels in BMDM under control treatment and near-infrared heat treatment (NIR: 808 nm, 1.25 W/cm^2^, 90 s) (n = 4). b) Representative western blot bands and quantification of mitochondrial function in BMDM at different conditions under control treatment and near-infrared heat treatment (NIR: 808 nm, 1.25 W/cm^2^, 90 s) (n = 3).


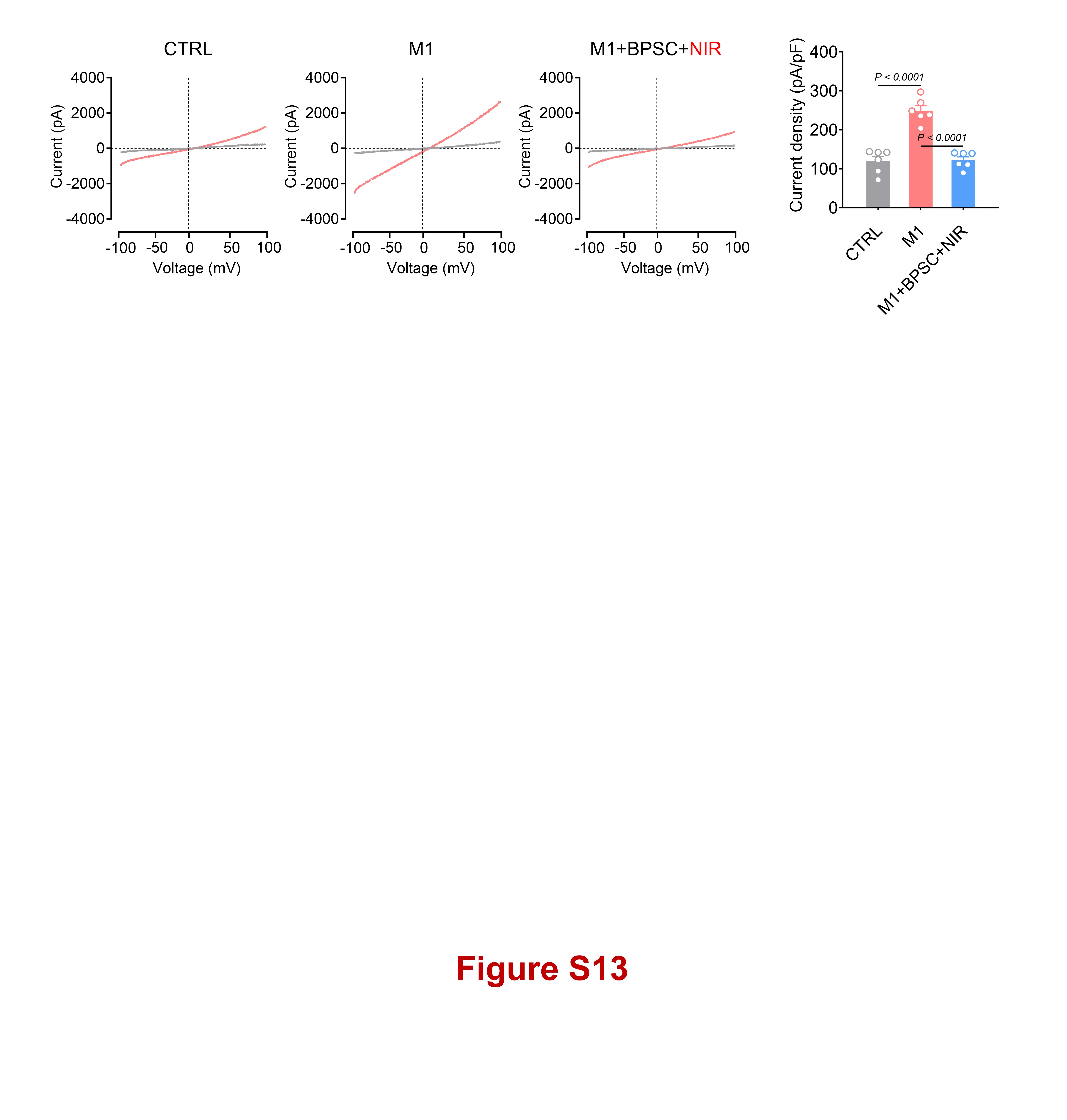


**S13:** Representative current–voltage curves and summarized current density analysis recorded in BMDMs, in which whole-cell currents were evoked by voltage ramp stimulation (−100 to +100 mV) under baseline conditions and after stimulation with GSK1016790A in CTRL, M1, and M1+BPSC+NIR groups (NIR: 808 nm, 1.25 W/cm^2^, 90 s) (n = 6).


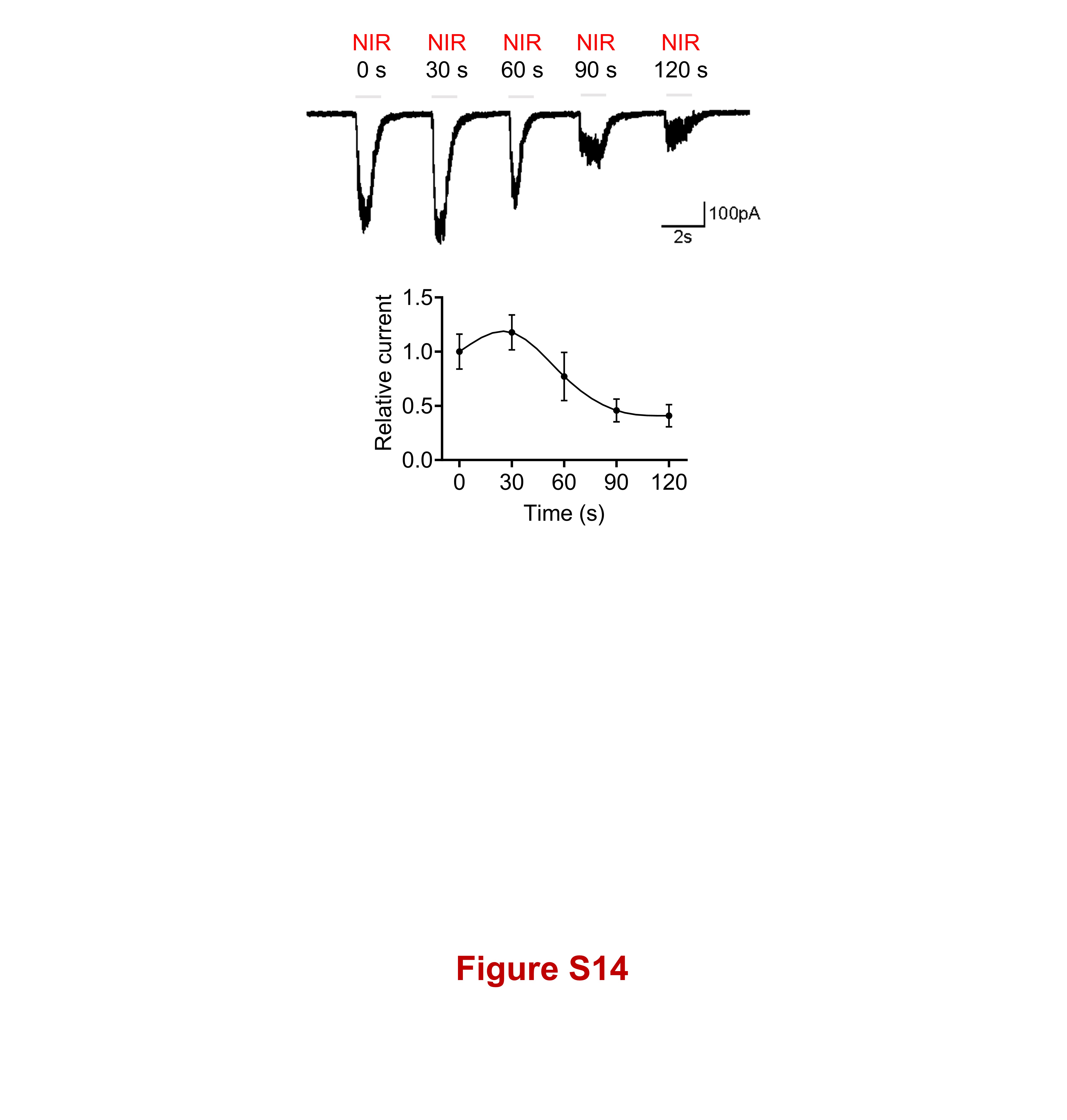


**S14:** The continuous whole-cell voltage-clamp recordings were conducted on individual BMDMs after they were treated with M2M@BPSC (n = 6).

**
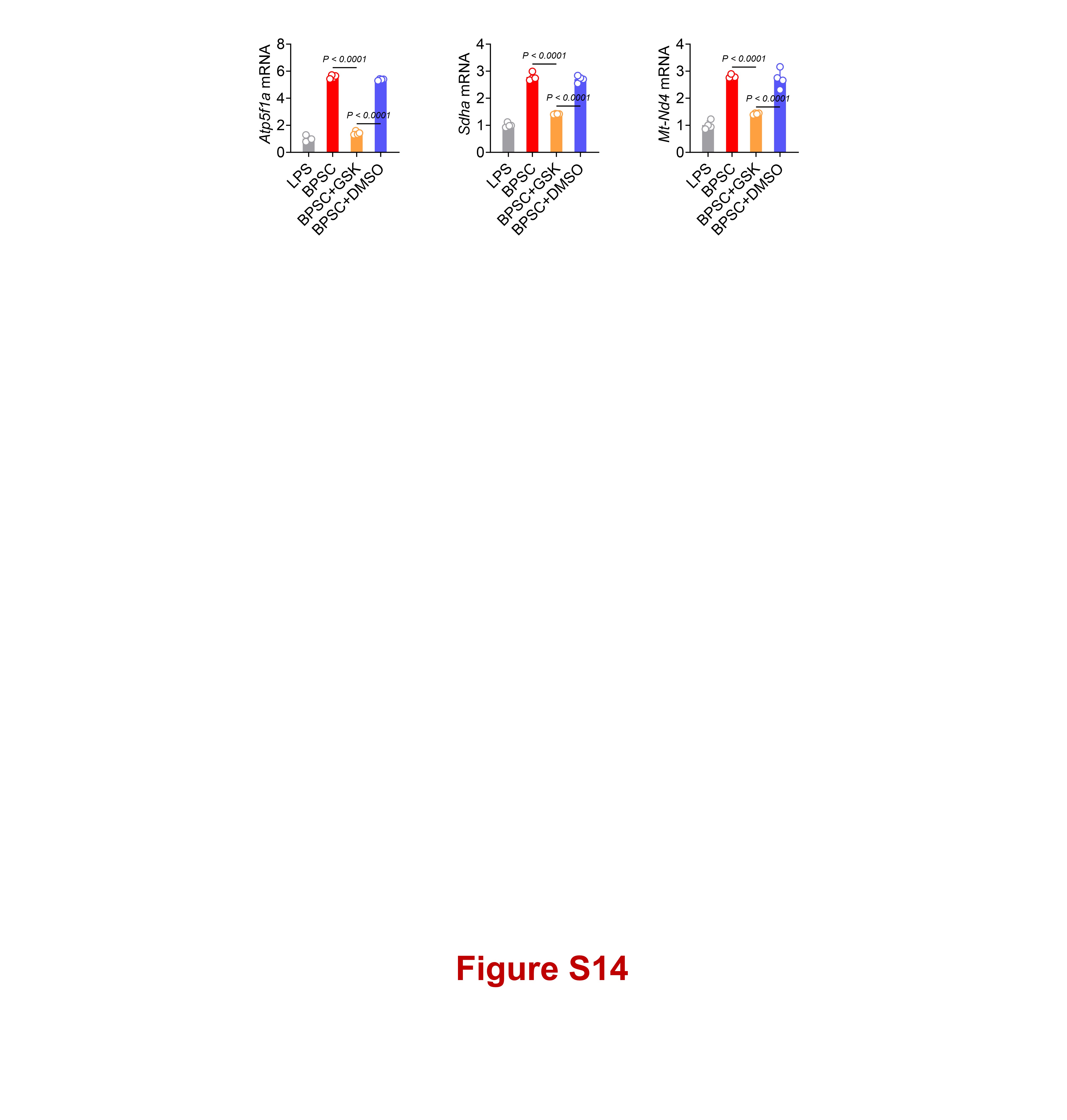
**

**S15:** qPCR analysis of *Atp5f1a*, *Sdha*, *Mt-Nd4* mRNA levels in BMDM under different conditions with near-infrared heat treatment (NIR: 808 nm, 1.25 W/cm^2^, 90 s) (n = 4).

**
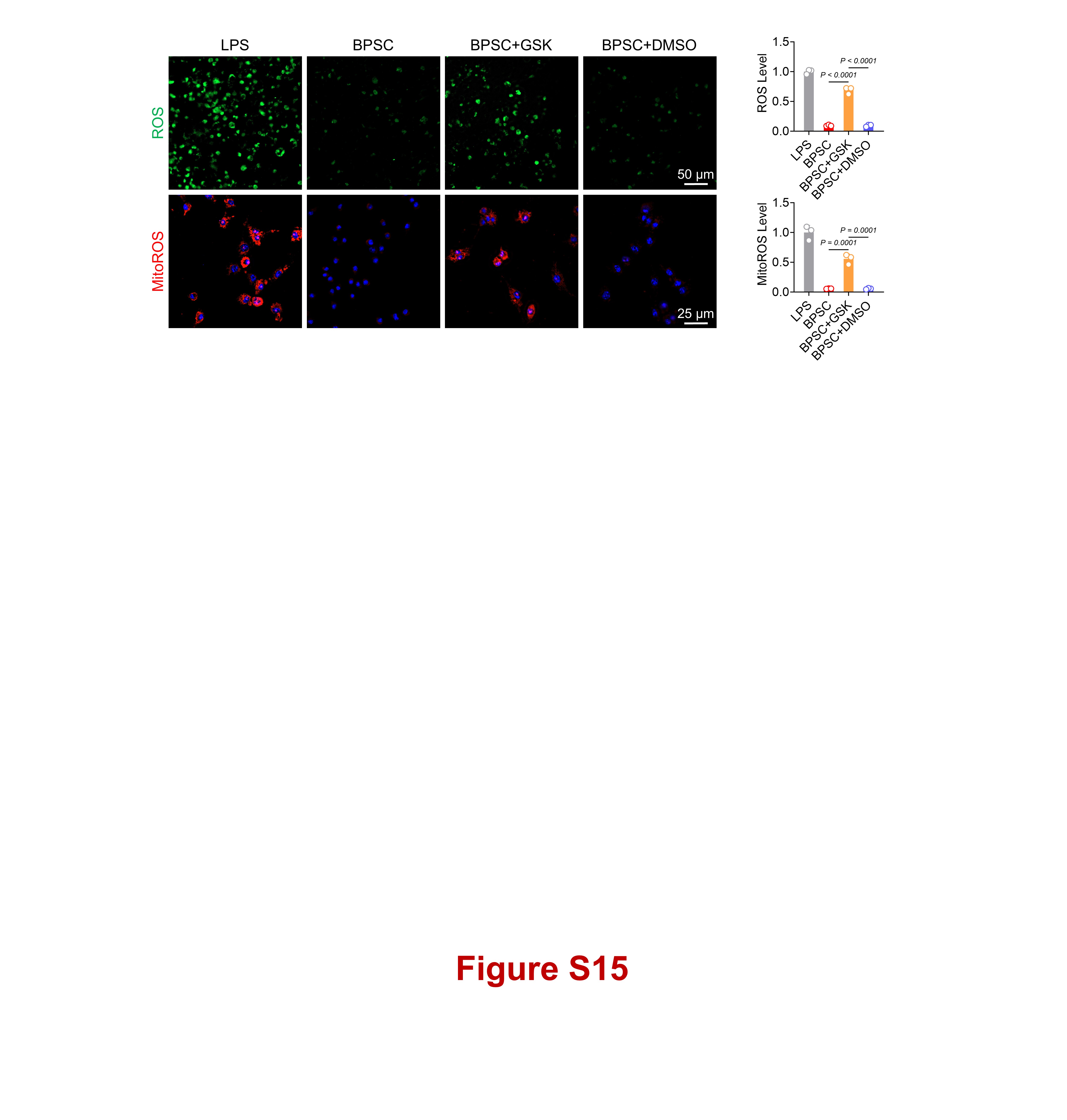
**

**S16:** ROS, mitoROS levels, and quantification of BMDM detected by DCFH-DA (green) and MitoSOX (red) in different conditions under near-infrared heat treatment (NIR: 808 nm, 1.25 W/cm^2^, 90 s) (n = 3).

**
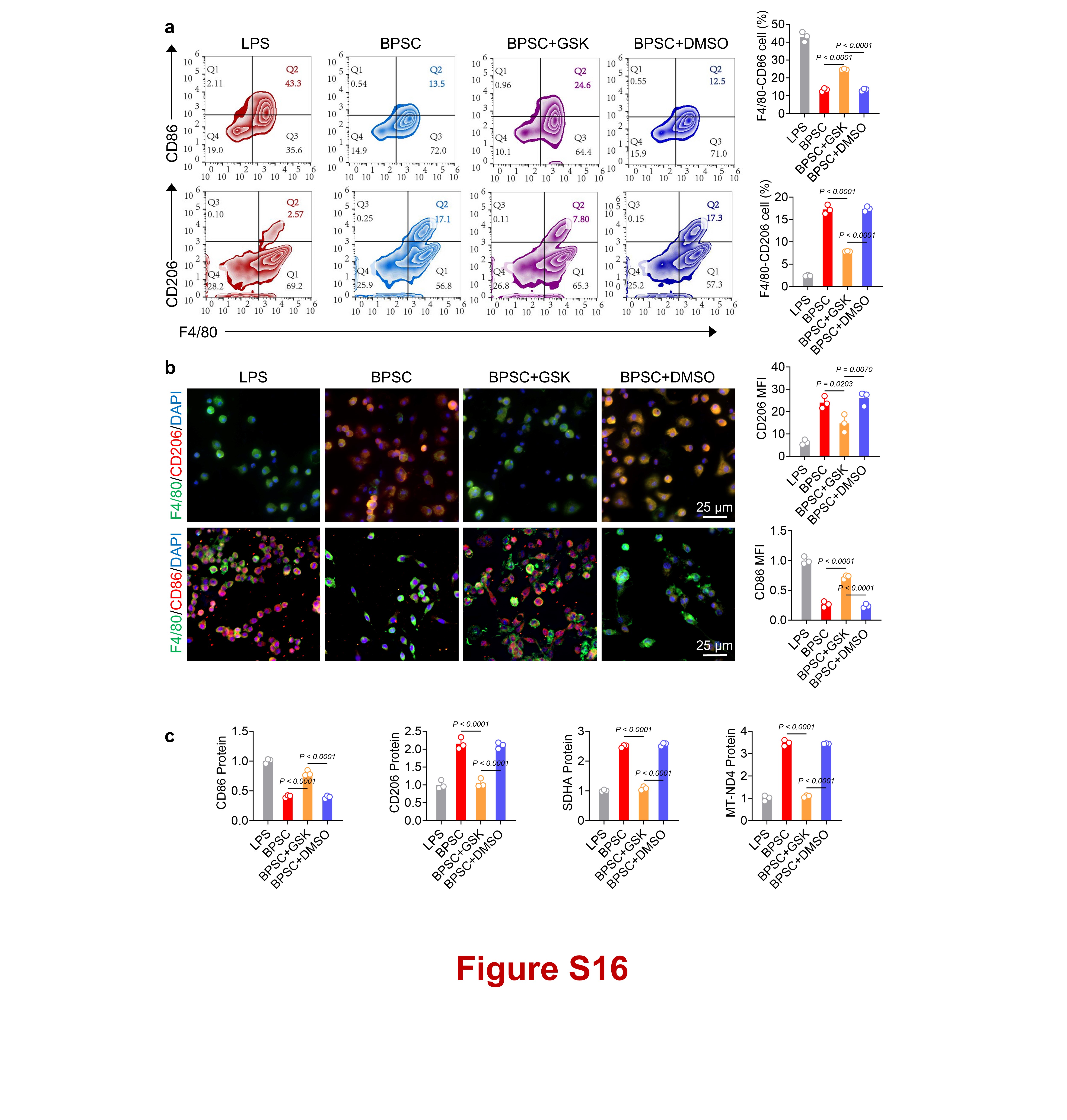
**

**S17:** a) Representative flow cytometry plots and quantification of M1 macrophages (CD86^+^ and F4/80^+^ cells) and M2 macrophages (CD206^+^ and F4/80^+^ cells) in different conditions under near-infrared heat treatment (NIR: 808 nm, 1.25 W/cm^2^, 90 s) (n = 3). b) Immunofluorescence staining and quantification of CD206 and CD86 in BMDM under control treatment and near-infrared heat treatment (NIR: 808 nm, 1.25 W/cm^2^, 90 s) (n = 3). c) Western blot bands quantification of CD86, CD206, SDHA and MT-ND4 expression in BMDM under near-infrared heat treatment (NIR: 808 nm, 1.25 W/cm^2^, 90 s) (n = 3).

**
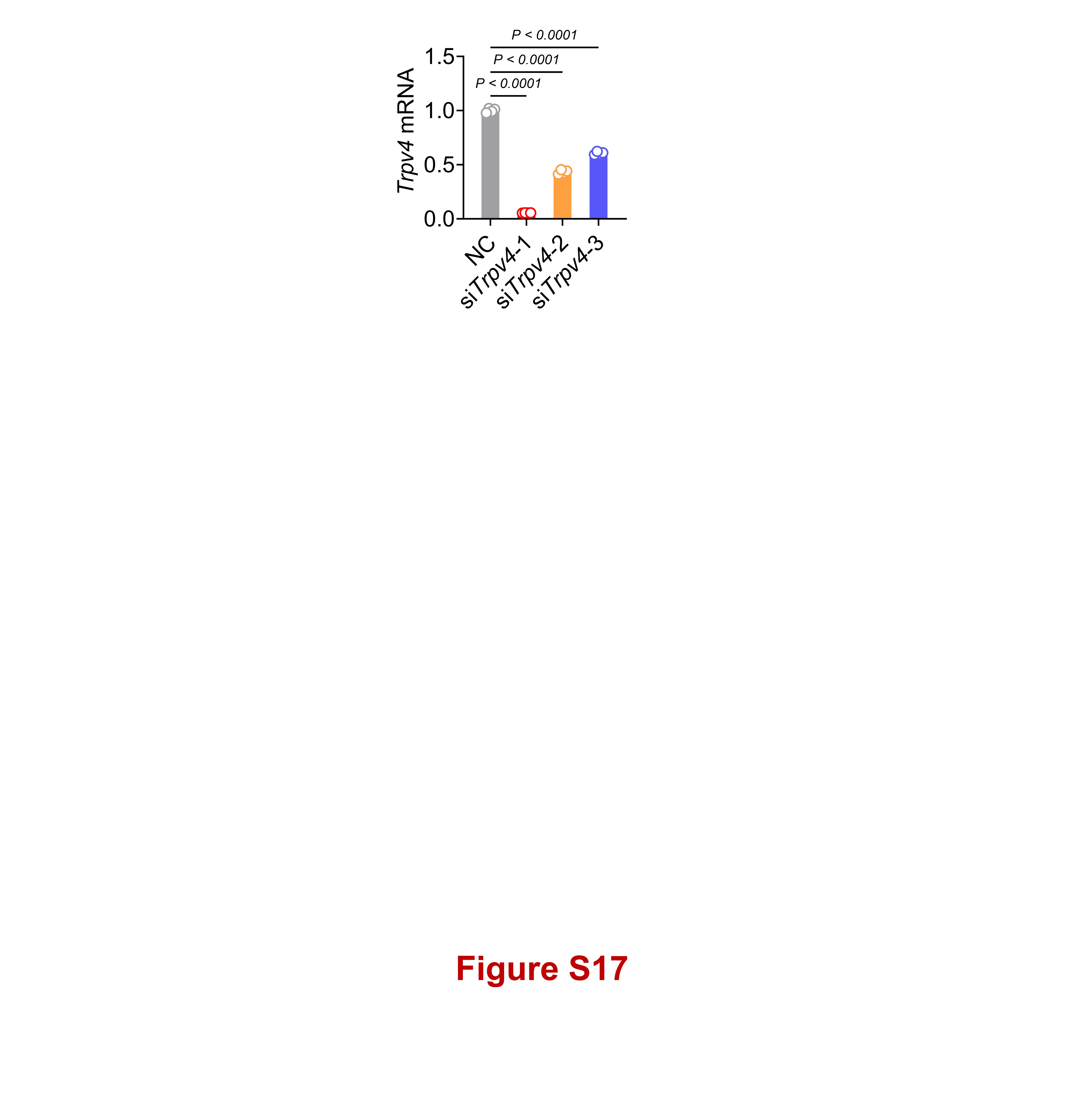
**

**S18:** Validation of siRNA-mediated inhibition of TRPV4 in BMDM (n = 4).


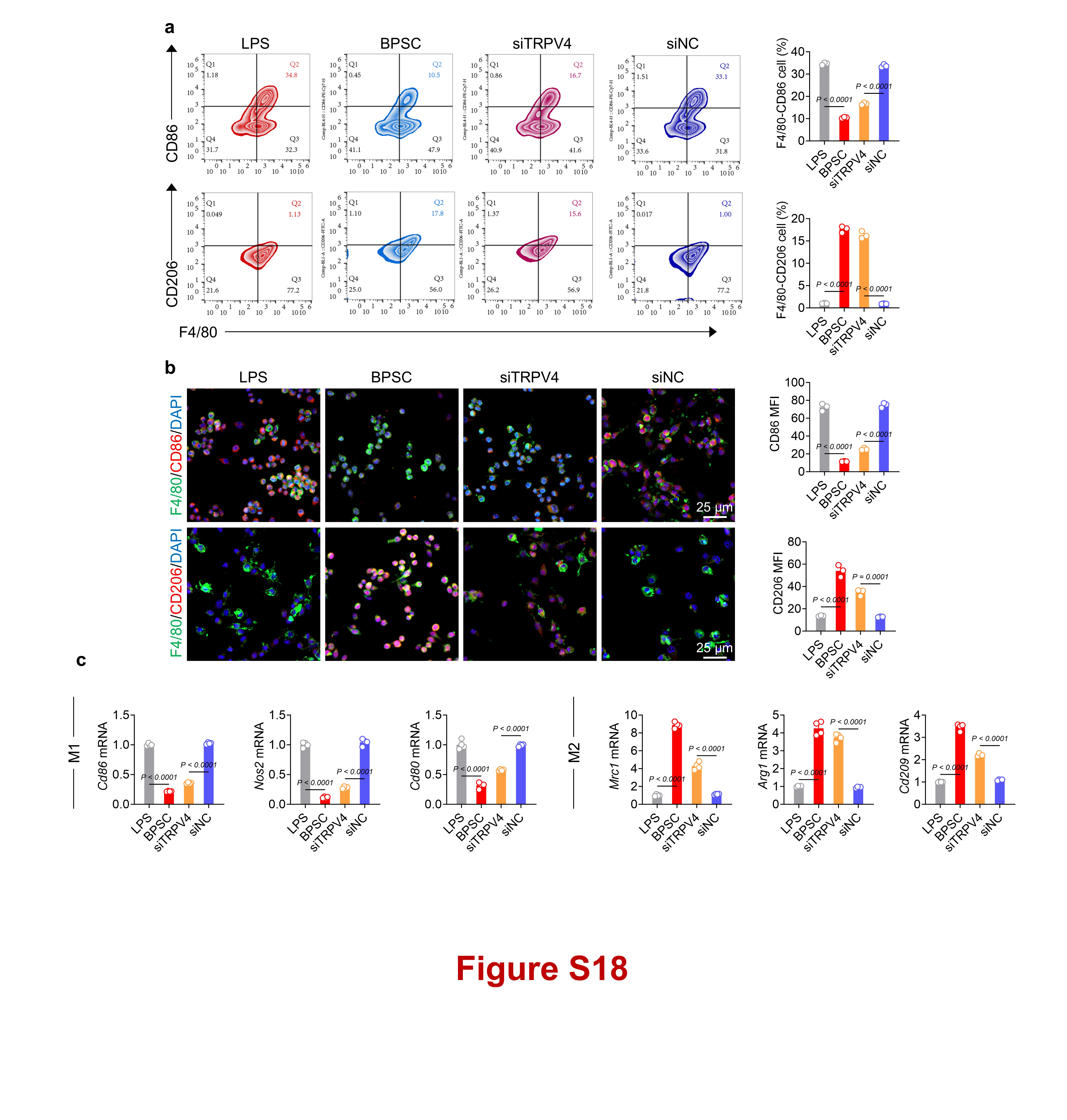


**S19:** Representative flow cytometry plots and quantification of M1 macrophages (CD86^+^ and F4/80^+^ cells) and M2 macrophages (CD206^+^ and F4/80^+^ cells) in different conditions under near-infrared heat treatment (NIR: 808 nm, 1.25 W/cm^2^, 90 s) (n = 3). b) Immunofluorescence staining and quantification of CD206 and CD86 in BMDM under control treatment and near-infrared heat treatment (NIR: 808 nm, 1.25 W/cm^2^, 90 s) (n = 3). c) qPCR analysis of *Cd86*, *Nos2*, *Cd80*, *Cd206*, *Arg1* and *Cd209* mRNA levels in BMDM under near-infrared heat treatment (NIR: 808 nm, 1.25 W/cm^2^, 90 s) (n = 4).


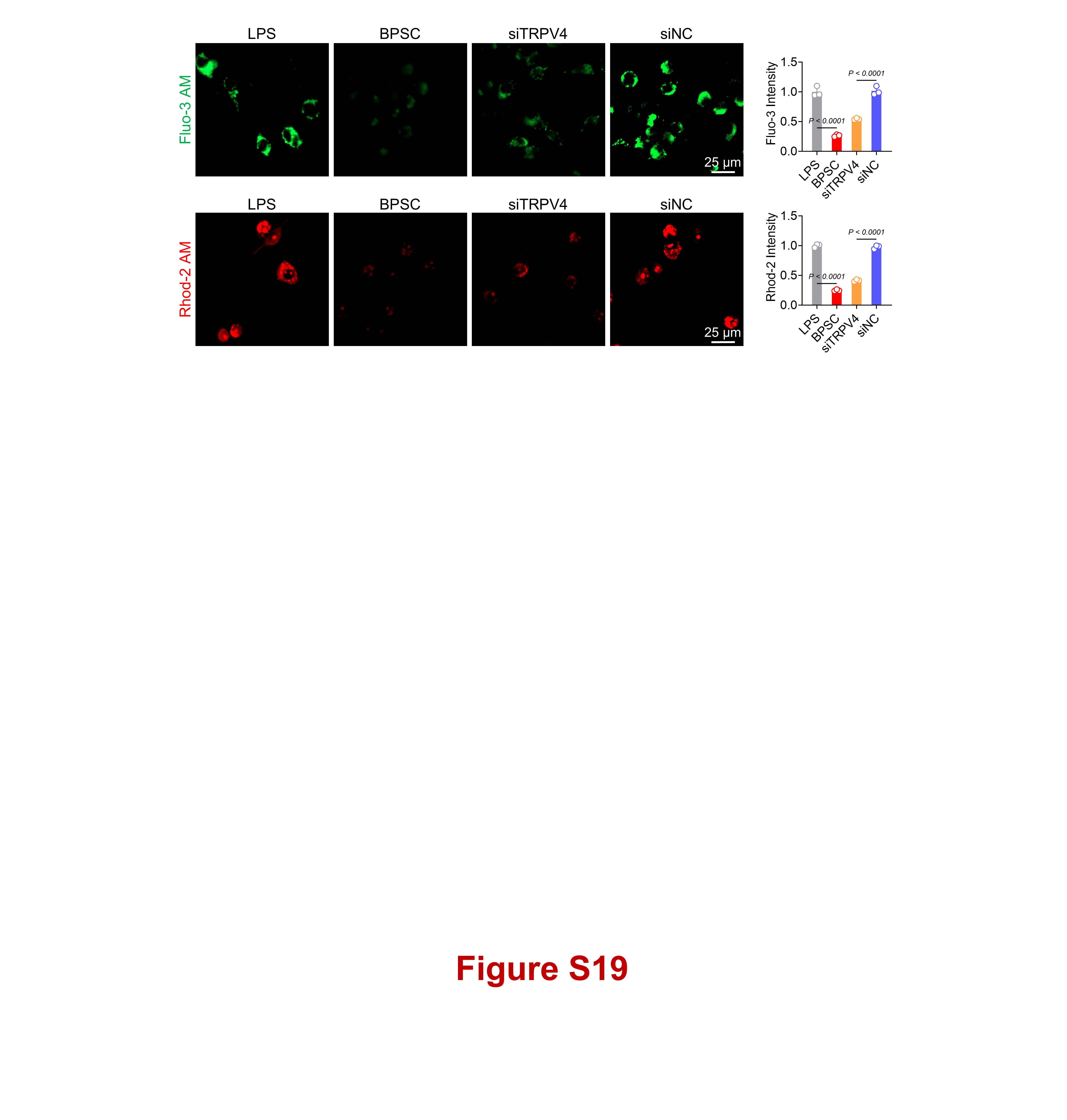


**S20:** Representative images and quantitative analysis of intracellular Ca^2+^ localization (Fluo-3 AM, green) and mitochondrial Ca^2+^ localization (Rhod-2 AM, red) are presented (scale bar: 25 μm) (n = 3).

**
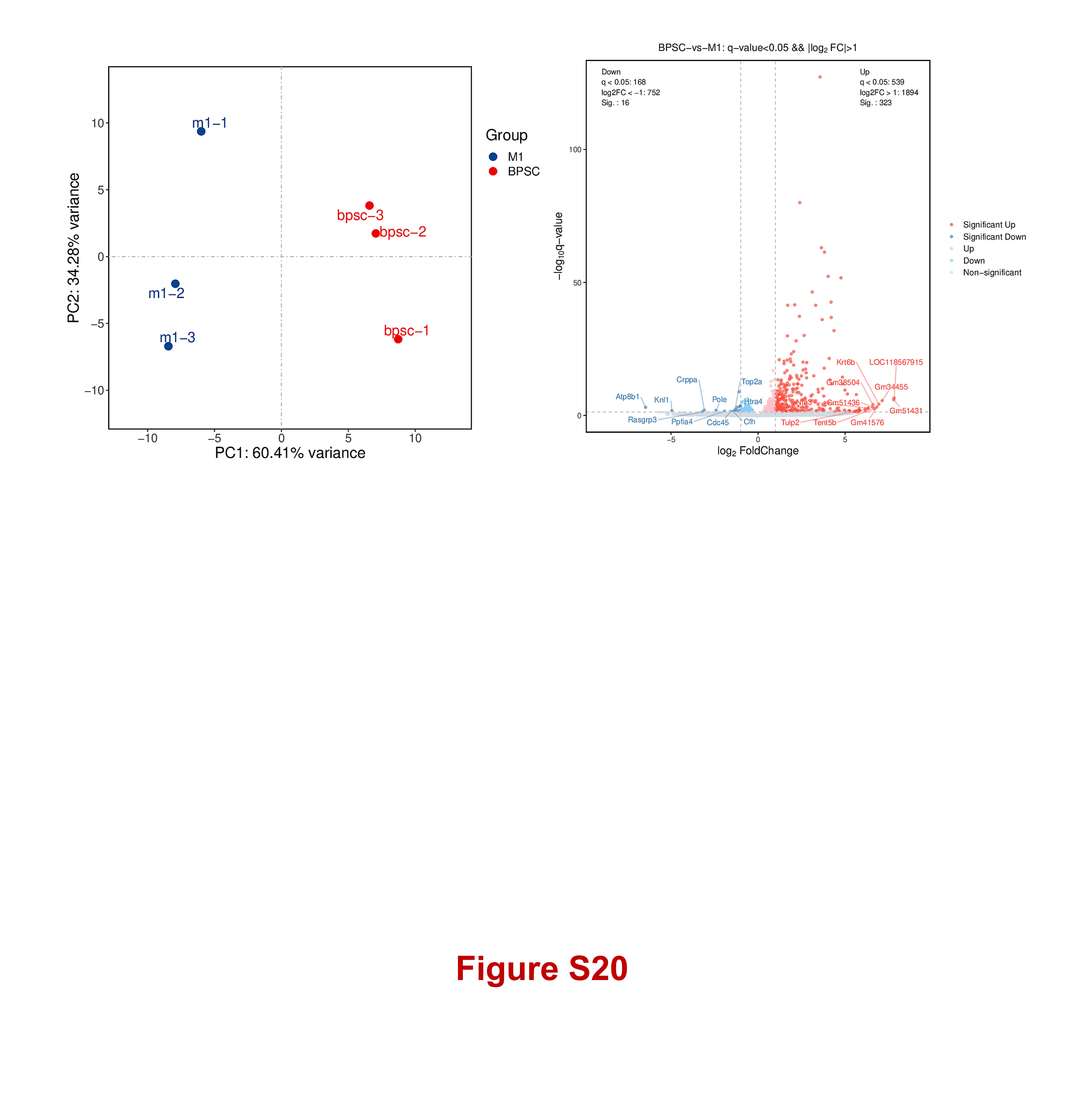
**

**S21:** a) PCA score plot showing clustering of samples by M1-BMDM vs. M1-BMDM with BPSC+NIR. b) Volcano plot of differentially expressed genes (DEGs) by M1-BMDM vs. M1-BMDM with BPSC+NIR.

**
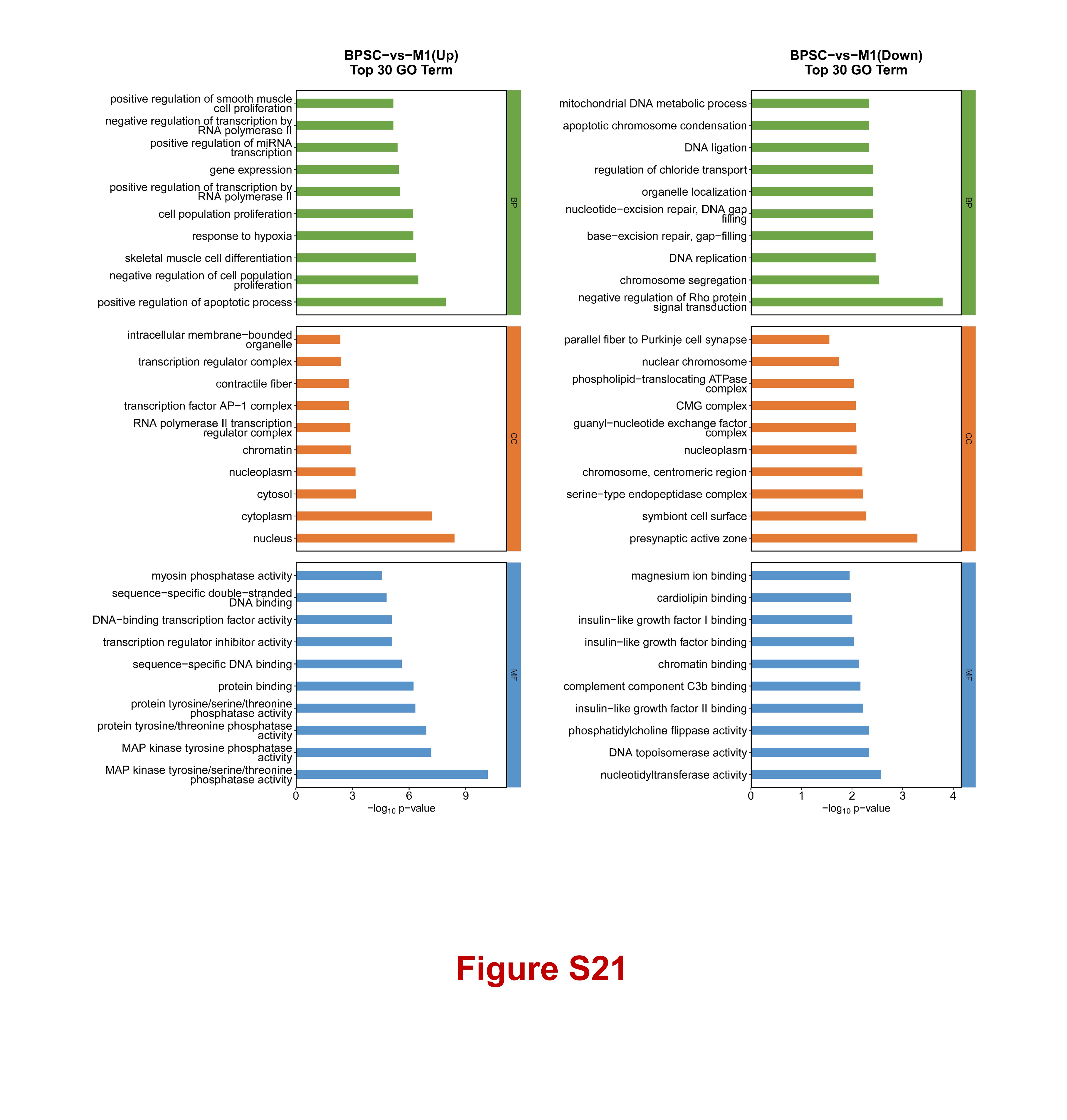
**

**S22:** Gene Ontology (GO) enrichment analysis of M1-BMDM vs. M1-BMDM with BPSC+NIR.

**
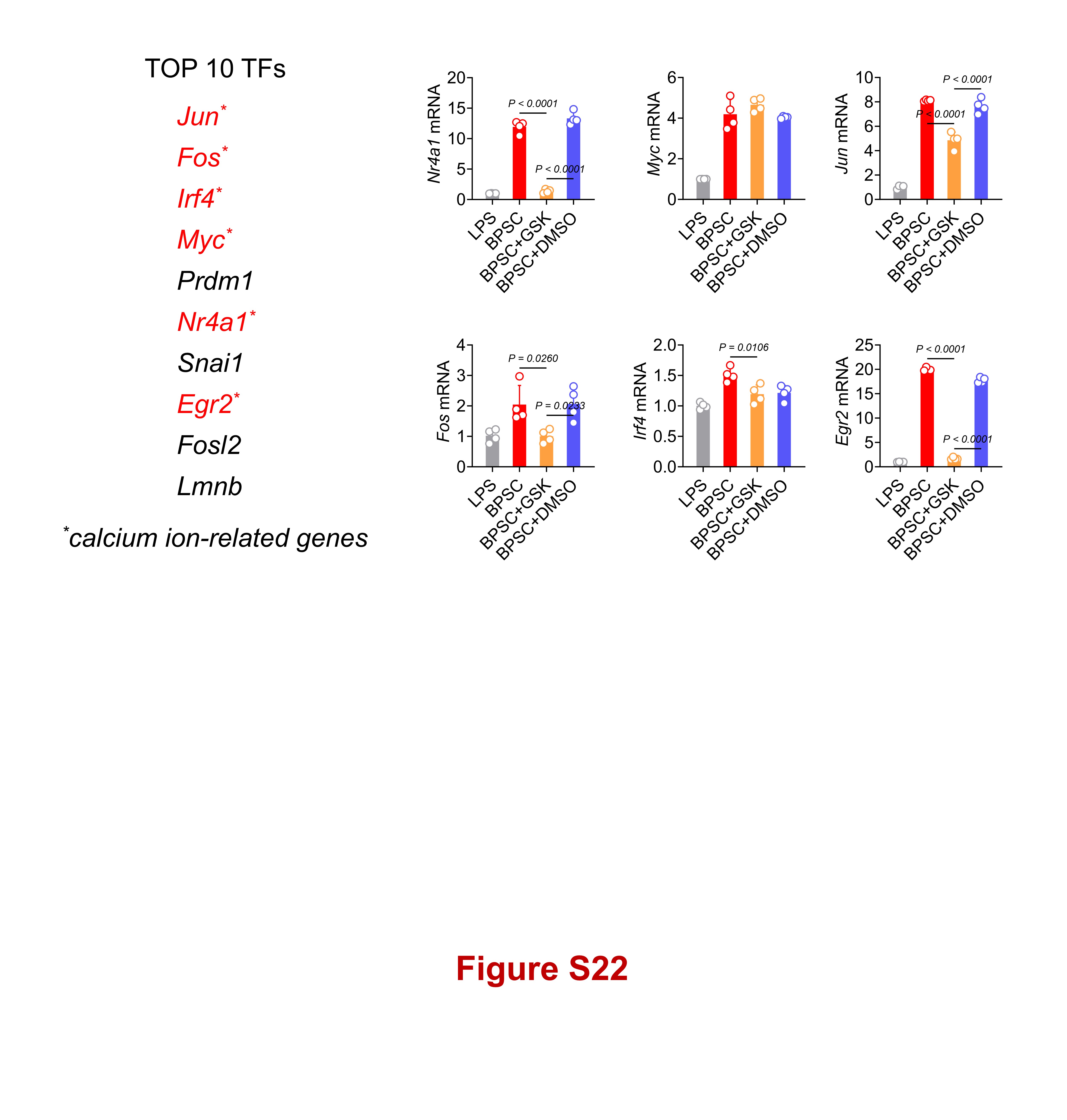
**

**S23:** Top 10 transcription factors and qPCR validation of six calcium ion-related genes (n = 4).

**
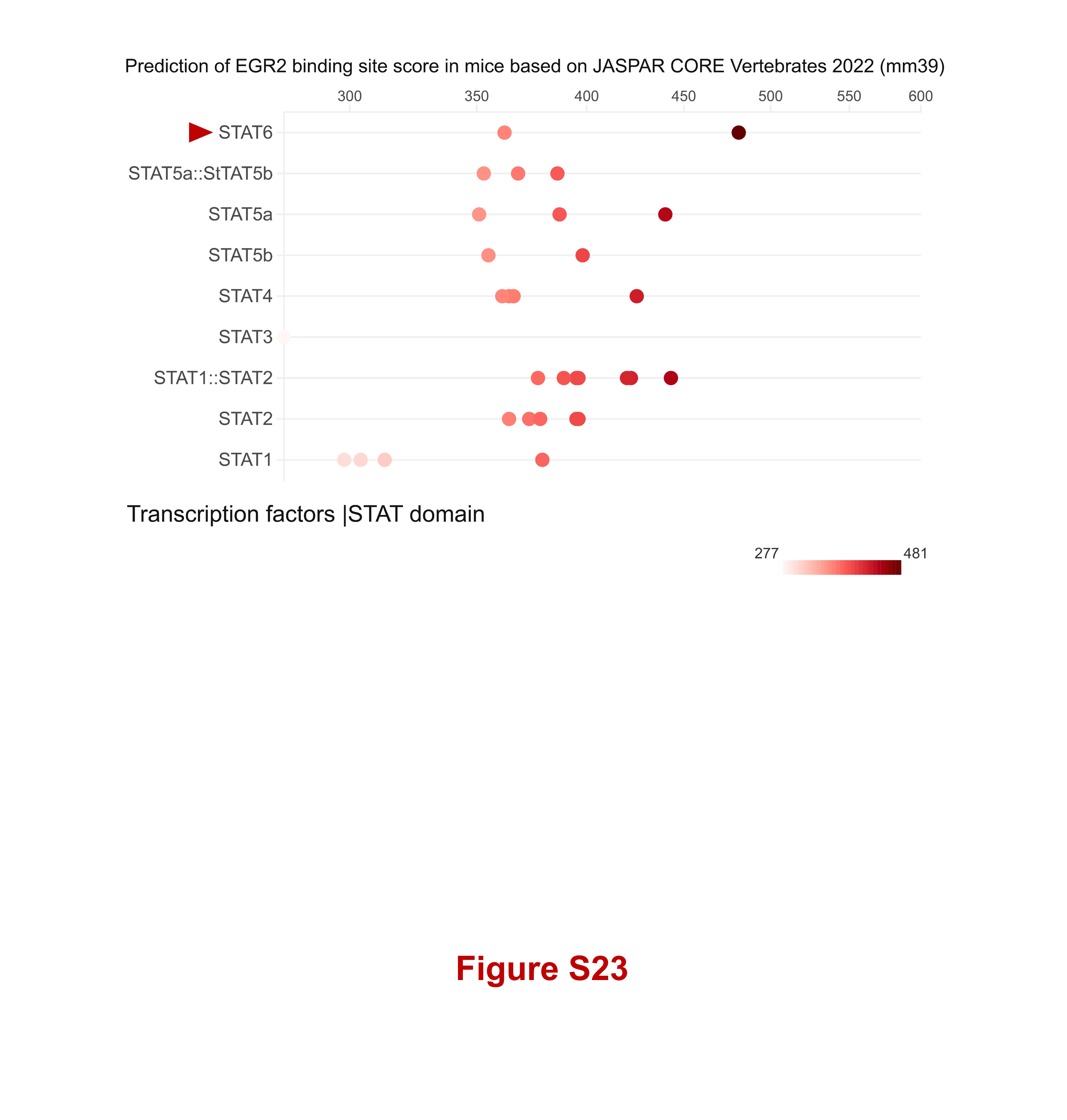
**

**S24:** ChiP-Atlas MACS2 Binding Score.


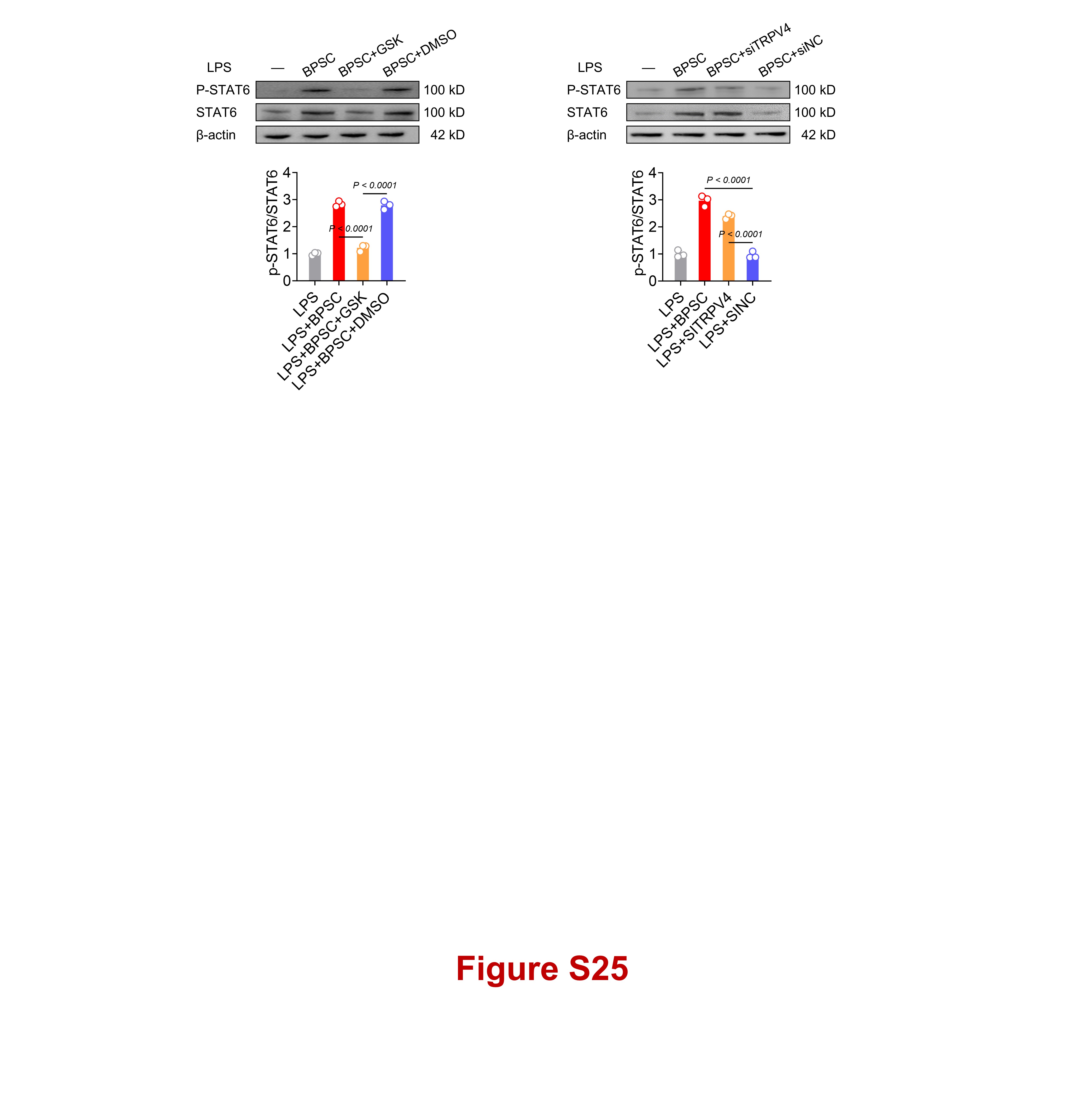


**S25:** Representative western blot bands and quantification of P-STAT6 and total STAT6 expression in BMDM at different conditions under near-infrared heat treatment (NIR: 808 nm, 1.25 W/cm^2^, 90 s) (n = 3).

**
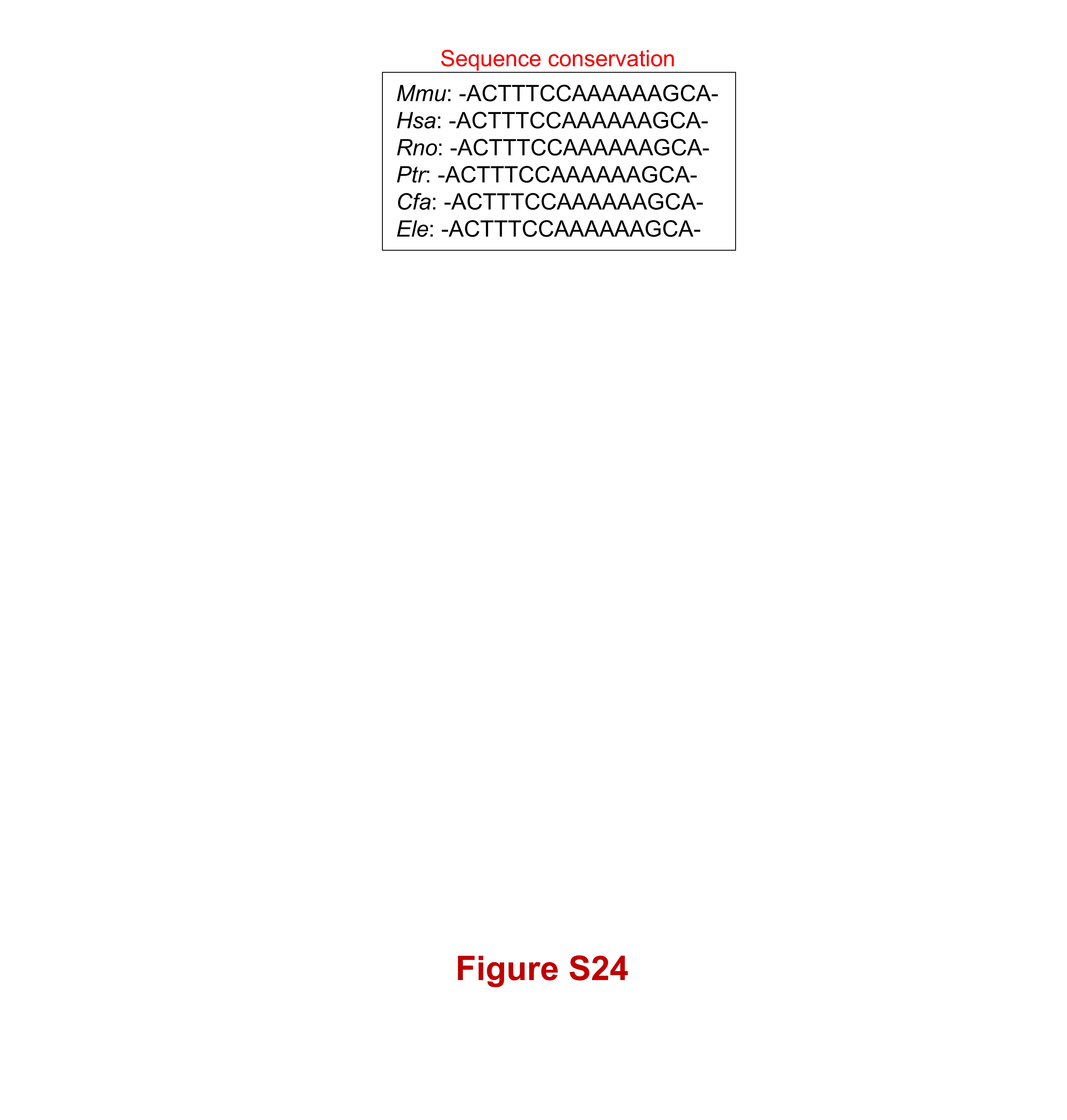
**

**S26:** Putative conserved STAT6 binding site within the mammalian EGR2 gene.

**
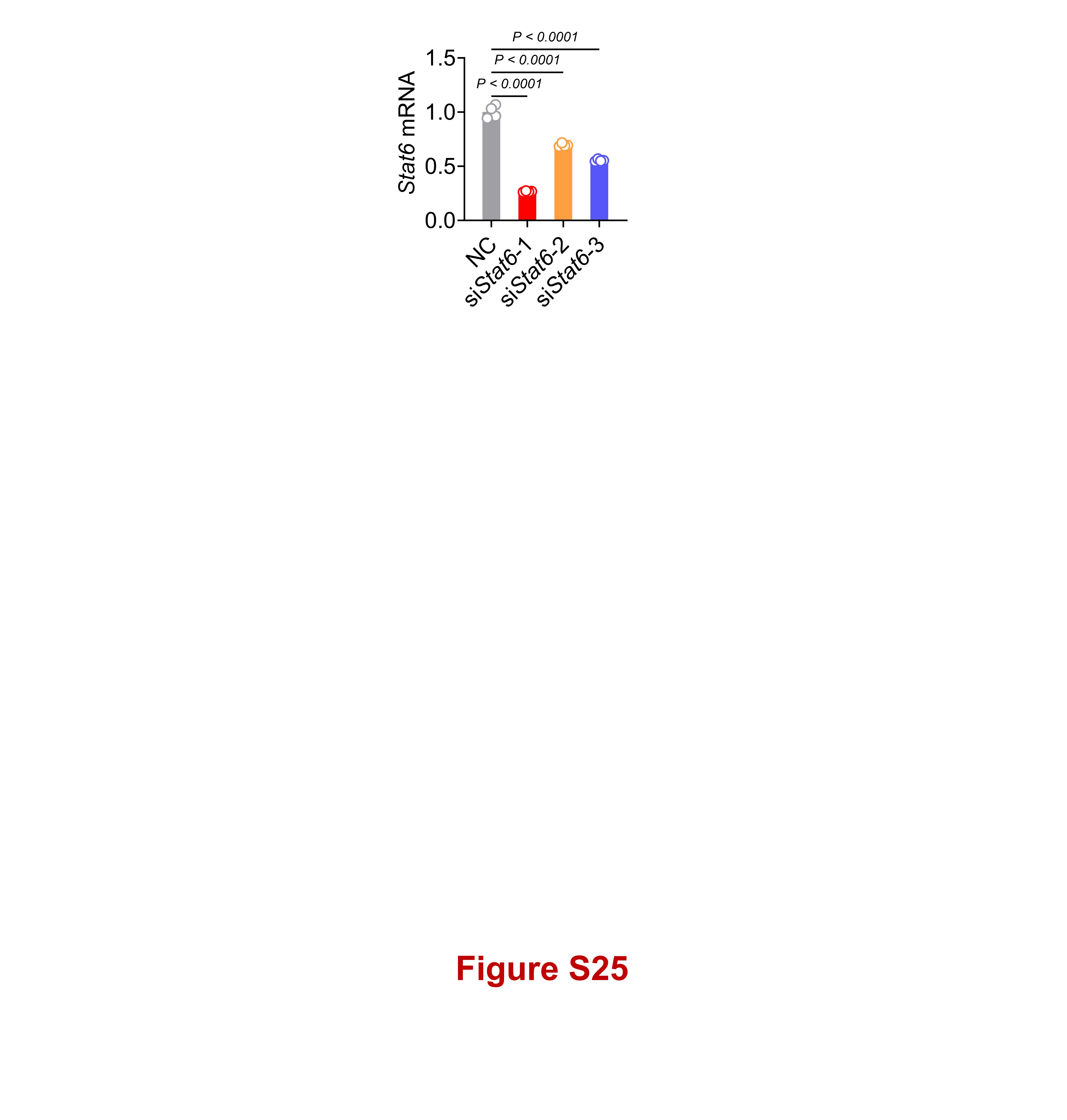
**

**S27:** Validation of siRNA-mediated inhibition of STAT6 in BMDM (n = 4).

**
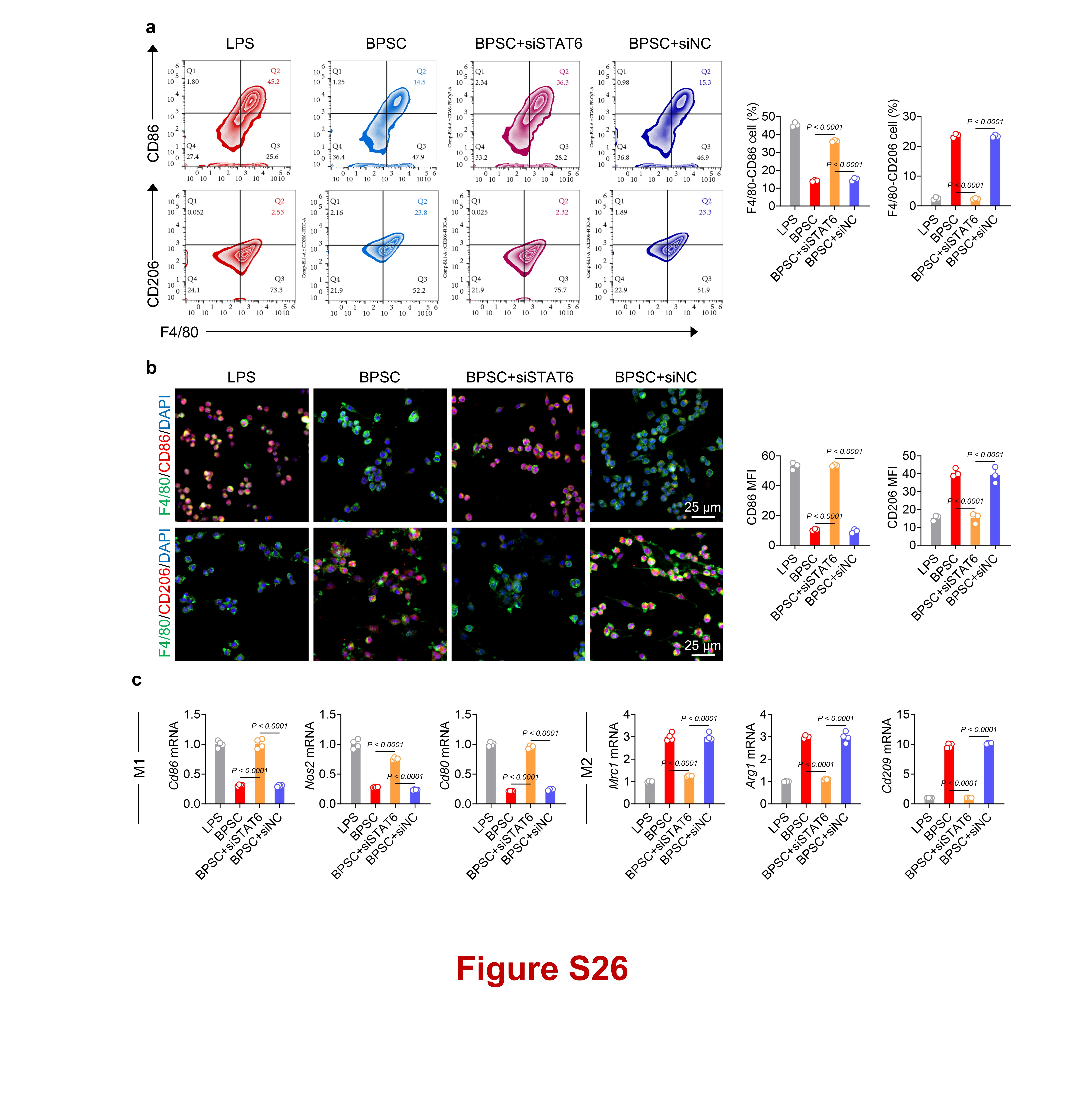
**

**S28:** Representative flow cytometry plots and quantification of M1 macrophages (CD86^+^ and F4/80^+^ cells) and M2 macrophages (CD206^+^ and F4/80^+^ cells) in different conditions under near-infrared heat treatment (NIR: 808 nm, 1.25 W/cm^2^, 90 s) (n = 3). b) Immunofluorescence staining and quantification of CD206 and CD86 in BMDM under control treatment and near-infrared heat treatment (NIR: 808 nm, 1.25 W/cm^2^, 90 s) (n = 3). c) qPCR analysis of *Cd86*, *Nos2*, *Cd80*, *Cd206*, *Arg1* and *Cd209* mRNA levels in BMDM under near-infrared heat treatment (NIR: 808 nm, 1.25 W/cm^2^, 90 s) (n = 4).

**
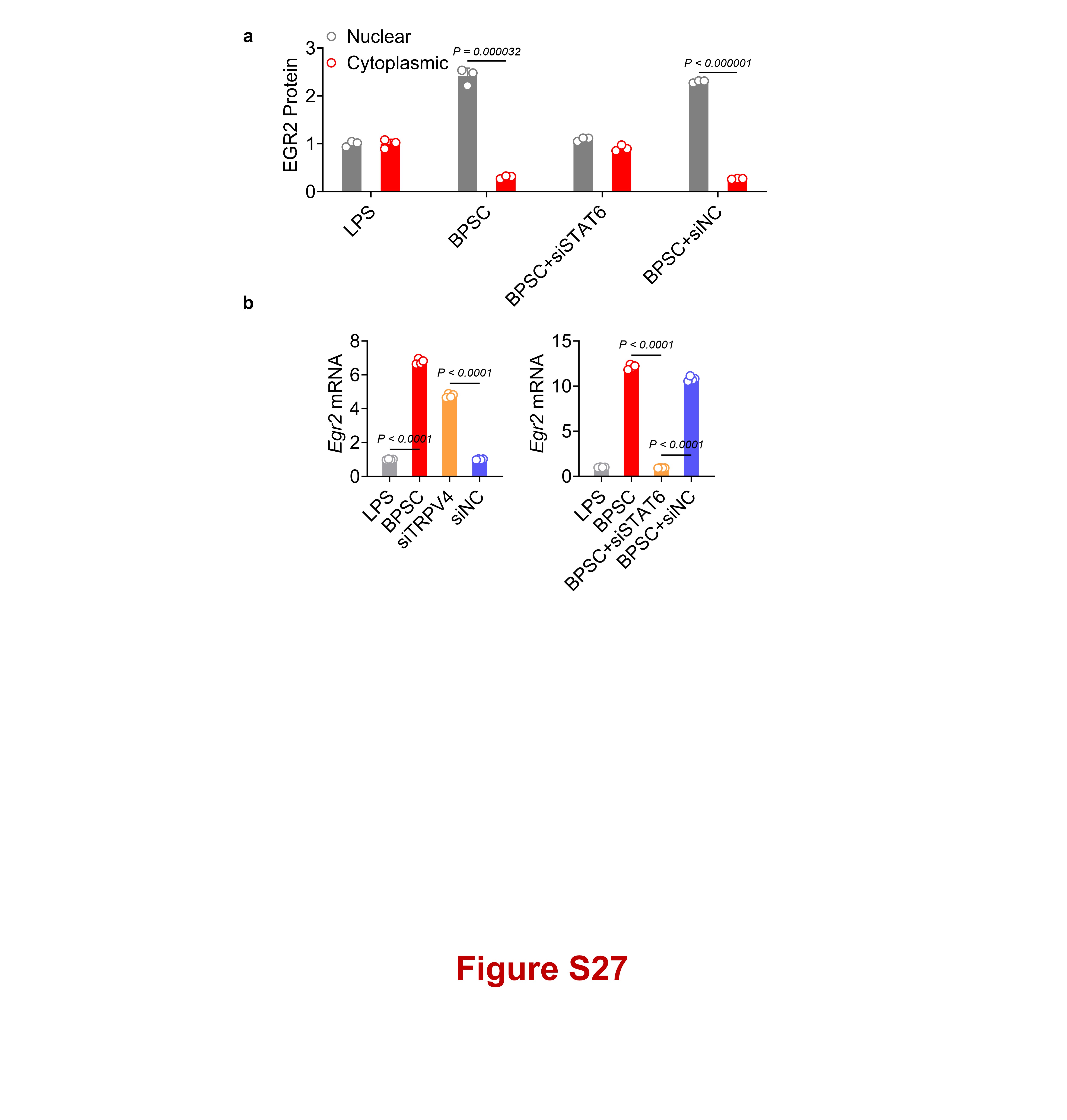
**

**S29:** a)Western blot bands quantification of EGR2 expression in BMDM under near-infrared heat treatment (NIR: 808 nm, 1.25 W/cm², 90 s) (n = 3). b) qPCR analysis of EGR2 mRNA levels in BMDMs under near-infrared heat treatment (NIR: 808 nm, 1.25 W/cm², 90 s)， showing results from two independent knockdown settings, including siTRPV4 (left panel) and siSTAT6 (right panel) groups (n = 4).

**
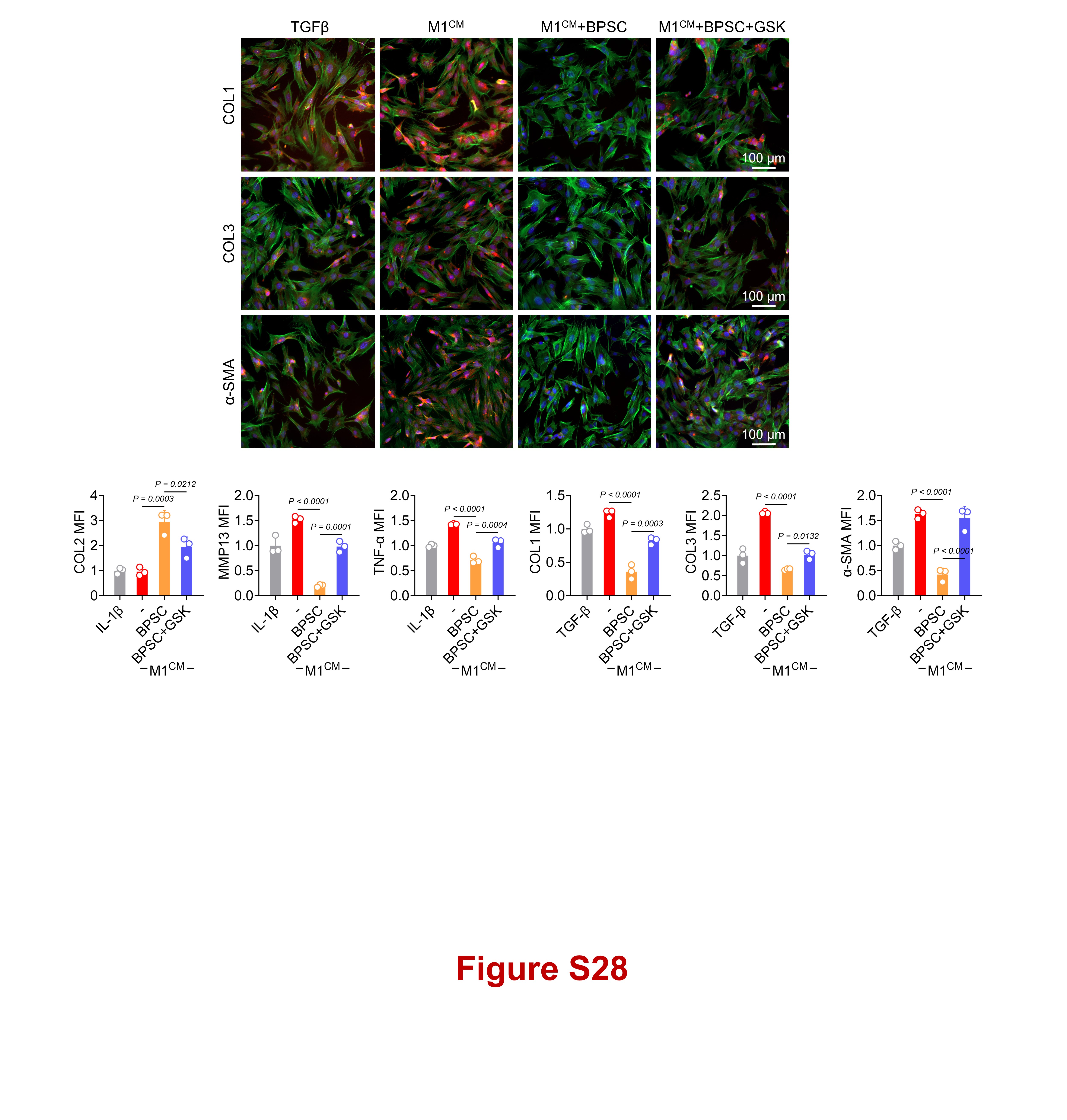
**

**S30:** Immunofluorescence staining and quantification of COL1, COL3, and α-SMA in murine fibroblasts cultured with macrophage-conditioned media from distinct polarization states (n = 3).

**
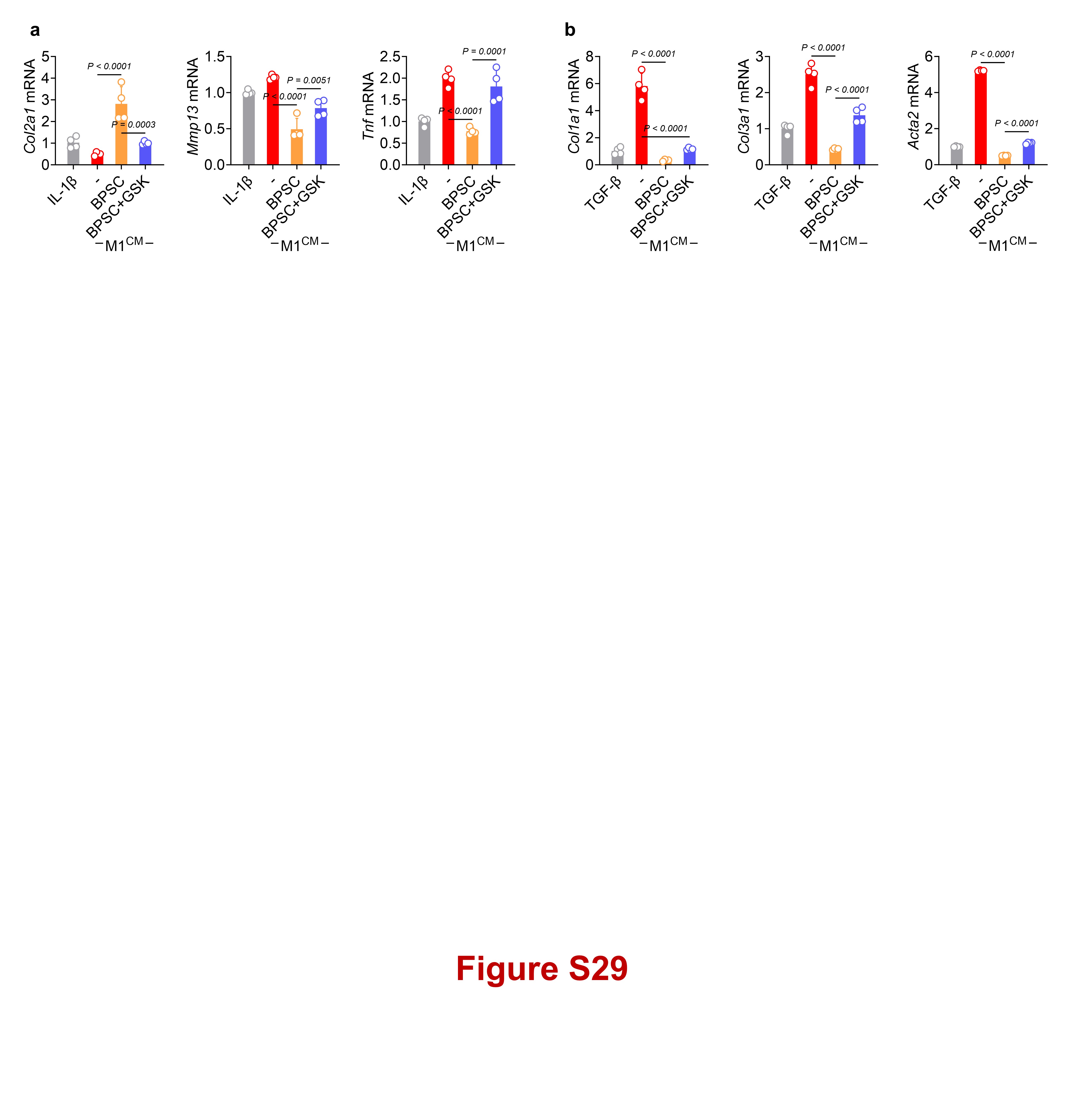
**

**S31:** a) qPCR analysis of *Col2a1*, *Mmp13*, and *Tnf* in Murine chondrocytes cultured with macrophage-conditioned media from distinct polarization states (n = 4). b) qPCR analysis of *Col1*, *Col3*, and *Acta2* in murine fibroblasts cultured with macrophage-conditioned media from distinct polarization states (n = 4).


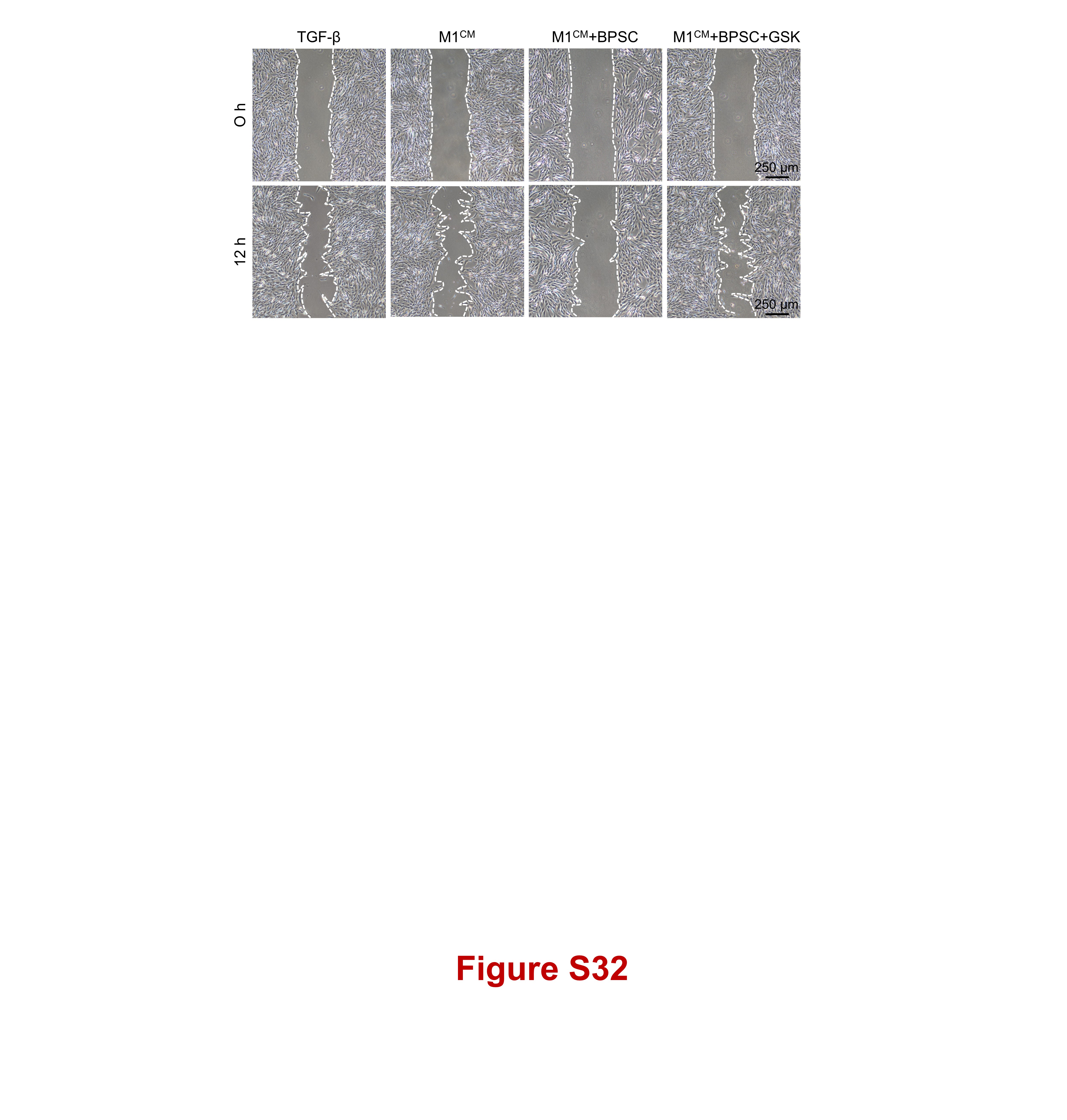


**S32:** Representative scratch wound healing assay and quantitative analysis of murine fibroblasts cultured with macrophage-conditioned media from distinct polarization states with TGFβ treatment.

**
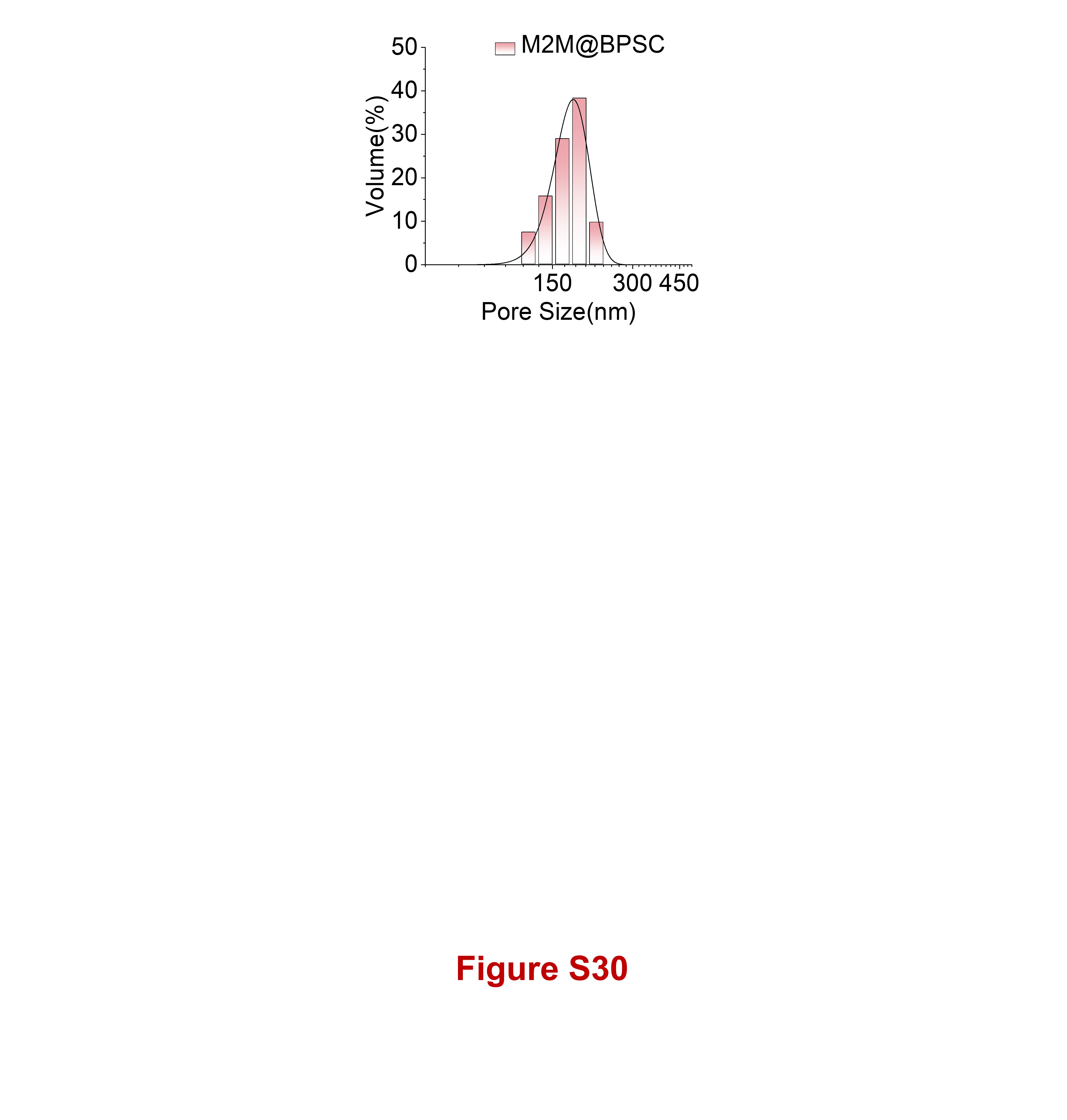
**

**S33:** Size distribution of M2M@BPSC determined by DLS.

**
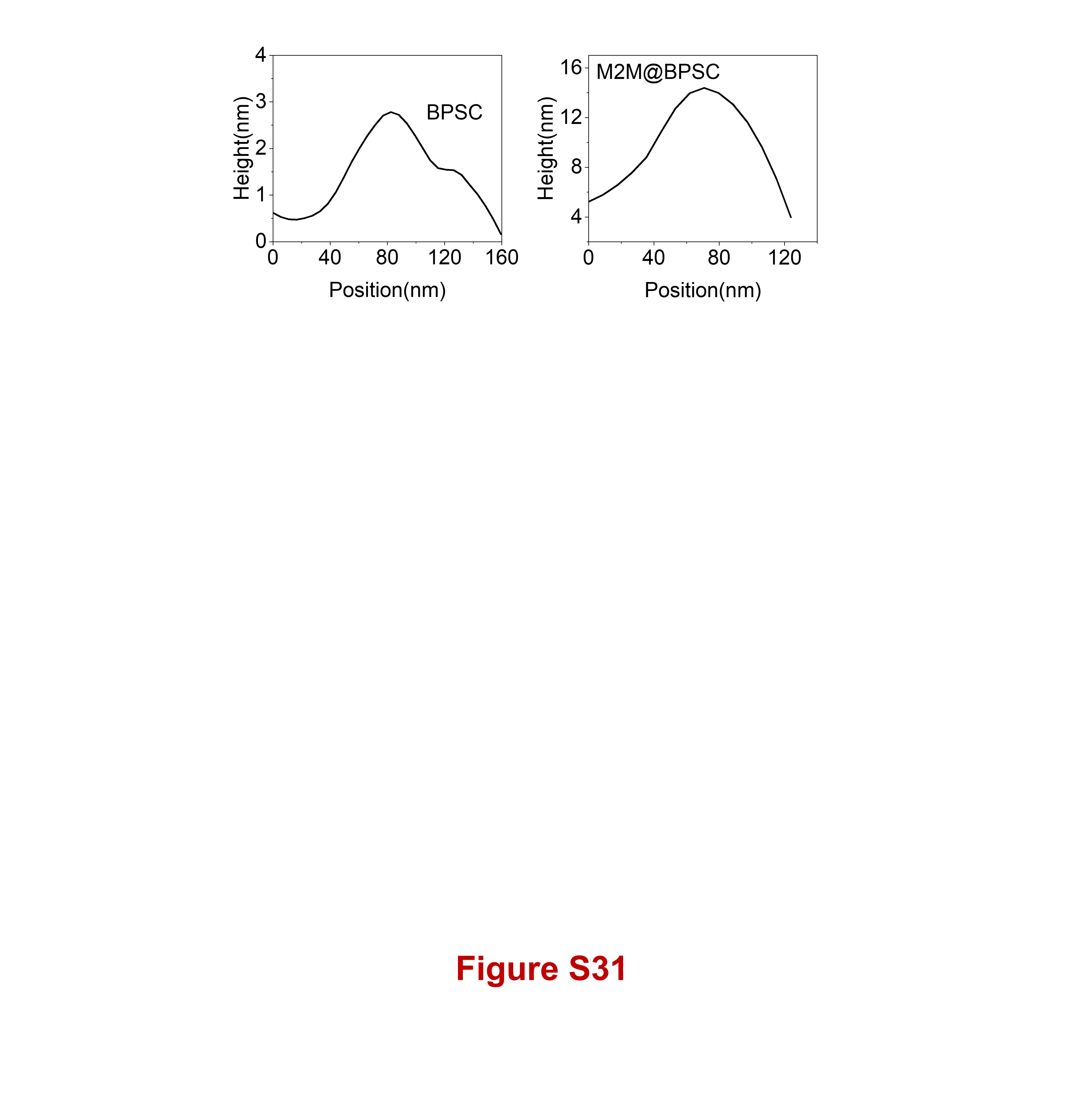
**

**S34:** Height profiles along dashed lines in (Figure 6b).

**
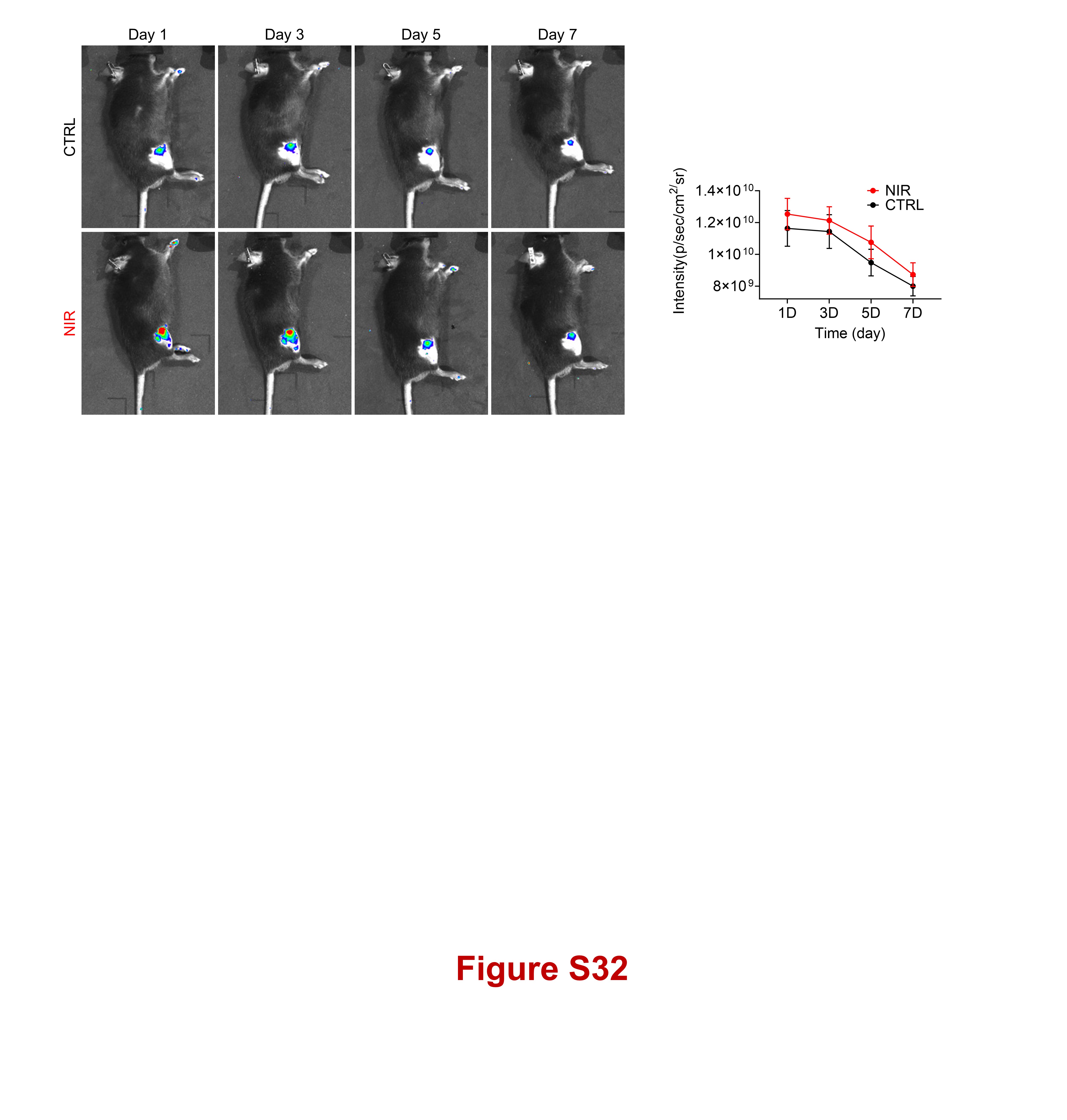
**

**S35:** *In vivo* metabolic infrared imaging and quantitative analysis of M2M@BPSC under control treatment and near-infrared heat treatment (NIR: 808 nm, 1.25 W/cm², 90 s) (n = 3).

**
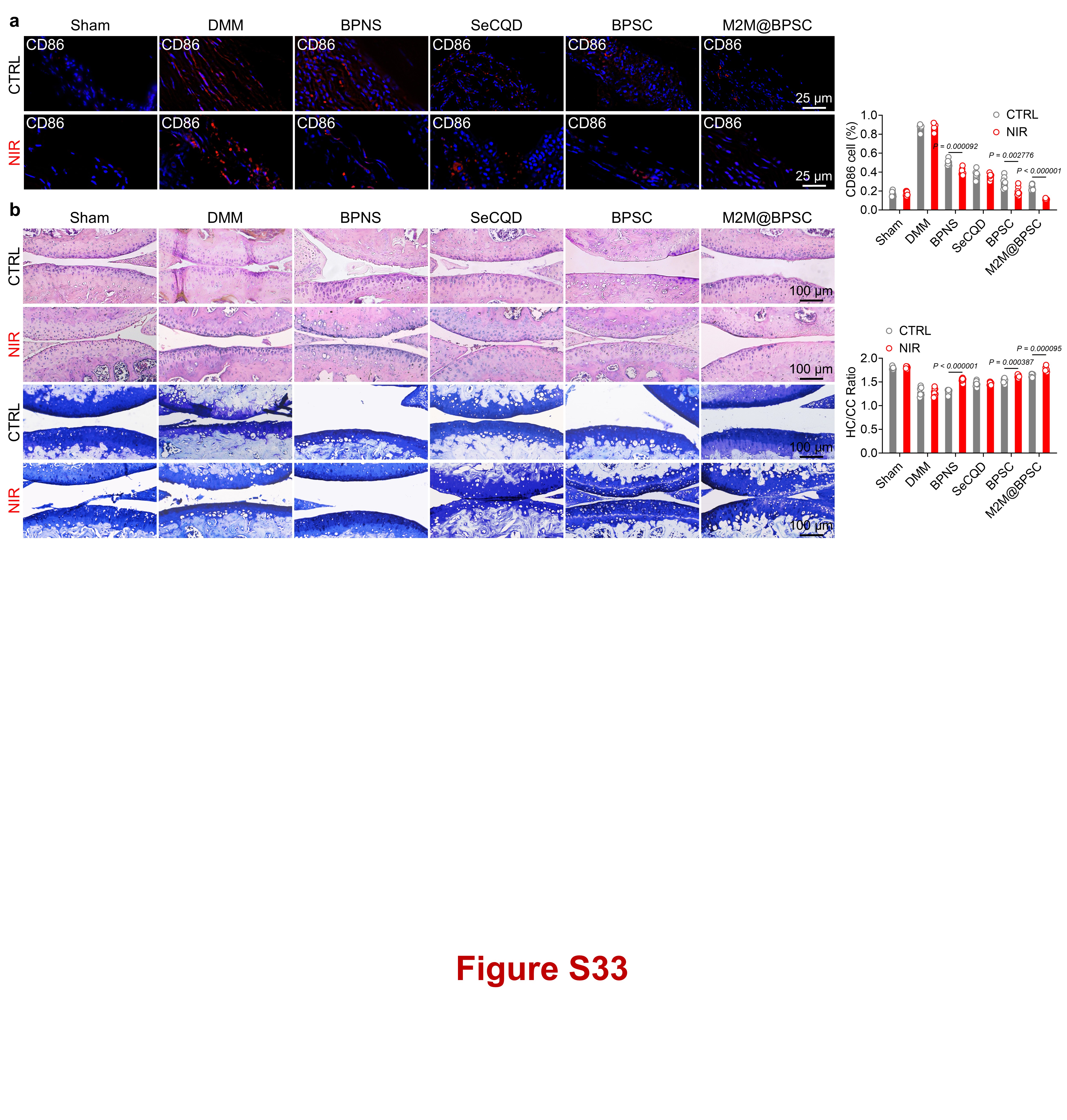
**

**S36:** a) Immunofluorescence staining and quantitative analysis of CD86 (red) and DAPI (blue) in the synovium of mice under control treatment and near-infrared heat treatment (NIR: 808 nm, 1.25 W/cm^2^) (Scale bar: 25 µm) (n = 8). b) H&E, Toluidine Blue staining and quantitative analysis of knee joint sections in mice under control treatment and near-infrared heat treatment (NIR: 808 nm, 1.25 W/cm^2^) (Scale bar: 25 µm) (n = 8).

**
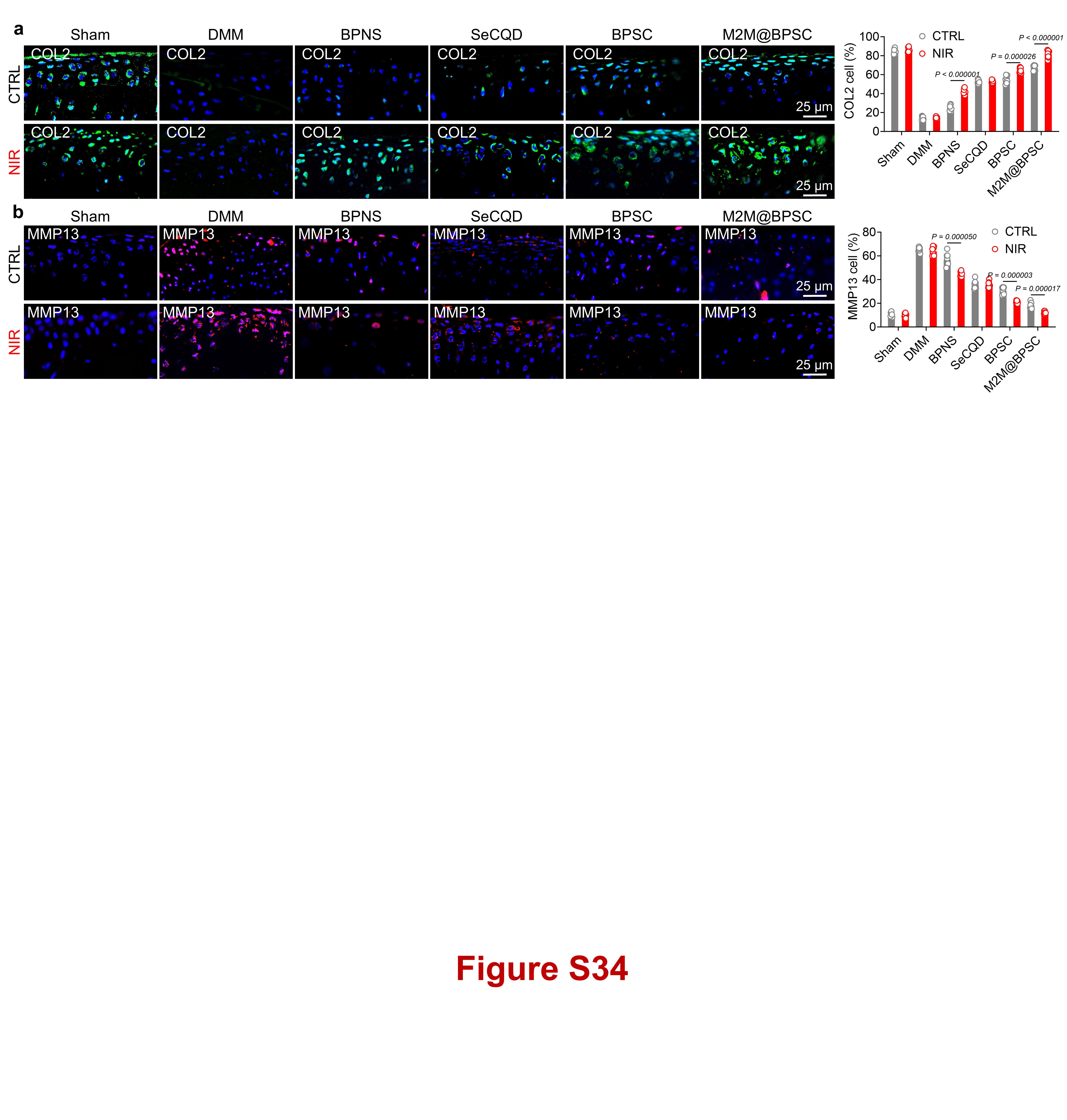
**

**S37:** Immunofluorescence staining and quantitative analysis of COL2 (green), MMP13 (red), and DAPI (blue) in the cartilage of mice under control treatment and near-infrared heat treatment (NIR: 808 nm, 1.25 W/cm^2^) (Scale bar: 25 µm) (n = 8).

**
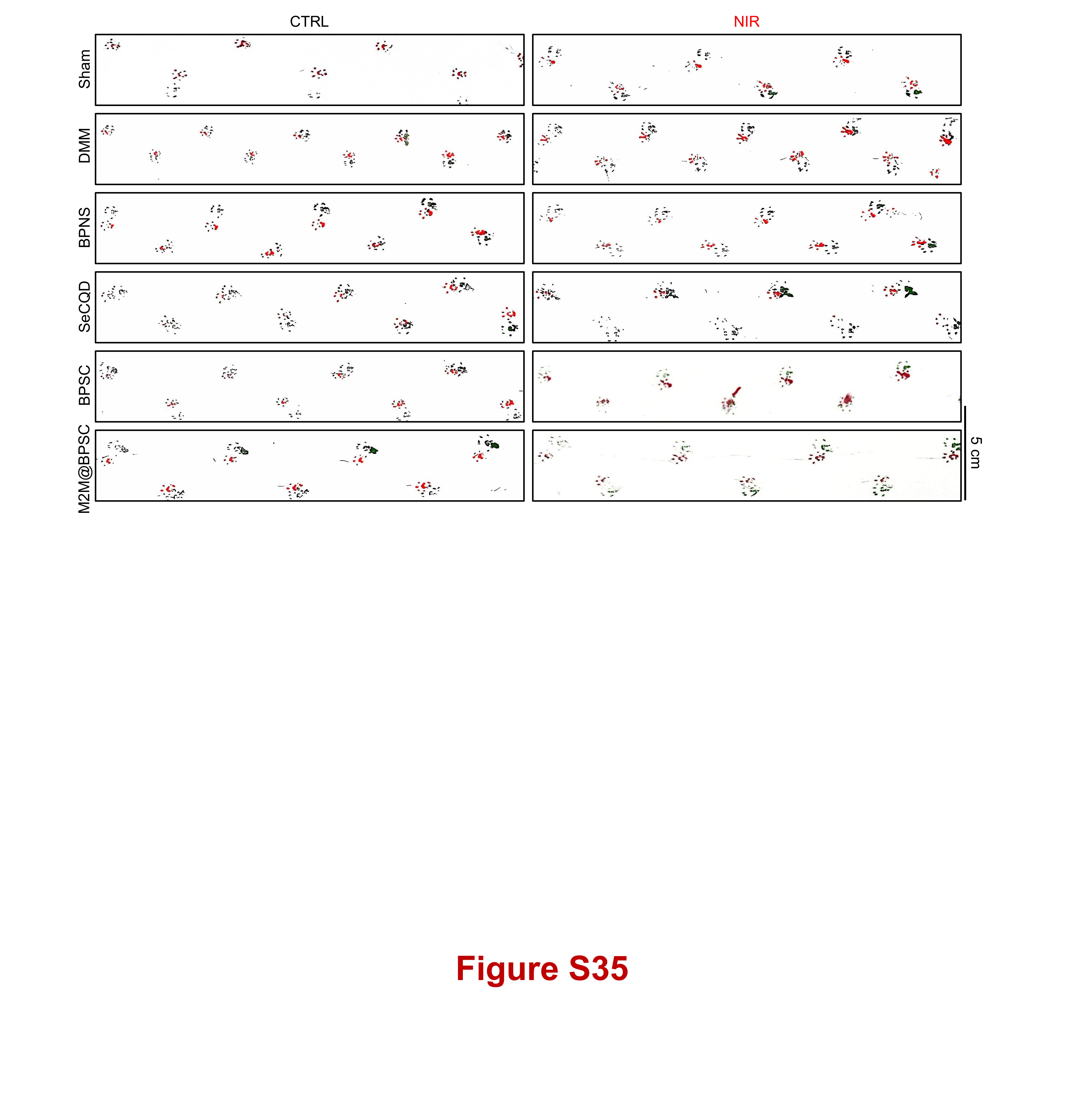
**

**S38:** Gait image of mice under control treatment and near-infrared heat treatment (NIR: 808 nm, 1.25 W/cm^2^) (Scale bar: 25 µm) (n = 8).

**
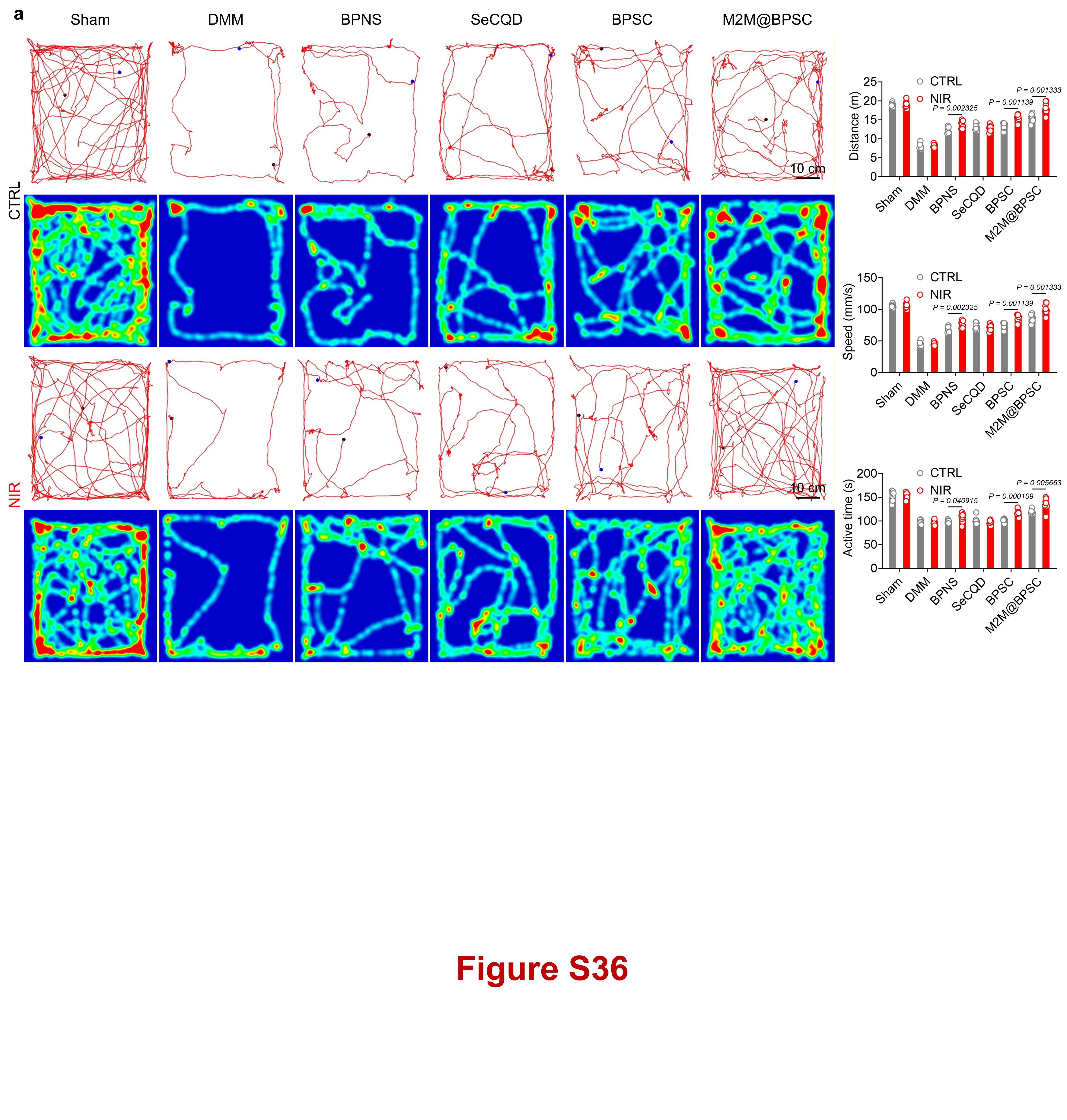
**

**S39:** Representative open field test plots and quantitative analysis of mice under control treatment and near-infrared heat treatment (NIR: 808 nm, 1.25 W/cm^2^) (Scale bar: 25 µm) (n = 5).

**
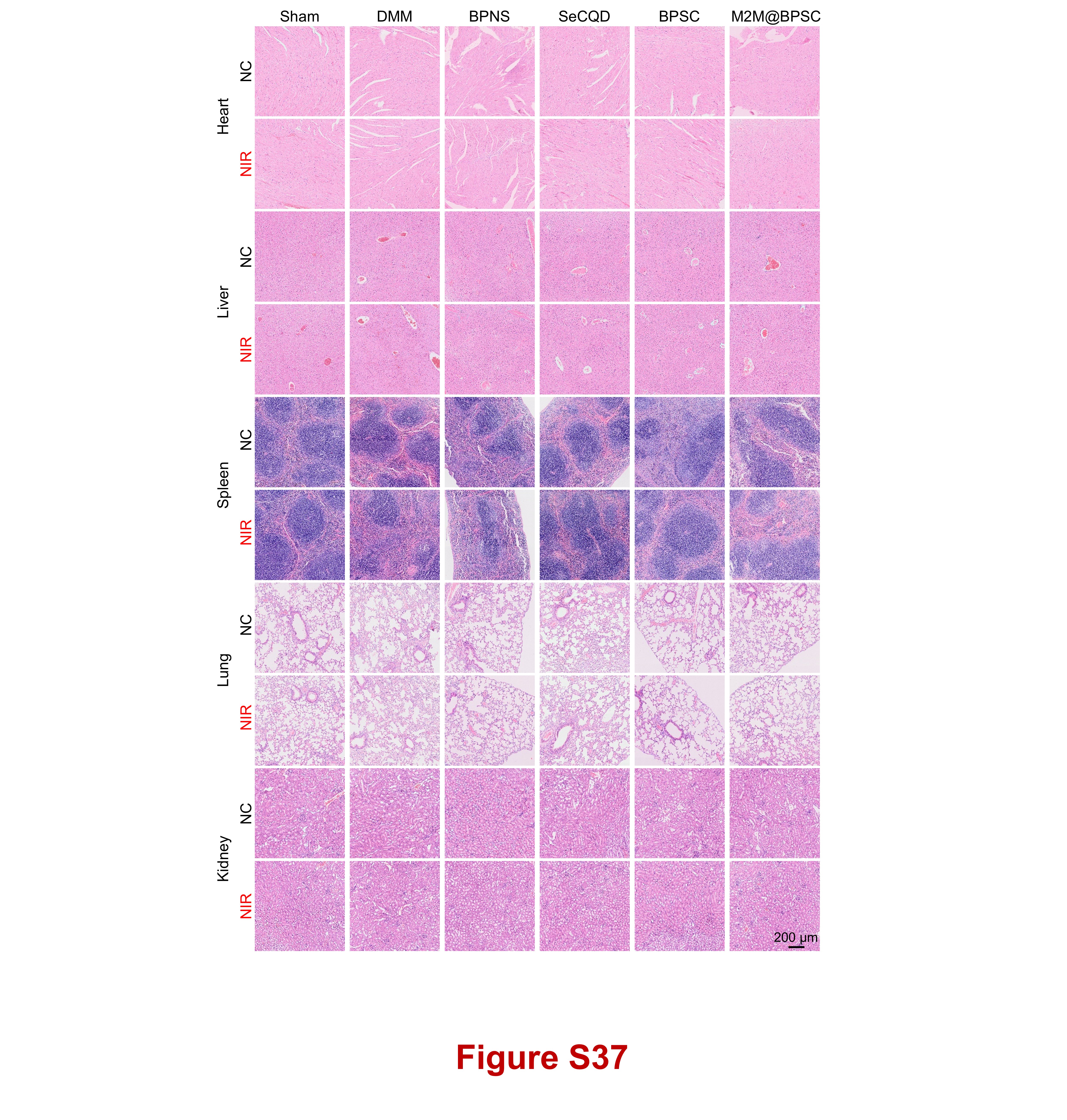
**

**S40:** H&E staining of heart, liver, spleen, lung, and kidney tissues of mice under control treatment and near-infrared heat treatment (NIR: 808 nm, 1.25 W/cm^2^) (Scale bar: 25 µm) (n = 8).

**
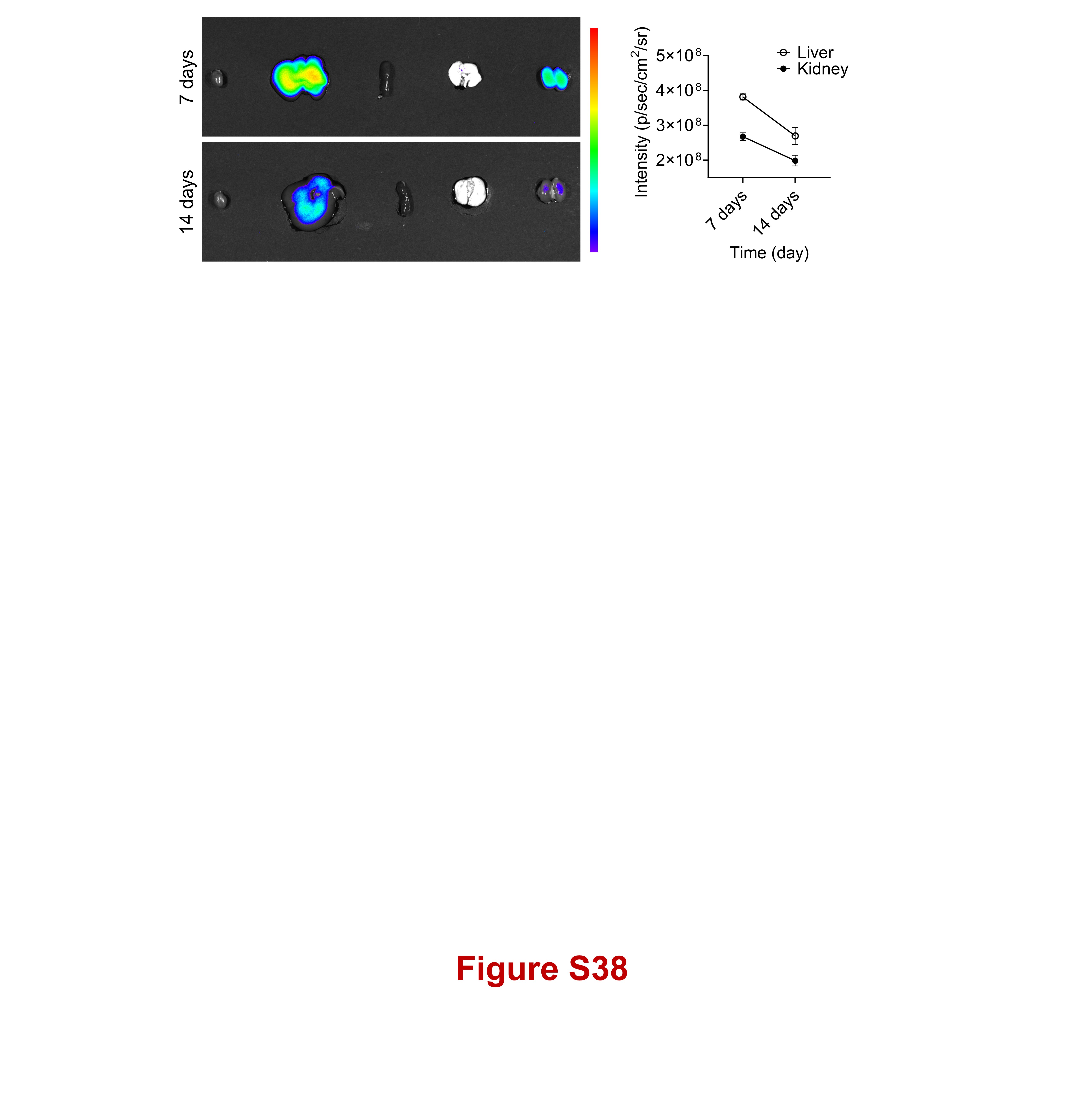
**

**S41:** Representative visceral fluorescence images and quantitative analysis at 7 and 14 days following intra-articular injection of Cy5.5-labeled M2M@BPSC in mice with near-infrared heat treatment (NIR: 808 nm, 1.25 W/cm^2^) (n = 3).

**
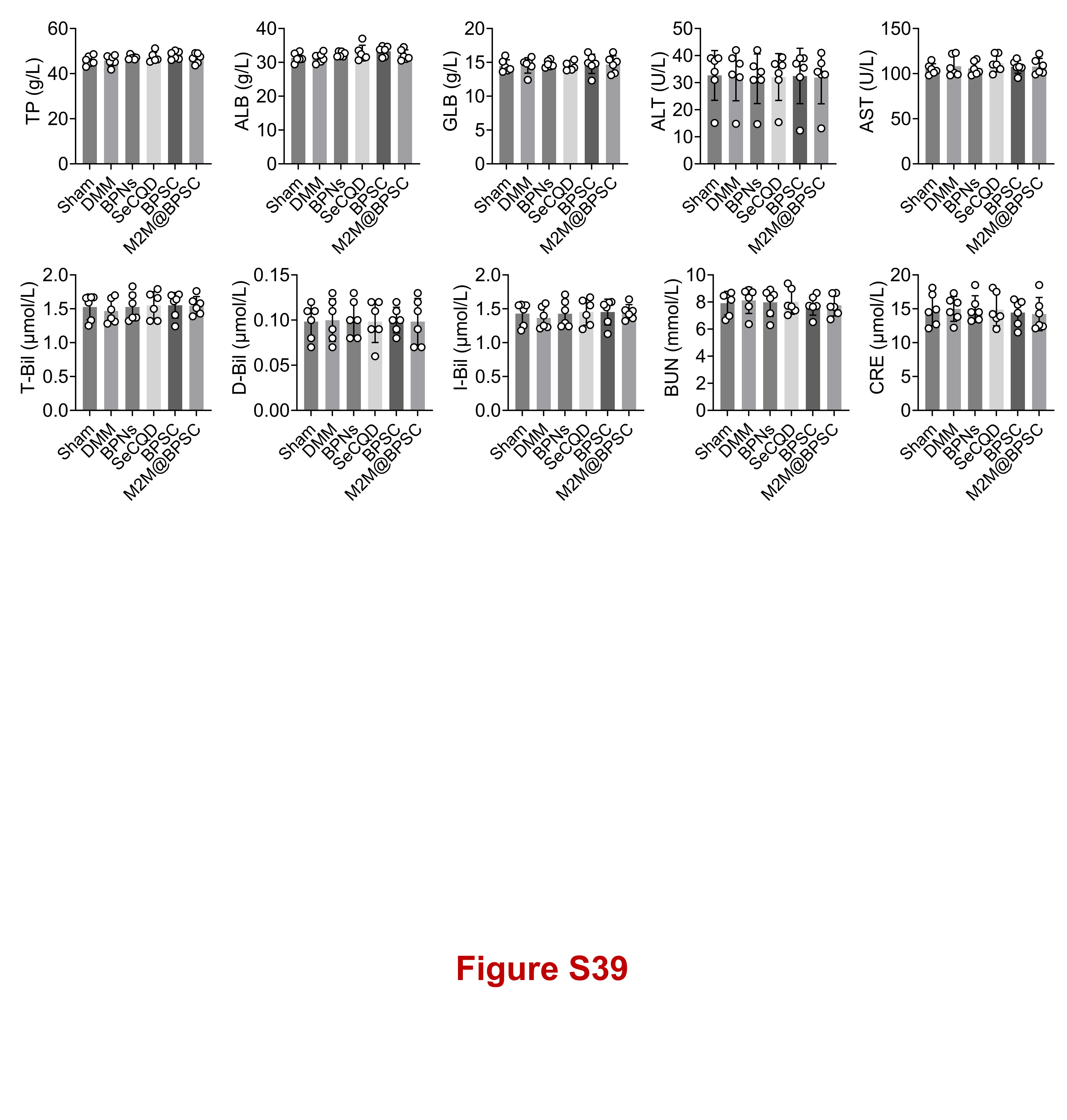
**

**S42:** Serum biochemical parameters of mice, including total protein (TP), albumin (ALB), globulin (GLB), alanine aminotransferase (ALT), aspartate aminotransferase (AST), total bilirubin (T-Bil), direct bilirubin (D-Bil), indirect bilirubin (I-Bil), blood urea nitrogen (BUN), and creatinine (CRE), under near-infrared heat treatment (NIR: 808 nm, 1.25 W·cm⁻²) (n = 6).

**
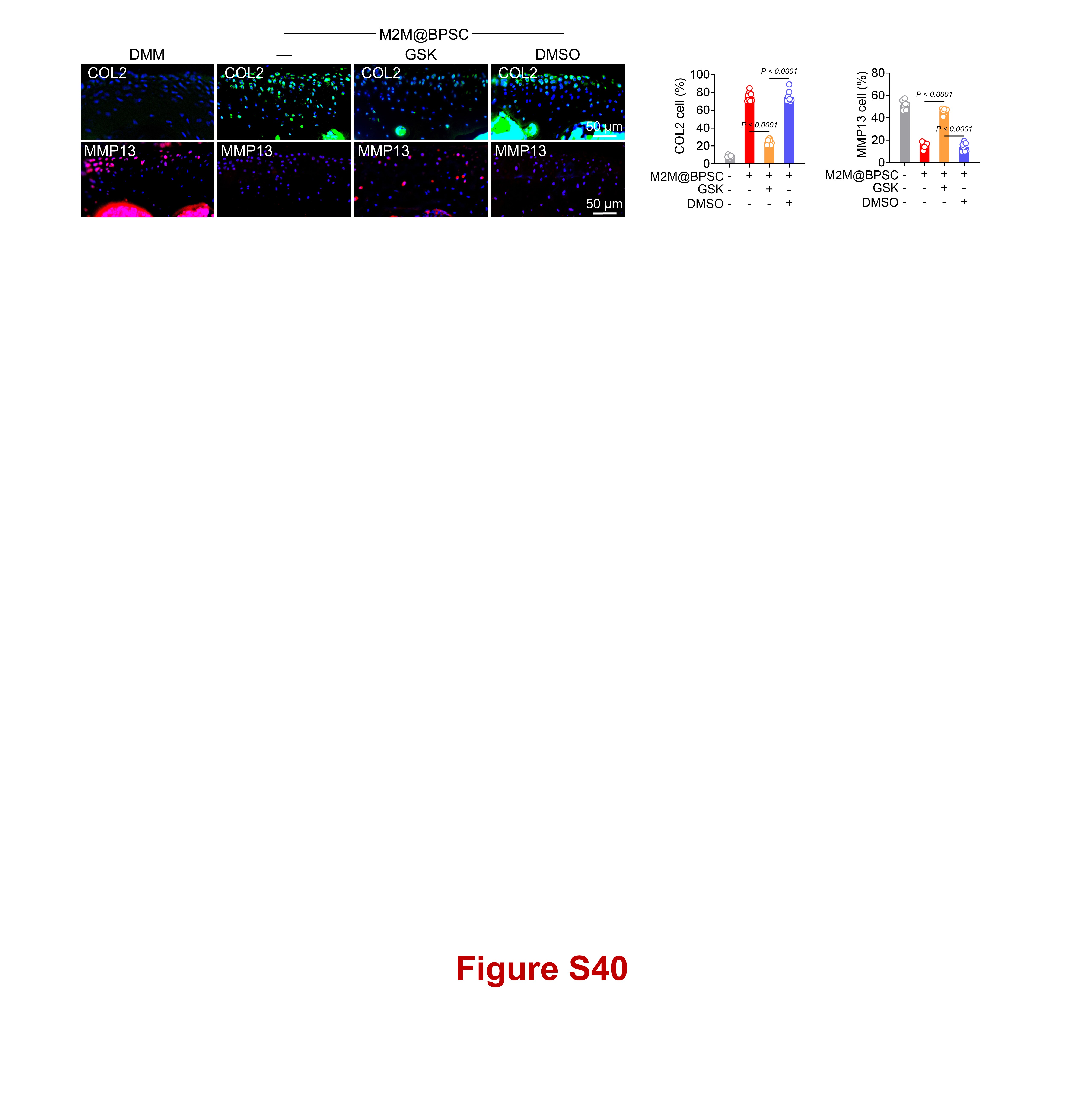
**

**S43:** Immunofluorescence staining and quantitative analysis of COL2 (green), MMP13 (red), and DAPI (blue) in the cartilage of mice under different conditions with near-infrared heat treatment (NIR: 808 nm, 1.25 W/cm^2^) (Scale bar: 25 µm) (n = 8).

**KEY RESOURCES TABLE**

| REAGENT or RESOURCE | SOURCE | IDENTIFIER |  |
| --- | --- | --- | --- |
| **Antibodies** | | |  |
| CD86 Rabbit Monoclonal antibody | ABclonal | Cat#A21198; RRID: AB_3712536 |  |
| CD206 Rabbit Polyclonal antibody | ABclonal | Cat#A8301; RRID: AB_2770422 |  |
| Trpv4 Rabbit Monoclonal antibody | ABclonal | Cat#A22657; RRID: AB_3712533 |  |
| Egr2 Rabbit Monoclonal antibody | ABclonal | Cat#A3219; RRID: AB_2863028 |  |
| Stat6 Rabbit Monoclonal antibody | ABclonal | Cat#A19120; RRID: AB_2862613 |  |
| Phospho-Camk2 Rabbit Monoclonal antibody | ABclonal | Cat#AP1386; RRID: AB_3678648 |  |
| Collagen II Rabbit monoclonal antibody | abcam | Cat#ab188570; RRID: AB_3107030 |  |
| F4/80 Rat Monoclonal Antibody | Huabio | Cat#RT1212; RRID: AB_3712535 |  |
| Mmp13 Rabbit Polyclonal antibody | abcam | Cat#ab39012; RRID: AB_776416 |  |
| Atp5a1 Rabbit Polyclonal antibody | ABclonal | Cat#A11217; RRID: AB_2861524 |  |
| Mt-nd4 Rabbit Polyclonal antibody | ABclonal | Cat#A17970; RRID: AB_2861772 |  |
| Sdha Rabbit Polyclonal antibody | ABclonal | Cat#A2594; RRID: AB_2764479 |  |
| Nos2 Rabbit Polyclonal antibody | ABclonal | Cat#A3774; RRID: AB_3094627 |  |
| Arg1 Polyclonal antibody | ABclonal | Cat#A1847; RRID: AB_2763883 |  |
| Tnfα Rabbit Polyclonal antibody | ABclonal | Cat#A23264; RRID: AB_3665498 |  |
| Collagen I Rabbit Monoclonal antibody | ABclonal | Cat#A21059; RRID: AB_3712537 |  |
| Collagen III Rabbit Monoclonal antibody | ABclonal | Cat#A0817; RRID: AB_3661637 |  |
| αSMA Rabbit Monoclonal antibody | ABclonal | Cat#A17910; RRID: AB_2861755 |  |
| Goat Anti-Rabbit IgG (H+L) Fluor488-conjugated | Affinity | Cat#S0018; RRID: AB_2846215 |  |
| Goat Anti-Rabbit IgG (H+L) 594-conjugated | ABclonal | Cat# AS039; RRID: AB_2768326 |  |
| Goat Anti-Rat IgG (H+L) FITC-conjugated | ABclonal | Cat#AS019; RRID: AB_2769477 |  |
| Polyclonal Rabbit Anti-Histone H3 antibody | ABclonal | Cat#A2348; RRID: AB_2631273 |  |
| Polyclonal Rabbit Anti-beta actin antibody | Servicebio | Cat#GB11001-100; RRID: AB_2801259 |  |
| Goat Anti-Rabbit IgG (H+L) HRP | Affinity | Cat#S0001; RRID: AB_2839429 |  |
| **Chemicals, Peptides, and Recombinant Proteins** | | |  |
| Macrophage Colony-Stimulating Factor (M-CSF) | Novoprotein | Cat#CB34 |  |
| Lipopolysaccharides (LPS) | Sigma-Aldrich | Cat#L2880 |  |
| Recombinant Mouse IL-4 | ABclonal | Cat#RP01161 |  |
| Recombinant Mouse IFN-gamma | ABclonal | Cat#RP01070 |  |
| Recombinant Mouse IL-1β | Novoprotein | Cat#C042 |  |
| Phenylmethanesulfonyl fluoride (PMSF) | Beyotime | Cat#ST506 |  |
| Type II Collagenase | Sigma-Aldrich | Cat# C2-BIOC |  |
| DMEM/F-12 | Keygen BioTECH | Cat# KGL1201-500 |  |
| Fetal Bovine Serum (FBS) | Vazyme Biotech | Cat# F103 |  |
| Penicillin Streptomycin | Gibco | Cat#15140122 |  |
| Paraformaldehyde (PFA) | Servicebio | Cat#G1101 |  |
| Radio Immunoprecipitation Assay Buffer (RIPA buffer) | Beyotime | Cat# P0013E |  |
| SDS Loading Buffer | Beyotime | Cat#P0015 |  |
| Nitrocellulose membrane | Beyotime | Cat#FFN03 |  |
| Western blocking solution | Beyotime | Cat#P0023B |  |
| Western Wash Buffer | Beyotime | Cat#P0023C3 |  |
| YoungPAGE™ Bis-Tris Precast Gradient Gel (4-12%, 8-well×10 gels) | GenScript | Cat#M00938 |  |
| ultra-sensitive Enhanced Chemiluminescent | NCM Biotech | Cat#P10100 |  |
| TRIzol® reagent | Sigma-Aldrich | Cat# T9424 |  |
| ChamQ Blue Universal SYBR qPCR Master Mix | Vazyme | Cat#Q312-02 |  |
| QuickBlock™ Blocking Buffer | Beyotime | Cat#P0228 |  |
| Triton X-100 | Beyotime | Cat#P0096 |  |
| DAPI | Sigma-Aldrich | Cat# D9542 |  |
| FITC Phalloidin | Solarbio | Cat#CA1620 |  |
| PKH26 | MedChemExpress | Cat#367265 |  |
| Trypsin-EDTA | Thermo Fisher Scientific | Cat#25200072 |  |
| Isoflurane | RWD | Cat#R510-22-10 |  |
| Ethylenedinitrilotetraacetic acid (EDTA) | Sigma-Aldrich | Cat#E9884 |  |
| Tissue-Tek O.C.T. Compound | SAKURA | Cat#4583 |  |
| Safranin O | Sigma-Aldrich | Cat#S2255 |  |
| Fast Green FCF | Sigma-Aldrich | Cat#F7252 |  |
| Hematoxylin and Eosin (H&E) | Jiancheng | Cat#D006 |  |
| DiR | MedChemExpress | Cat#HY-D1048 |  |
| **Critical Commercial Assays** |  |  |  |
| HiScript III RT SuperMix for qPCR (+gDNA wiper) | Vazyme | Cat#R323 |  |
| Nuclear and Cytoplasmic Protein Extraction Kit | Beyotime | Cat#P0028 |  |
| BCA Protein Quantification Kit | Vazyme | Cat#E112-01/02 |  |
| Chromatin Immunoprecipitation (ChIP) Kit | Genecreate | Cat#JKR23002A |  |
| Universal DNA Purification and Recovery Kit | Tiangen | Cat#DP214-02 |  |
| DCFH-DA | Medchemexpress | Cat# HY-D0940 |  |
| MitoSOX Red Mitochondrial Superoxide Indicator | Medchemexpress | Cat# HY-D1055 |  |
| Total Antioxidant Capacity (T-AOC) (DPPH) Assay Kit | MLBIO | Cat#ml092650 |  |
| Total Antioxidant Capacity (T-AOC) (ABTS) Assay Kit | MLBIO | Cat#ml092653 |  |
| Hydroxyl Radical Scavenging Capacity Assay Kit | MLBIO | Cat#ml076360 |  |
| Cell Counting Kit-8 | NCM Biotech | Cat#C6005 |  |
| **Experimental Models: Organisms/Strains** |  |  |  |
| C57BL6/J mice | The Jackson Laboratory | RRID: IMSR_JAX:000664 |  |
| **Software and Algorithms** |  |  |  |
| Integrative Genomics Viewer (IGV) | Broad Institute | https://www.broadinstitute.org/ |  |
| UCSC Genome Browser​​ | University of California Santa Cruz | http://genome.ucsc.edu/ |  |
| ChIP-X Enrichment Analysis Version 3(ChEA3) | Icahn School of Medicine at Mount Sinai | https://maayanlab.cloud/chea3/ |  |
| Gene Transcription Regulation Database (GTRD) | N/A | http://gtrd.biouml.org/ |  |
| KnockTF 2.0 | Harbin Medical University | http://www.licpathway.net/KnockTF/index.php |  |
| ChIP-Atlas | Database Center for Life Science | https://chip-atlas.org/ |  |
| TF-Target Finder | N/A | https://jingle.shinyapps.io/TF_Target_Finder/ |  |
| JASPAR 2024 | N/A | https://jaspar.elixir.no/ |  |
| Primer3Plus | N/A | https://www.primer3plus.com/ |  |
| Origin 2021 | OriginLab | https://www.originlab.com/ |  |
| NRecon v1.6 | Bruker | http://www.bruker-microct.com/ |  |
| Dataviewer v1.5.2.4 | Bruker | http://www.bruker-microct.com/ |  |
| CTAn v1.13.8.1 | Bruker | http://www.bruker-microct.com/ |  |
| Mimics Research 19.0 | Materialise | http://www. materialise.com/ |  |
| Prism v9.3.1 | Graphpad | https://www.graphpad.com/;  RRID:SCR_002798 |  |
| Flourish | Flourish Studio | https://flourish.studio/ |  |
| SPSS | IBM | N/A |  |
| Image J | NIH | https://imagej.nih.gov/ij/ |  |

**Supplementary Table 1.** Primers used for qRT-PCR assay

| *Mmu* | Forward Primer sequence (5'-3') | Reverse Primer sequence (5'-3') |
| --- | --- | --- |
| *Gapdh* | AGGTCGGTGTGAACGGATTTG | TGTAGACCATGTAGTTGAGGTCA |
| *Cd86* | TGTTTCCGTGGAGACGCAAG | TTGAGCCTTTGTAAATGGGCA |
| *Cd206* | CTCTGTTCAGCTATTGGACGC | CGGAATTTCTGGGATTCAGCTTC |
| *Nos2* | GTTCTCAGCCCAACAATACAAGA | GTGGACGGGTCGATGTCAC |
| *Arg1* | CTCCAAGCCAAAGTCCTTAGAG | AGGAGCTGTCATTAGGGACATC |
| *Cd209* | CAGTTGAAGGCTGGCGTAGATCG | GTGGCAGGCAGTGGCAGAATC |
| *Cd80* | CAACTGTCCAAGTCAGTGAAAG | CACCACTTTGTCATGTTTTTGC |
| *Tnf* | ATGTCTCAGCCTCTTCTCATTC | GCTTGTCACTCGAATTTTGAGA |
| *Col2a1* | TACTGGAGTGACTGGTCCTAAG | AACACCTTTGGGACCATCTTTT |
| *Mmp 13* | CTTCCTGATGATGACGTTCAAG | GTCACACTTCTCTGGTGTTTTG |
| *Col3a1* | GAAAGAATGGGGAGACTGGAC | TACCAGGTATGCCTTGTAATCC |
| *Col1a1* | TGAACGTGGTGTACAAGGTC | CCATCTTTACCAGGAGAACCAT |
| *Acta2* | GTCCCAGACATCAGGGAGTAA | TCGGATACTTCAGCGTCAGGA |
| *Trpv4* | GGACCTACAGCATCATGATTCA | GATCATGAAGAGCAGGTACACA |
| *Sdha* | GGAACACTCCAAAAACAGACCT | CCACCACTGGGTATTGAGTAGAA |
| *Mt-nd4* | CTCCTCAGACCCCCTATCCA | AAATCCCTGCGTTTAGGCGT |
| *Atp5f1a* | TCTCCATGCCTCTAACACTCG | CCAGGTCAACAGACGTGTCAG |
| *Stat6* | CTGAGGCACCCTGTATATCCC | GGCAGAAAGTAGGGCACTGG |
| *Egr2* | GATCACAGGCAGGAGAGACTG | TCGGATACGGGAGATCCAGG |
| *Irf4* | GCCAGCCCAGGTTCATAACTA | CTCAATGGGGATTCCAGGAGC |
| *Myc* | ATGCCCCTCAACGTGAACTT | CGACCGCAACATAGGATGGA |
| *Nr4a1* | GGCAGAGACGCGAGTG | AACAACTCACAACATCCCCTC |
| *Jun* | GGGAGCATTTGGAGAGTCCC | TTTGCAAAAGTTCGCTCCCG |
| *Fos* | TACTACCATTCCCCAGCCGA | GCTGTCACCGTGGGGATAAA |

**Supplementary Table 2.** Primers used for ChIP qPCR assay

| Site | Forward Primer sequence (5'-3') | Reverse Primer sequence (5'-3') |
| --- | --- | --- |
| Site1 | AACAAACAGCCCAGACCTGT | ACGGAAGCTGACTGCCTTTT |
| Site2 | TCCTGGGGTAGACCTTAGGC | TGGCGACAGTCAGGATATCC |
| Site3 | CAGCGCTCGGTTTTCTTTCC | GTAGCTCTGTCTCTTGGGGC |

**Supplementary Table 3. siRNA sequences used for gene knockdown**

| Target gene | siRNA name | Sense (5′→3′) | Antisense (5′→3′) |
| --- | --- | --- | --- |
| *Trpv4* | *Trpv4-Mus-1466* | GAGCCCAUUAACGAACUGUTT | ACAGUUCGUUAAUGGGCUCTT |
|  | *Trpv4-Mus-1700* | GACUUGUUCACGAAGAAAUTT | AUUUCUUCGUGAACAAGUCTT |
|  | *Trpv4-Mus-458* | CCCAGUGACAACAAGAGAUTT | AUCUCUUGUUGUCACUGGGTT |
| *Stat6* | *Stat6-Mus-1776* | CCUGGUCACAGUUCAAUAATT | UUAUUGAACUGUGACCAGGTT |
|  | *Stat6-Mus-2722* | CCUGCAACCAUCUCCUUAUTT | AUAAGGAGAUGGUUGCAGGTT |
|  | *Stat6-Mus-1469* | GCCCCAACAAACUUCUCAUTT | AUGAGAAGUUUGUUGGGGCTT |
